# Genetic dissection of main and epistatic effects of QTL based on augmented triple test cross design

**DOI:** 10.1371/journal.pone.0189054

**Published:** 2017-12-14

**Authors:** Xueli Zhang, Congwei Sun, Zheng Zhang, Zhijun Dai, Yuan Chen, Xiong Yuan, Zheming Yuan, Wenbang Tang, Lanzhi Li, Zhongli Hu

**Affiliations:** 1 Hunan Engineering & Technology Research Center for Agricultural Big Data Analysis & Decision-Making, Hunan Agricultural University, Changsha, Hunan, China; 2 Hunan Province Collaborative Innovation Center for the Key Agriculture Pest Control, Hunan Agricultural University, Changsha, Hunan, China; 3 Rice Research Institute, Hunan Agricultural University, Changsha, Hunan, China; 4 State Key Laboratory of Hybrid Rice, Hubei Lotus Engineering Center, College of Life Sciences, Wuhan University, Wuhan, Hubei, China; New Mexico State University, UNITED STATES

## Abstract

The use of heterosis has considerably increased the productivity of many crops; however, the biological mechanism underpinning the technique remains elusive. The North Carolina design III (NCIII) and the triple test cross (TTC) are powerful and popular genetic mating design that can be used to decipher the genetic basis of heterosis. However, when using the NCIII design with the present quantitative trait locus (QTL) mapping method, if epistasis exists, the estimated additive or dominant effects are confounded with epistatic effects. Here, we propose a two-step approach to dissect all genetic effects of QTL and digenic interactions on a whole genome without sacrificing statistical power based on an augmented TTC (aTTC) design. Because the aTTC design has more transformation combinations than do the NCIII and TTC designs, it greatly enriches the QTL mapping for studying heterosis. When the basic population comprises recombinant inbred lines (RIL), we can use the same materials in the NCIII design for aTTC-design QTL mapping with transformation combination Z_1_, Z_2_, and Z_4_ to obtain genetic effect of QTL and digenic interactions. Compared with RIL-based TTC design, RIL-based aTTC design saves time, money, and labor for basic population crossed with F_1_. Several Monte Carlo simulation studies were carried out to confirm the proposed approach; the present genetic parameters could be identified with high statistical power, precision, and calculation speed, even at small sample size or low heritability. Additionally, two elite rice hybrid datasets for nine agronomic traits were estimated for real data analysis. We dissected the genetic effects and calculated the dominance degree of each QTL and digenic interaction. Real mapping results suggested that the dominance degree in Z_2_ that mainly characterize heterosis showed overdominance and dominance for QTL and digenic interactions. Dominance and overdominance were the major genetic foundations of heterosis in rice.

## Introduction

Heterosis, or hybrid vigor, describes the superior performance of heterozygous hybrid plants over their homozygous parental inbred lines [1–3]. The development of heterotic crops, especially those for hybrid rice and maize, is one of the most important applications of genetics in agriculture [4–5], but the molecular basis underlying heterosis remains elusive.

Indeed, much of our knowledge regarding heterosis derives from classical genetic studies on maize, during which the fundamental hypotheses for heterosis were defined, with the main competing hypotheses including dominance, overdominance, and epistasis [1,5–6]. The dominance hypothesis explains heterosis by the complementing action of superior dominant alleles from both parental inbred lines at multiple loci over the corresponding unfavorable alleles leading to the improved vigor of hybrid plants [1,5,7–8]. The overdominance hypothesis attributes heterosis to allelic interactions at one or multiple loci in hybrids that result in superior traits compared to the homozygous parental inbred lines [1,9–10]. In addition, the epistasis hypothesis considers epistatic interactions between non-allelic genes at two or more loci as the main factor for the superior phenotypic expression of a trait in hybrids [1,10–12].

To decipher the genetic basis of heterosis, NCIII [13] and TTC [14] are powerful genetic mating designs widely used in maize [12,15–19], rice [12,8,20–26], and *Arabidopsis thaliana* [27–30]. Rice is the staple food for a large segment of the world’s population. The success of hybrid rice breeding [31], together with its relatively small genome size [32], saturated molecular linkage maps [33], and rapid advances in genome sequencing [34–35], have provided a novel opportunity for dissecting the genetic basis of heterosis.

In Xiao et al.’s study [8], based on the NCIII design, 194 F_7_ RIL were backcrossed to their parental lines to develop the mapping population, and 37 QTL were detected for 12 quantitative traits by single-point analysis [one-way analysis of variance (ANOVA)] and an interval mapping method. In one of the two BC_1_F_7_ populations, 82% of the detected heterozygotes were superior to the respective homozygotes; therefore, Xiao et al. concluded that dominance complementation was the major genetic basis of heterosis in rice. On the other hand, Li et al. [21] and Luo et al. [22] investigated five interrelated mapping populations by an interval mapping method in which 254 F_10_ RIL were selected as the base population; two BC_1_F_1_ populations were derived from the NCIII design and two test cross populations were obtained by test crossing the RIL with two testers (Zhong 413 and IR64). The results suggested that epistasis and overdominance, rather than dominance, were the major genetic bases of heterosis in rice. Yu et al. [20] also pointed out that epistasis played a major role as the genetic basis of heterosis. Hua et al. [23] investigated the genetic components conditioning the heterosis of yield and yield component traits in an elite rice hybrid using an immortalized F_2_ population with modified composite interval mapping (CIM) and two-way ANOVA methods and found that heterotic effects at the single-locus level and a dominance × dominance interaction at the two-locus level could adequately explain the genetic basis of heterosis. In our previous study [24] based on the NCIII design, two recombinant inbred populations were backcrossed to their respective parents to develop mapping populations (L_1_ and L_2_) in which main-effect QTL were detected by the CIM method and epistatic QTL were detected by the mixed linear approach in the RIL population and summation (L_1_ + L_2_) and subtraction (L_1_ − L_2_) data of two backcross populations. The research demonstrated that heterosis was attributable to the orchestrated outcome of partial-to-complete dominance, overdominance, and epistasis. In addition, based on an ultra-high-density single nucleotide polymorphism bin map constructed with population sequencing, the immortalized F_2_ population in Hua et al. [23] was reanalyzed by Zhou et al. [26] with an *h* test in one-locus effects detection and two-way ANOVA in two-locus interactions for the whole genome. The results suggested that relative contributions of the genetic components varied with traits; single-locus dominance had relatively small contributions in all of the traits and the cumulative effects of these different components may adequately explain the genetic basis of heterosis. This conclusion was consistent with our previous study [24].

In summary, most of the mapping populations above derive from the NCIII design, and QTL mapping methods usually employ ANOVA, interval mapping, or the CIM method in one-locus effects detection and two-locus interactions. However, the estimated additive and dominant effects are confounded with epistatic effect if epistasis is present. Kao and Zeng [36] pointed out that a two-way ANOVA-exploiting genetic marker and trait phenotype data from an F_2_-segregating population was, in principle, inappropriate for testing for pairwise epistasis, even though this approach has been widely used in analyses of such data sets [20–21,23]. In addition, only one variable was involved in the model at one time, which was not able to capture all types of genetic effects, especially epistatic effects, simultaneously on the whole genome. In 2008, Garcia et al. [25] developed a multiple-interval mapping model for the NCIII design that provided a platform to simultaneously estimate the number, genomic positions, augmented additive and dominance effects, and digenic interactions (*aa* + *dd* and *ad* + *da*) of QTL. This method was used to reanalyze the datasets by Stuber et al. [12], who found that additive × additive effect (*aa*) epistatic effects of QTL could be the main cause for the heterosis in rice. After this, He et al. [37] proposed a method for mapping epistatic QTL associated with heterosis using the RIL-based NCIII design by a series of simulation studies; however, main or epistatic effects were mixed measured as augment effects.

In 1988, Liu [38] proposed an aTTC design based on the TTC design. In TTC design, base populations are backcrossed to *P*_*1*_, *P*_*2*_ and *F*_*1*_ to get *L*_*1i*_, *L*_*2i*_, and *L*_*3i*_, *i* = 1…n, whereas in aTTC design, base populations are simultaneously self-mated to get *L*_*4i*_. The aTTC design provided several ways to detect epistasis by detecting a variance component. However, there was no report based on aTTC design for QTL mapping on a Mendelian factor level.

In this paper, under aTTC design, based on four data sets (*L*_*1i*_, *L*_*2i*_, *L*_*3i*_, and *L*_*4i*_), we developed six data set transformations [38]: *Z*_1*i*_ = *L*_1*i*_ + *L*_2*i*_, *Z*_2*i*_ = *L*_1*i*_ − *L*_2*i*_, *Z*_3*i*_ = *L*_1*i*_ + *L*_2*i*_ − 2*L*_3*i*_, *Z*_4*i*_ = *L*_1*i*_ + *L*_2*i*_ − *L*_4*i*_, *Z*_5*i*_ = *L*_1*i*_ + *L*_2*i*_ + *L*_3*i*_, and *Z*_6*i*_ = 2*L*_3*i*_ − *L*_4*i*_. By employing Z_1_, Z_2_, and Z_3_, He and Zhang [39] provided a complete solution for dissecting main and epistatic effects in the F_2_-based TTC design through a simulation study. Our study utilized different data set combinations (Z_1_, Z_2_, and Z_4_), (Z_1_, Z_2_, and Z_5_), and (Z_1_, Z_2_, and Z_6_), respectively, to provide a two-step approach for estimating, in an unambiguous and unbiased manner, all the main and, especially, epistatic effects of QTL; this method also fits for many types of base populations such as RIL, F_2_, and Double Haploid (DH). Here, we will take the first combination (Z_1_, Z_2_, and Z_4_) of the RIL-based aTTC design as an instance for QTL mapping to dissect genetic effects. The other combinations (Z_1_, Z_2_, and Z_5_) and (Z_1_, Z_2_ and Z_6_) listed above can also be used to estimate genetic effects. A series of Monte Carlo simulation studies were carried out to confirm the proposed approach. We further applied the proposed method to real data analysis.

## Materials and methods

### Genetic design

In aTTC design, F_2_ populations or their offspring (BC, DH, or RIL) derived from the hybridization of two pure lines (*P*_*1*_ and *P*_*2*_) and were selected as the base population. On one hand, *n* individuals in the base population were crossed to three testers (*P*_*1*_, *P*_*2*_, and *F*_*1*_) to get *L*_*1i*_, *L*_*2i*_, and *L*_*3i*_, respectively (*i* = 1, 2… n); on the other hand, the *n* individuals in the base population were self-mated to get *L*_*4i*_. Therefore, *4n* aTTC lines (*L*_*1i*_, *L*_*2i*_, *L*_*3i*_, and *L*_*4i*_) can be obtained and used for the detection of epistasis.

All *4n* families, each with *m* replications, were planted. Molecular marker information was observed from all of the *n* base population lines and the testers P_1_, P_2_ and F_1_, whereas quantitative traits were measured for all *4nm* aTTC progeny. The phenotypic observations were denoted by *y*_*tij*_, where *t* = 1, 2, 3, and 4 for *L*_*1i*_, *L*_*2i*_, *L*_*3i*_, and *L*_*4i*_; respectively; *j* = 1, 2… m. The family means were denoted by ${\bar{L}}_{ti}=\sum_{j=1}^{m}y_{tij}/m$.

The genetic expectations of six data set transformations, Z_1i_, Z_2i_, Z_3i_, Z_4i_, Z_5i_, and Z_6i_, were obtained from *L*_*1i*_, *L*_*2i*_, *L*_*3i*_, and *L*_*4i*_. *Z*_1*i*_ = *L*_1*i*_ + *L*_2*i*_, *Z*_2*i*_ = *L*_1*i*_ − *L*_2*i*_, *Z*_3*i*_ = *L*_1*i*_ + *L*_2*i*_ − 2*L*_3*i*_, *Z*_4*i*_ = *L*_1*i*_ + *L*_2*i*_ − *L*_4*i*_, *Z*_5*i*_ = *L*_1*i*_ + *L*_2*i*_ + *L*_3*i*_, and *Z*_6*i*_ = 2*L*_3*i*_ − *L*_4*i*_. Two main metrics were adopted for the *4n* aTTC lines: the F_∞_ and F_2_ metrics [36,40]; their genetic expectations are listed in S1 Supporting Information.

### Genetic models for mapping heterotic QTL in RIL-based aTTC design

The derivation of the expected genetic values of Z_1i_, Z_2i_, Z_3i_, Z_4i_, Z_5i_, and Z_6i_ under both the F_∞_ and F_2_ metric models is presented in S3 Supporting Information under the assumption that the quantitative trait was determined by two QTL with digenic epistasis and arbitrary linkage. The genetic effect symbols adopted in this research were described by Kao and Zeng [36]. He et al. [37] simulated and estimated main and epistatic QTL in the RIL-based NCIII design under both the F_∞_ and F_2_ metrics models and found that QTL mapping results under the F_∞_ metric were superior to the F_2_ metric; therefore, this paper simulated QTL under the F_∞_ metric models.

#### QTL mapping models in the RIL-based aTTC design under the F_∞_ metric model

The phenotypic values of Z_1i_ and Z_2i_ in the RIL-based aTTC design are the same as the RIL-based NCIII design. Details can be found in the publication by He et al. [37]. According to the genetic expectations of Z_1i_ under the F_∞_ metric model (Table A5 in S1 Supporting Information), the phenotypic value of Z_1i_ can be described as

$$Z_{1i}=2\mu +x_{a_{1}i}a_{1}+d_{1}+x_{a_{2}i}a_{2}+d_{2}+x_{a_{1}a_{2}i}i_{a_{1}a_{2}}+x_{a_{1}d_{2}i}i_{a_{1}d_{2}}+x_{d_{1}a_{2}i}i_{d_{1}a_{2}}+x_{d_{1}d_{2}i}i_{d_{1}d_{2}}+e_{1i},$$ (1)

where *μ* is the mean genotypic value of the four homozygotes in the RIL population; *a*_*k*_ and *d*_*k*_ are additive and dominance effects of the *k*th QTL (*k* = 1, 2); $i_{a_{1}a_{2}}$, $i_{a_{1}d_{2}}$, $i_{d_{1}a_{2}}$, and $i_{d_{1}d_{2}}$ are *additive* × *additive*, *additive* × *dominance*, *dominance* × *additive*, and *dominance* × *dominance* interactions between two QTL, respectively; $x_{a_{1}i}$, $x_{a_{2}i}$, $x_{a_{1}a_{2}i}$, $x_{a_{1}d_{2}i}$, $x_{d_{1}a_{2}i}$, and $x_{d_{1}d_{2}i}$ are dummy variables and are determined by the genotype of the *i*th RIL line (Table A5 in S1 Supporting Information); and *e*_1*i*_ is the residual error with an $N(0,\sigma_{1}^{2})$ distribution. According to Table A5 in S1 Supporting Information, $x_{a_{1}a_{2}i}=x_{d_{1}d_{2}i}$ and $x_{a_{1}d_{2}i}=-x_{d_{1}a_{2}i}=\frac{1}{2}\left(x_{a_{1}i}-x_{a_{2}i}\right)$, model (1) can be reduced to

$$Z_{1i}=\mu_{z_{1}}+x_{a_{1}i}a_{1}{}^{*}+x_{a_{2}i}a_{2}{}^{*}+x_{{\overset{↔}{i}}_{12}i}{\overset{↔}{i}}_{12}+e_{1i},$$ (2)

where $\mu_{z_{1}}=2\mu +d_{1}+d_{2}$, $a_{1}{}^{*}=a_{1}+\frac{1}{2}\left(i_{a_{1}d_{2}}-i_{d_{1}a_{2}}\right)$, $a_{2}{}^{*}=a_{2}+\frac{1}{2}\left(i_{d_{1}a_{2}}-i_{a_{1}d_{2}}\right)$, ${\overset{↔}{i}}_{12}=i_{a_{1}a_{2}}+i_{d_{1}d_{2}}$, and $x_{{\overset{↔}{i}}_{12}i}=x_{a_{1}a_{2}i}=x_{d_{1}d_{2}i}$. If the quantitative trait was controlled by *q* QTL, model (2) should be extended to

$$Z_{1i}=\mu_{z_{1}}+\overset{q}{\underset{k=1}{\sum }}x_{a_{k}{}^{*}i}a_{k}{}^{*}+\overset{q-1}{\underset{k=1}{\sum }}\overset{q}{\underset{l=k+1}{\sum }}x_{{\overset{↔}{i}}_{{}_{kl}}i}{\overset{↔}{i}}_{kl}+e_{1i},$$ (3)

where the model mean $\mu_{z_{1}}=2\mu +\sum_{k=1}^{q}d_{k}$; $a_{k}{}^{*}=a_{k}+\frac{1}{2}\sum_{l\neq k}^{q}\left(i_{a_{k}d_{l}}-i_{d_{k}a_{l}}\right)$ is the augmented additive effect of QTL *k*; ${\overset{↔}{i}}_{kl}=i_{a_{k}a_{l}}+i_{d_{k}d_{l}}$ is the augmented epistatic effect between QTL *k* and *l*. Coefficients $x_{a_{k}{}^{*}i}$ and $x_{{\overset{↔}{i}}_{{}_{kl}}i}$ are determined by genotypes of the *k*th and *l*th QTL for the *i*th RIL line, as shown in Table 1.

**Table 1:** Coefficients of genetic parameters for the RIL based aTTC Z_1i_, Z_2i_ and Z_4i_ data under the F_∞_metric model.

				F_∞_ metric model				
Genotype of Marker		*Z*_1*i*_			*Z*_2*i*_		*Z*_4*i*_	
	$x_{a_{k}{}^{*}}$	$x_{a_{l}{}^{*}}$	$x_{{\overset{↔}{i}}_{{}_{kl}}}$	$u_{d_{k}{}^{*}}$	$u_{d_{l}{}^{*}}$	$u_{{\tilde{i}}_{{}_{kl}}}$	$w_{{\vec{i}}_{{}_{kl}}}$	$w_{{\overset{\leftarrow }{i}}_{{}_{kl}}}$
*M*_*k*_*M*_*k*_*M*_*l*_*M*_*l*_	1	1	1	-1	-1	0	-1	0
*M*_*k*_*M*_*k*_*m*_*l*_*m*_*l*_	1	-1	0	-1	1	1	1	1
*m*_*k*_*m*_*k*_*M*_*l*_*M*_*l*_	-1	1	0	1	-1	1	1	-1
*m*_*k*_*m*_*k*_*m*_*l*_*m*_*l*_	-1	-1	1	1	1	0	-1	0

Similarly, the phenotypic value of Z_2i_ can be described as

$$Z_{2i}=a_{1}+u_{d_{1}i}d_{1}+a_{2}+u_{d_{2}i}d_{2}+u_{a_{1}a_{2}i}i_{a_{1}a_{2}}+u_{a_{1}d_{2}i}i_{a_{1}d_{2}}+u_{d_{1}a_{2}i}i_{d_{1}a_{2}}+u_{d_{1}d_{2}i}i_{d_{1}d_{2}}+e_{2i},$$ (4)

where $u_{d_{1}i}$, $u_{d_{2}i}$, $u_{a_{1}a_{2}i}$, $u_{a_{1}d_{2}i}$, $u_{d_{1}a_{2}i}$, and $u_{d_{1}d_{2}i}$ are determined by the genotype of the *i*th RIL line (Table A6 in S1 Supporting Information), *e*_2*i*_ is the residual error with an $N(0,\sigma_{2}^{2})$ distribution. According to Table A6 in S1 Supporting Information, $u_{a_{1}d_{2}i}=u_{d_{1}a_{2}i}$ and $u_{a_{1}a_{2}i}=-u_{d_{1}d_{2}i}=-\frac{1}{2}\left(u_{d_{1}i}+u_{d_{2}i}\right)$, model (4) can be reduced to

$$Z_{2i}=\mu_{z_{2}}+u_{d_{1}i}d_{1}{}^{*}+u_{d_{2}i}d_{2}{}^{*}+u_{{\tilde{i}}_{12}i}{\tilde{i}}_{12}+e_{2i},$$ (5)

where $\mu_{z_{2}}=a_{1}+a_{2}$, $d_{1}{}^{*}=d_{1}-\frac{1}{2}\left(i_{a_{1}a_{2}}-i_{d_{1}d_{2}}\right)$, $d_{2}{}^{*}=d_{2}-\frac{1}{2}\left(i_{a_{1}a_{2}}-i_{d_{1}d_{2}}\right)$, ${\tilde{i}}_{12}=i_{a_{1}d_{2}}+i_{d_{1}a_{2}}$, and $u_{{\tilde{i}}_{12}i}=u_{a_{1}d_{2}i}=u_{d_{1}a_{2}i}$. If the quantitative trait controlled by *q* QTL, model (5) should be extended to

$$Z_{2i}=\mu_{z_{2}}+\overset{q}{\underset{k=1}{\sum }}u_{d_{k}{}^{*}i}d_{k}{}^{*}+\overset{q-1}{\underset{k=1}{\sum }}\overset{q}{\underset{l=k+1}{\sum }}u_{{\tilde{i}}_{{}_{kl}}i}{\tilde{i}}_{kl}+e_{2i},$$ (6)

where the model mean $\mu_{z_{2}}=\sum_{k=1}^{q}a_{k}$; $d_{k}{}^{*}=d_{k}-\frac{1}{2}\sum_{l\neq k}^{q}\left(i_{a_{k}a_{l}}-i_{d_{k}d_{l}}\right)$ is the augmented dominance effect of QTL *k*, ${\tilde{i}}_{kl}=i_{a_{k}d_{l}}+i_{d_{k}a_{l}}$ is the augmented epistatic effect between QTL *k* and *l*. Coefficients $u_{d_{k}{}^{*}i}$ and $u_{{\tilde{i}}_{{}_{kl}}i}$ are determined by genotypes of the *k*th and *l*th QTL for the *i*th RIL line, as shown in Table 1.

Similarly, the phenotypic value of Z_3i_ can be described as

$$\begin{array}{l} Z_{3i}=ri_{a_{1}a_{2}}+v_{a_{1}d_{2}i}i_{a_{1}d_{2}}+v_{d_{1}a_{2}i}i_{d_{1}a_{2}}+v_{d_{1}d_{2}i}i_{d_{1}d_{2}}+e_{3i}\\ =\mu_{z3}+v_{a_{1}d_{2}i}i_{a_{1}d_{2}}+v_{d_{1}a_{2}i}i_{d_{1}a_{2}}+v_{d_{1}d_{2}i}i_{d_{1}d_{2}}+e_{3i}, \end{array}$$ (7)

where $\mu_{z3}=ri_{a_{1}a_{2}}$, *r* is the recombination fraction between two QTL; dummy variables $v_{a_{1}d_{2}i}$, $v_{d_{1}a_{2}i}$, and $v_{d_{1}d_{2}i}$ are determined by the genotype of the *i*th RIL line (Table A7 in S1 Supporting Information). *e*_3*i*_ is the residual error with an $N(0,\sigma_{3}^{2})$ distribution. Genetic effects $i_{a_{1}d_{2}},i_{d_{1}a_{2}},i_{d_{1}d_{2}}$ can be estimated directly.

In the same way, the phenotypic value of Z_4i_ can be described as

$$Z_{4i}=\mu +d_{1}+d_{2}+w_{a_{1}a_{2}i}i_{a_{1}a_{2}}+w_{a_{1}d_{2}i}i_{a_{1}d_{2}}+w_{d_{1}a_{2}i}i_{d_{1}a_{2}}+w_{d_{1}d_{2}i}i_{d_{1}d_{2}}+e_{4i},$$ (8)

where $w_{a_{1}a_{2}i}$, $w_{a_{1}d_{2}i}$, $w_{d_{1}a_{2}i}$, and $w_{d_{1}d_{2}i}$ are determined by the genotype of the *i*th RIL line (Table A8 in S1 Supporting Information), *e*_4*i*_ is the residual error with an $N(0,\sigma_{4}^{2})$ distribution. According to Table A8 in S1 Supporting Information, there are $w_{a_{1}d_{2}i}=-w_{d_{1}a_{2}i}$ and $w_{a_{1}a_{2}i}=1-w_{d_{1}d_{2}i}$. Therefore, model (8) can be reduced to

$$Z_{4i}=\mu_{z_{4}}+w_{{\vec{i}}_{{}_{12}}i}{\vec{i}}_{12}+w_{{\overset{\leftarrow }{i}}_{{}_{12}}i}{\overset{\leftarrow }{i}}_{12}+e_{4i},$$ (9)

where $\mu_{z_{4}}=\mu +d_{1}+d_{2}+i_{d_{1}d_{2}}$, ${\vec{i}}_{12}=i_{a_{1}a_{2}}-i_{d_{1}d_{2}}$, ${\overset{\leftarrow }{i}}_{12}=i_{a_{1}d_{2}}-i_{d_{1}a_{2}}$, and $w_{{\vec{i}}_{{}_{12}}i}=w_{a_{1}a_{2}i}$, $w_{{\overset{\leftarrow }{i}}_{{}_{12}}i}=w_{a_{1}d_{2}i}$.

If the quantitative trait was controlled by *q* QTL, model (9) can be extended to

$$Z_{4i}=\mu_{z_{4}}+\overset{q}{\underset{k\neq q}{\sum }}w_{{\vec{i}}_{{}_{kl}}i}{\vec{i}}_{{}_{kl}}+\overset{q}{\underset{k\neq q}{\sum }}w_{{\overset{\leftarrow }{i}}_{{}_{kl}}i}{\overset{\leftarrow }{i}}_{{}_{kl}}+e_{4i},$$ (10)

where the model mean $\mu_{z_{4}}=\mu +\sum_{k=1}^{q}d_{k}+\sum_{l\neq k}^{q}i_{d_{k}d_{l}}$, ${\vec{i}}_{kl}=i_{a_{k}a_{l}}-i_{d_{k}d_{l}}$, ${\overset{\leftarrow }{i}}_{kl}=i_{a_{k}d_{l}}-i_{d_{k}a_{l}}$ is the augmented epistatic effect between QTL *k* and *l*. Coefficients $w_{{\vec{i}}_{{}_{kl}}i}$ and $w_{{\overset{\leftarrow }{i}}_{{}_{kl}}i}$ are determined by genotypes of the *k*th and *l*th QTL for the *i*th RIL line, as shown in Table 1.

In the same way, the phenotypic value of Z_5i_ can be described as

$$Z_{5i}=3\mu +s_{a_{1}i}a_{1}+\frac{3}{2}d_{1}+s_{a_{2}i}a_{2}+\frac{3}{2}d_{2}+s_{a_{1}a_{2}i}i_{a_{1}a_{2}}+s_{a_{1}d_{2}i}i_{a_{1}d_{2}}+s_{d_{1}a_{2}i}i_{d_{1}a_{2}}+s_{d_{1}d_{2}i}i_{d_{1}d_{2}}+e_{5i},$$ (11)

where $s_{a_{1}i}$, $s_{a_{2}i}$, $s_{a_{1}a_{2}i}$, $s_{a_{1}d_{2}i}$, $s_{d_{1}a_{2}i}$, and $s_{d_{1}d_{2}i}$ are determined by the genotype of the *i*th RIL line (Table A9 in S1 Supporting Information), *e*_5*i*_ is the residual error with an $N(0,\sigma_{5}^{2})$ distribution. According to Table A9 in S1 Supporting Information, model (11) can be reduced to

$$Z_{5i}=\mu_{z_{5}}+s_{a_{1}i}a_{1}+s_{a_{2}i}a_{2}+s_{a_{1}a_{2}i}i_{a_{1}a_{2}}+s_{a_{1}d_{2}i}i_{a_{1}d_{2}}+s_{d_{1}a_{2}i}i_{d_{1}a_{2}}+s_{d_{1}d_{2}i}i_{d_{1}d_{2}}+e_{5i},$$ (12)

where the model mean $\mu_{z_{5}}=3(\mu +\frac{1}{2}d_{1}+\frac{1}{2}d_{2})$. Genetic effects $a_{1},a_{2},i_{a_{1}a_{2}},i_{a_{1}d_{2}},i_{d_{1}a_{2}},i_{d_{1}d_{2}}$ can be estimated directly.

In the same way, the phenotypic value of Z_6i_ can be described as

$$Z_{6i}=\mu +d_{1}+d_{2}+t_{a_{1}a_{2}i}i_{a_{1}a_{2}}+t_{a_{1}d_{2}i}i_{a_{1}d_{2}}+t_{d_{1}a_{2}i}i_{d_{1}a_{2}}+t_{d_{1}d_{2}i}i_{d_{1}d_{2}}+e_{6i},$$ (13)

where $t_{a_{1}a_{2}i}$, $t_{a_{1}d_{2}i}$, $t_{d_{1}a_{2}i}$, and $t_{d_{1}d_{2}i}$ are determined by the genotype of the *i*th RIL line (Table A10 in S1 Supporting Information), *e*_6*i*_ is the residual error with an $N(0,\sigma_{6}^{2})$ distribution. According to Table A10 in S1 Supporting Information, model (13) can be reduced to

$$Z_{6i}=\mu_{z6}+t_{a_{1}a_{2}i}i_{a_{1}a_{2}}+t_{a_{1}d_{2}i}i_{a_{1}d_{2}}+t_{d_{1}a_{2}i}i_{d_{1}a_{2}}+t_{d_{1}d_{2}i}i_{d_{1}d_{2}}+e_{6i},$$ (14)

where the model mean *μ*_*z*6_ = *μ* + *d*_1_ + *d*_2_. Genetic effects $i_{a_{1}a_{2}},i_{a_{1}d_{2}},i_{d_{1}a_{2}},i_{d_{1}d_{2}}$ can be calculated directly.

Model parameter components for Z_1i_, Z_2i_, Z_3i_, Z_4i_, Z_5i_ and Z_6i_ in the RIL-based aTTC design under both the F_∞_ were listed in Table 2.

**Table 2:** Model parameter components for Z_1i_, Z_2i_, Z_3i_, Z_4i_, Z_5i_ and Z_6i_ in the RIL-based aTTC design under both the F_∞_ model.

Data	F_∞_ metric models		
	Model mean	Main effect	Epistatic effect
*Z*_1*i*_	$\mu_{z_{1}}=2\mu +\sum_{k=1}^{q}d_{k}$	$a_{k}{}^{*}=a_{k}+\frac{1}{2}\sum_{l\neq k}^{q}\left(i_{a_{k}d_{l}}-i_{d_{k}a_{l}}\right)$	${\overset{↔}{i}}_{kl}=i_{a_{k}a_{l}}+i_{d_{k}d_{l}}$
*Z*_2*i*_	$\mu_{z_{2}}=\sum_{k=1}^{q}a_{k}$	$d_{k}{}^{*}=d_{k}-\frac{1}{2}\sum_{l\neq k}^{q}\left(i_{a_{k}a_{l}}-i_{d_{k}d_{l}}\right)$	${\tilde{i}}_{kl}=i_{a_{k}d_{l}}+i_{d_{k}a_{l}}$
*Z*_3*i*_	$\mu_{z3}=ri_{a_{1}a_{2}}$	-	$i_{a_{1}d_{2}},i_{d_{1}a_{2}},i_{d_{1}d_{2}}$
*Z*_4*i*_	$\mu_{z_{4}}=\mu +\sum_{l\neq k}^{q}\left(d_{k}+d_{l}+i_{d_{k}d_{l}}\right)$	-	${\vec{i}}_{kl}=i_{a_{k}a_{l}}-i_{d_{k}d_{l}}$ ${\overset{\leftarrow }{i}}_{kl}=i_{a_{k}d_{l}}-i_{d_{k}a_{l}}$
*Z*_5*i*_	$\mu_{z_{5}}=3(\mu +\frac{1}{2}d_{1}+\frac{1}{2}d_{2})$	*a*_1_,*a*_2_	$i_{a_{1}a_{2}},i_{a_{1}d_{2}},i_{d_{1}a_{2}},i_{d_{1}d_{2}}$
*Z*_6*i*_	*μ*_*z*6_ = *μ* + *d*_1_ + *d*_2_	-	$i_{a_{1}a_{2}},i_{a_{1}d_{2}},i_{d_{1}a_{2}},i_{d_{1}d_{2}}$

### Genetic models for mapping heterotic QTL in F_2_-based aTTC design

Genetic models for mapping heterotic QTL in the F_2_-based aTTC design under both F_∞_ and F_2_ metric can be found in S4 Supporting Information.

### Parameter estimation

For a continuously distributed trait, the observed phenotypic value *y*_*i*_ of individual *i* can be described by the linear regression model

$$y_{i}=\mu +\overset{q}{\underset{k=1}{\sum }}x_{ki}a_{k}+\overset{q-1}{\underset{k=1}{\sum }}\overset{q}{\underset{l=k+1}{\sum }}x_{kli}i_{kl}+e_{i}=\mu +\overset{p}{\underset{j=1}{\sum }}x_{ji}\beta_{j}+e_{i},\;i=1,2,\cdots ,n,$$ (15)

where *q* is the number of markers, *μ* is the overall mean, *x*_*ki*_ denotes the genotype of marker *k* for individual *i* and is defined as −1 or 1 for the two genotypes in the mapping population, and *x*_*kli*_ represents the epistatic genotype between the *k*th and *l*th QTL of individual *i*, and is obtained as the element-wise product of *x*_*kj*_ and *x*_*lj*_. In addition, *a*_*k*_ and *i*_*kl*_ are the corresponding augmented main and epistatic effects, respectively. $p=q+\frac{1}{2}q(q-1)$ is the total number of genetic effects and *x*_*ji*_ and *β*_*j*_ are the corresponding genotypes and coefficients, including the main and epistatic effects. *e*_*i*_ is the residual error assumed to follow an *N*(0,*σ*^2^) distribution.

Model (15) can be written as

$$y=\mu +X_{G}\beta_{G}+X_{GG^{'}}\beta_{GG^{'}}+e,$$ (16)

where vectors *β*_*G*_ and $\beta_{GG^{'}}$ represent the augmented main and epistatic effects of all markers, respectively. *X*_*G*_ and $X_{GG^{'}}$ are corresponding design matrices of different effects and *e* is the residual error that follows an *N*(0,*σ*^2^) distribution. Defining $\beta ={\left[\beta_{G}^{T},\beta_{GG^{'}}^{T}\right]}^{T}$ and $X=[X_{G},X_{GG^{'}}]$, model (16) can be written in a more compact form

y=μ+Xβ+e. (17)

Due to the physical linkage or epistatic interactions among multiple QTL, it is rational when taking a large number of loci into consideration simultaneously. However, the total number of genetic effects p is very large because we set each marker as a QTL initially. Typically, we have *p* >> *n*. To handle such an oversaturated model, we employed a fast empirical Bayesian LASSO (EBLASSO) algorithm. Simulation studies demonstrate that the EBLASSO method can sharply reduce the computational burden by shrinking small effects into zero, and can detect more true QTL effects without increasing the false-positive rate. Details of the EBLASSO algorithm can be seen by reference to the work of Cai et al. [41]. At last, all remaining markers with $t_{j}=\left|{\hat{\beta }}_{j}\right|/{\hat{\sigma }}_{j}>2.0$ are picked up, where ${\hat{\sigma }}_{j}$ is the standard deviation estimation for normal prior ${\hat{\beta }}_{j}∼N(0,\sigma_{{}_{j}}^{2})$. The epistatic model is then established that only includes effects that pass the first round of selection. We also perform a usual likelihood ratio test on the model to obtain significant QTL and epistatic interactions. With transformation combinations (Z1, Z2, and Z4), following the above steps, in the first round, the argument effects *a*_k_^*^, ${\overset{↔}{i}}_{kl}$, *d*_k_^*^, ${\tilde{i}}_{kl}$, ${\vec{i}}_{kl}$, and ${\overset{\leftarrow }{i}}_{kl}$ can be obtained; in the second round, main and epistatic effects of each QTL and interaction can be calculated according to equation transformations $a_{k}{}^{*}=a_{k}+\frac{1}{2}\sum_{l\neq k}^{q}\left(i_{a_{k}}{}_{d_{l}}-i_{d_{k}}{}_{a_{l}}\right)$, ${\overset{↔}{i}}_{kl}=i_{a_{k}}{}_{a_{l}}+i_{d_{k}}{}_{d_{l}}$, $d_{k}{}^{*}=d_{k}-\frac{1}{2}\sum_{l\neq k}^{q}\left(i_{a_{k}}{}_{a_{l}}-i_{d_{k}}{}_{d_{l}}\right)$, ${\tilde{i}}_{kl}=i_{a_{k}}{}_{d_{l}}+i_{d_{k}}{}_{a_{l}}$, ${\vec{i}}_{kl}=i_{a_{k}}{}_{a_{l}}-i_{d_{k}}{}_{d_{l}}$, and ${\overset{\leftarrow }{i}}_{kl}=i_{a_{k}}{}_{d_{l}}-i_{d_{k}}{}_{a_{l}}$.

Take any two significant QTL in a model as an example to explain how we obtain genetic effects. After performing QTL mapping, genetic parameters ${\vec{i}}_{12}$ and ${\overset{\leftarrow }{i}}_{12}$ in *Z*_4*i*_, ${\overset{↔}{i}}_{12}$ in *Z*_1*i*_, and ${\tilde{i}}_{12}$ in *Z*_2*i*_ can be obtained. For ${\vec{i}}_{12}=i_{a_{1}a_{2}}-i_{d_{1}d_{2}}$ and ${\overset{↔}{i}}_{12}=i_{a_{1}a_{2}}+i_{d_{1}d_{2}}$, so $i_{a_{1}a_{2}}=({\vec{i}}_{12}+{\overset{↔}{i}}_{12})/2$, $i_{d_{1}d_{2}}=({\overset{↔}{i}}_{12}-{\vec{i}}_{12})/2$, while ${\overset{\leftarrow }{i}}_{12}=i_{a_{1}d_{2}}-i_{d_{1}a_{2}}$ and ${\tilde{i}}_{12}=i_{a_{1}d_{2}}+i_{d_{1}a_{2}}$; we then get $i_{a_{1}d_{2}}=({\overset{\leftarrow }{i}}_{12}+{\tilde{i}}_{12})/2$, $i_{d_{1}a_{2}}=({\tilde{i}}_{12}-{\overset{\leftarrow }{i}}_{12})/2$. Meanwhile, *a*_1_^*^, *a*_2_^*^, *d*_1_^*^, and *d*_2_^*^ are obtained after performing QTL mapping in *Z*_1*i*_ and *Z*_2*i*_ because $a_{1}{}^{*}=a_{1}+\frac{1}{2}(i_{a_{1}d_{2}}-i_{d_{1}a_{2}})$, $a_{2}{}^{*}=a_{2}+\frac{1}{2}(i_{d_{1}a_{2}}-i_{a_{1}d_{2}})$, $d_{1}{}^{*}=d_{1}+\frac{1}{2}(i_{a_{1}a_{2}}-i_{d_{1}d_{2}})$, and $d_{2}{}^{*}=d_{2}+\frac{1}{2}(i_{a_{1}a_{2}}-i_{d_{1}d_{2}})$, with the estimation value of *d*_1_^*^, *d*_2_^*^, and above $i_{a_{1}a_{2}}$, $i_{d_{1}d_{2}}$, $i_{a_{1}d_{2}}$, and $i_{d_{1}a_{2}}$ values, main effects can be calculated by $d_{1}=d_{1}^{*}-\frac{1}{2}(i_{a_{1}a_{2}}-i_{d_{1}d_{2}})$, $d_{2}=d_{2}^{*}-\frac{1}{2}(i_{a_{1}a_{2}}-i_{d_{1}d_{2}})$, $a_{1}=a_{1}^{*}-\frac{1}{2}(i_{a_{1}d_{2}}-i_{d_{1}a_{2}})$, and $a_{2}=a_{2}^{*}-\frac{1}{2}(i_{d_{1}a_{2}}-i_{a_{1}d_{2}})$. All main and epistatic effects were dissected by the integration of augmented effects in Z_1_, Z_2_, and Z_4_.

Similarity, with transformation combination (Z_1_, Z_2_, and Z_5_) and (Z_1_, Z_2_, and Z_6_), we can also get the genetic effect of each QTL or interaction by QTL mapping under the aTTC design, respectively. More details are listed in S1 Supporting Information.

### Simulation study

We took all the possible types of epistatic interaction patterns into consideration. The simulated genome, covered by 100 evenly spaced markers with a marker interval of 5 cM, was 495 cM in total length and comprised four chromosomes. For data sets Z_1_, Z_2_, or Z_4_, six QTL positions were preset, of which three positions (QTL_1_, QTL_2_, and QTL_4_) had main effects. Pairwise interactions were set between positions with main effects (QTL_1_ and QTL_2_), with and without main effects (QTL_3_ and QTL_4_), and without main effects (QTL_5_ and QTL_6_), respectively. The assumed QTL positions, parameters, and augmented effects (including main and epistatic effects) are listed in Table 3 and Tables A-B in S11 Supporting Information. The sample size (*n*) was set at three levels: 800, 400, and 200. The broad heritability (*h*) was also set at three levels: 0.8, 0.5, and 0.2, separately representing high, middle, and low heritabilities. The replication number of offspring (*m*) was set at two levels: 5 and 10. In three transformations, Z_1_, Z_2_, and Z_4_, each treatment was replicated 100 times. Simulation study also be conducted for Z_5_ and Z_6_. The results of Z_5_ and Z_6_ were listed in Tables C and D in S11 Supporting Information.

**Table 3:** QTL mapping results for Z_4_ in RIL-based aTTC design under the F_∞_ metric model in simulation study.

n	h	m				QTL_1_×QTL_2_								QTL_3_×QTL_4_								QTL_5_×QTL_6_				
			I_1_^a^	pos^c^	pos	p^d^	I_2_^b^	pos	pos	p	I_1_	pos	pos	p	I_2_	pos	pos	p	I_2_	pos	pos	p	I_1_	pos	pos	p
			3.2	20	36		2.4	20	36		-3.6	45	70		2.2	45	70		3	80	95		3.4	80	95	
800	0.8	10	3.204	20	36	1	2.399	20	36	1	-3.596	45	70	1	2.194	45	70	1	2.994	80	95	1	3.385	80	95	1
			(0.062)				(0.03)				(0.062)				(0.035)				(0.03)				(0.062)			
		5	3.191	20	36	1	2.4	20	36	1	-3.594	45	70	1	2.192	45	70	1	2.989	80	95	1	3.38	80	95	1
			(0.075)				(0.045)				(0.085)				(0.05)				(0.048)				(0.093)			
	0.5	10	3.175	20	36	1	2.37	20	36	1	-3.595	45	70	1	2.178	45	70	1	2.989	80	95	1	3.384	80	95	1
			(0.118)				(0.069)				(0.109)				(0.062)				(0.065)				(0.109)			
		5	3.17	20	36	1	2.342	20	36	1	-3.538	45	70	1	2.178	45	70	1	2.985	80	95	1	3.371	80	95	1
			(0.195)				(0.098)				(0.168)				(0.093)				(0.085)				(0.168)			
	0.2	10	3.146	20	36	1	2.333	20	36	1	-3.531	45	70	1	2.141	45	70	1	2.921	80	95	1	3.336	80	95	1
			(0.233)				(0.127)				(0.265)				(0.141)				(0.134)				(0.232)			
		5	3.171	20	36	1	2.278	20	36	0.99	-3.566	45	70	1	2.055	45	70	0.99	2.923	80	95	1	3.338	80	95	1
			(0.354)				(0.192)				(0.417)				(0.255)				(0.193)				(0.342)			
400	0.8	10	3.183	20	36	1	2.385	20	36	1	-3.59	45	70	1	2.192	45	70	1	2.989	80	95	1	3.381	80	95	1
			(0.081)				(0.045)				(0.089)				(0.048)				(0.046)				(0.088)			
		5	3.19	20	36	1	2.374	20	36	1	-3.569	45	70	1	2.176	45	70	1	2.999	80	95	1	3.399	80	95	1
			(0.141)				(0.061)				(0.138)				(0.065)				(0.069)				(0.130)			
	0.5	10	3.16	20	36	1	2.381	20	36	1	-3.58	45	70	1	2.15	45	70	1	2.981	80	95	1	3.345	80	95	1
			(0.156)				(0.092)				(0.158)				(0.085)				(0.099)				(0.173)			
		5	3.154	20	36	1	2.313	20	36	1	-3.52	45	70	1	2.097	45	70	1	2.943	80	95	1	3.344	80	95	1
			(-0.263)				(0.147)				(0.245)				(0.141)				(0.147)				(0.291)			
	0.2	10	3.118	20	36	1	2.283	20	36	1	-3.478	45	70	1	2.066	45	70	0.96	2.904	80	95	0.98	3.316	80	95	1
			(0.351)				(0.337)				(0.345)				(0.277)				(0.197)				(0.337)			
		5	2.899	20	36	0.99	2.247	20	36	0.89	-3.477	45	70	1	1.985	45	70	0.75	2.812	80	95	0.99	2.699	80	95	0.99
			(0.889)				(0.26)				(0.558)				(0.45)				(0.362)				(1.792)			
200	0.8	10	3.18	20	36	1	2.386	20	36	1	-3.592	45	70	1	2.192	45	70	1	2.993	80	95	1	3.389	80	95	1
			(0.118)				(0.069)				(0.126)				(0.065)				(0.065)				(0.139)			
		5	3.192	20	36	1	2.342	20	36	1	-3.56	45	70	1	2.163	45	70	1	2.947	80	95	1	3.346	80	95	1
			(0.166)				(0.095)				(0.181)				(0.097)				(0.098)				(0.174)			
	0.5	10	3.087	20	36	1	2.317	20	36	1	-3.541	45	70	1	2.12	45	70	1	2.943	80	95	1	3.321	80	95	1
			(0.253)				(0.147)				(0.224)				(0.13)				(0.147)				(0.268)			
		5	3.142	20	36	1	2.244	20	36	0.99	-3.552	45	70	1	1.862	45	70	0.97	2.94	80	95	1	3.308	80	95	1
			(0.354)				(0.36)				(0.331)				(0.628)				(0.186)				(0.349)			
	0.2	10	3.068	20	36	0.97	2.329	20	36	0.76	-3.31	45	70	0.93	2.05	45	70	0.64	2.936	80	95	0.97	3.15	80	95	0.97
			(0.47)				(0.257)				(0.859)				(0.326)				(0.263)				(0.829)			
		5	3.591	20	36	0.61	2.834	20	36	0.24	-3.593	45	70	0.61	2.552	45	70	0.25	3.044	80	95	0.49	3.543	80	95	0.59
			(0.612)				(0.494)				(0.624)				(0.332)				(0.359)				(0.527)			

Epistatic effects estimated in Z_4_ were multiplied by 2; n: sample size; h: broad heritability; m: the replication number of offspring.

^a^I_1:_ *i*_*aa*_—*i*_*dd*_.

^b^I_2_: *i*_*ad*_—*i*_*da*_.

^c^pos: position.

^d^p: power.

#### QTL mapping in simulation study

All independent variable $p=100+\frac{1}{2}\times 100\times (100-1)=5050$ for Z_1_, Z_2_, Z_4_ in simulation study were simultaneously included in one genetic model, which was much larger than the sample size. Data sets were implemented in R (version 3.0) with the EBLASSO package obtained from Cai et al. [41]. Hyper-parameters *a* and *b* were obtained by three-fold cross-validation (by default) in each individual model; after 100 replications, hyper-parameters with minimum predicted errors were fixed to estimate parameters. The time was approximately 5 minutes in each transformation in a stand-alone personal computer (Intel Pentium CPU 2.9 GHz; memory 4 GB); therefore, the EBLASSO algorithm presented high efficiency and saved time.

### Real data analysis

Considering the unbiased estimate of coefficients and the excellent detection power in the simulation study, we further applied the proposed approach to a real mapping population and presented a comparison to previous mapping results.

#### Populations

Two elite rice hybrids, one inter-subspecific between 9024 (*indica*) and LH422 (*japonica*) and one intra-subspecific between Zhenshan97 (*indica*) and Minghui63 (*indica*), were analyzed, and details were documented in our previous study [24]. For convenience, we designated the two hybrids as *IJ* and *II* hybrids, respectively. The RIL were derived from the cross of a random sample of F_2_ individuals to their parental lines (194 F_7_ lines for the *IJ* hybrid and 222 F_12_ lines for the *II* hybrid, respectively).

#### Genetic linkage maps

For the *II* hybrid, the linkage map comprised 221 marker loci and covered 1796 cM in total [42]. For the *IJ* hybrid, Xiao et al. [8] constructed a linkage map of the recombinant population in which a subset of 141 polymorphic restriction fragment length polymorphism markers was used.

#### Phenotypic traits

Nine quantitative traits, including heading date (HD, in days), plant height (PH, in centimeters), tillers per plant, panicle length (PL, in centimeters), filled grains per panicle (FGPP), percentage of seed set, grain density (GD, in grain numbers per centimeter of panicle length), 1000-grain weight (KGW, in grams), and grain yield (YD, in tons/hectare) were investigated in RIL, Z_1_, Z_2_, and Z_4_ respectively. All the materials described above were laid out in a field in a randomized complete block design with two replications (plots) for phenotypic evaluation.

#### QTL mapping in real data analysis

Data sets Z_1_, Z_2_ and Z_4_ were implemented in R (version 3.0) with the EBLASSO package obtained from Cai et al. [41] for QTL mapping.

## Results

### Simulation study results

#### Augmented effects in simulation study

As shown in Table 3 and Tables A-B in S11 Supporting Information, the augmented additive ($a_{1}{}^{*}=a_{1}+\frac{1}{2}\left(i_{a_{1}d_{2}}-i_{d_{1}a_{2}}\right)$) and epistatic effects (${\overset{↔}{i}}_{12}=\left(i_{a_{1}a_{2}}+i_{d_{1}d_{2}}\right)$) in Z_1_, augmented dominance ($d_{1}{}^{*}=d_{1}-\frac{1}{2}\left(i_{a_{1}a_{2}}-i_{d_{1}d_{2}}\right)$) and epistatic effects (${\tilde{i}}_{12}=\left(i_{a_{1}d_{2}}+i_{d_{1}a_{2}}\right)$) in Z_2_, augmented epistatic effects (${\vec{i}}_{12}=i_{a_{1}a_{2}}-i_{d_{1}d_{2}}$, ${\overset{\leftarrow }{i}}_{12}=i_{a_{1}d_{2}}-i_{d_{1}a_{2}}$) in Z_4_ were rightly and unbiased estimated with a high statistical power in preset positions. The ratio of the number of samples, in which the LOD statistic was greater than 2.5, to the total number of replicates represented the empirical power of this simulated QTL or interaction.

**Fig 1:**
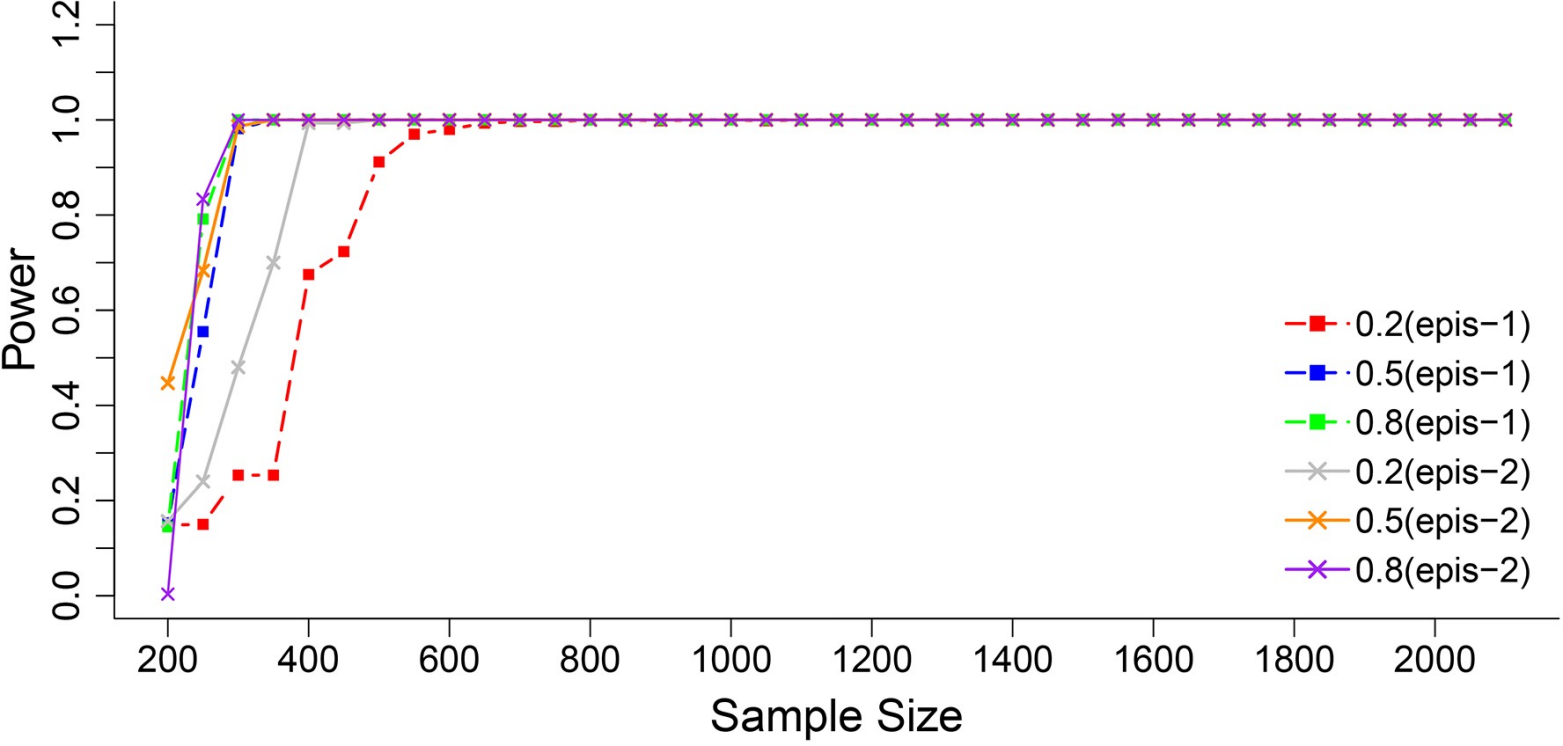
The mean statistic power of augmented epistatic effect interactions in Z_4_. Epis-1 refer the augmented epistatic effect (*i*_*aa*_*—i*_*dd*_) interaction, epis-2 refer the augmented epistatic effect (*i*_*ad*_*—i*_*da*_) interaction.

In Z_1_ transformation (Table A in S11 Supporting Information, S1 Fig), when the sample size was 800 or 400, almost all augmented additive and epistatic effects were detected, except for the detection power of digenic interactions in 400 samples, 0.2 heritability, and 5 replications. This indicated that smaller heritability or less individual replication had little influence on the detection of QTL in a relatively large sample size. When the sample size was reduced to 200, all the preset QTL were detected successfully with a heritability of 0.8 and 0.5; however, detection power decreased sharply to the level of 0.2, which is more true for the preset digenic interactions. When individual replication was 5, the detection power of the augment additive effect of QTL_3_ was 0.73, whereas it was 0.985 when the individual replication was 10, and the detection power of augmented epistatic effects dropped to 0.59 for interaction QTL_5_ × QTL_6_. Similar results could be found in Z_2_ (Table B in S11 Supporting Information, S2 Fig); all the preset QTL were precisely detected and the QTL effects were estimated in an unbiased manner, even on the level of the smallest sample size (200) or the lowest heritability (0.2). In addition, all the augmented epistatic effects estimated in an unbiased manner in Z_4_ (Table 3, Fig 1). The poor detection power occurred only on a low heritability level (0.2) with sample sizes 400 or 200.

#### Main and epistatic effects in simulation study

Table 4 shown the main and epistatic effects of QTL_1_ and QTL_2_ in the in RIL-based aTTC design using the two-step approach under the F_∞_ metric model. Other pairs of interactions are listed in Tables 5 and 6 for the interaction between QTL_3_ and QTL_4_ and QTL_5_ and QTL_6_, respectively. We can see that the main-effect and epistatic effects of QTL were very close to set value when sample size is big (800) and heritability is high (0.8). all the preset QTL were precisely detected and the QTL effects were estimated in an unbiased manner, except on the level of the smallest sample size (200) and the lowest heritability (0.2) with 5 replications in Z_1_, Z_2_ and Z_4_.

**Table 4:** Dissected main and epistatic effects of QTL_1_ and QTL_2_ in the in RIL-based aTTC design using the two-step approach under the F_∞_ metric model in simulation study.

N	h	m	statistics	QTL_1_		QTL_2_		QTL_1_×QTL_2_			
				*a*_*1*_	*d*_*1*_	*a*_*2*_	*d*_*2*_	*a*_*1*_*a*_*2*_	*a*_*1*_*d*_*2*_	*d*_*1*_*a*_*2*_	*d*_*1*_*d*_*2*_
Parameter values				1.8	3.2	2.8	-1.5	2.7	3.4	1	-0.5
800	0.8	10	mean	1.802	3.204	2.795	-1.501	2.699	3.399	0.999	-0.506
			sd	(0.022)	(0.038)	(0.021)	(0.036)	(0.021)	(0.030)	(0.033)	(0.049)
		5	mean	1.804	3.192	2.794	-1.506	2.698	3.403	1.004	-0.492
			sd	(0.032)	(0.047)	(0.030)	(0.051)	(0.026)	(0.047)	(0.045)	(0.061)
	0.5	10	mean	1.807	3.183	2.789	-1.511	2.686	3.370	1.000	-0.489
			sd	(0.049)	(0.071)	(0.043)	(0.069)	(0.045)	(0.066)	(0.061)	(0.093)
		5	mean	1.825	3.181	2.758	-1.523	2.672	3.356	1.015	-0.498
			sd	(0.058)	(0.124)	(0.064)	(0.120)	(0.068)	(0.095)	(0.097)	(0.155)
	0.2	10	mean	1.830	3.158	2.748	-1.526	2.653	3.350	1.017	-0.493
			sd	(0.084)	(0.154)	(0.089)	(0.154)	(0.092)	(0.131)	(0.141)	(0.177)
		5	mean	1.827	3.142	2.688	-1.492	2.614	3.333	1.067	-0.557
			sd	(0.151)	(0.222)	(0.122)	(0.228)	(0.123)	(0.178)	(0.175)	(0.284)
400	0.8	10	mean	1.808	3.184	2.795	-1.515	2.689	3.388	1.002	-0.494
			sd	(0.035)	(0.052)	(0.030)	(0.051)	(0.031)	(0.042)	(0.044)	(0.064)
		5	mean	1.810	3.187	2.789	-1.506	2.688	3.385	1.011	-0.501
			sd	(0.044)	(0.084)	(0.044)	(0.087)	(0.050)	(0.061)	(0.065)	(0.103)
	0.5	10	mean	1.807	3.156	2.786	-1.525	2.673	3.378	0.998	-0.486
			sd	(0.066)	(0.096)	(0.062)	(0.099)	(0.063)	(0.084)	(0.084)	(0.126)
		5	mean	1.831	3.168	2.744	-1.518	2.660	3.354	1.041	-0.494
			sd	(0.099)	(0.166)	(0.090)	(0.164)	(0.098)	(0.132)	(0.143)	(0.201)
	0.2	10	mean	1.842	3.118	2.698	-1.559	2.625	3.305	1.022	-0.494
			sd	(0.176)	(0.216)	(0.179)	(0.219)	(0.153)	(0.244)	(0.226)	(0.313)
		5	mean	1.946	2.993	2.568	-1.629	2.399	3.206	1.138	-0.471
			sd	(0.251)	(0.513)	(0.257)	(0.491)	(0.461)	(0.311)	(0.375)	(0.687)
200	0.8	10	mean	1.805	3.184	2.785	-1.500	2.688	3.391	1.006	-0.492
			sd	(0.050)	(0.072)	(0.044)	(0.073)	(0.042)	(0.071)	(0.058)	(0.096)
		5	mean	1.816	3.190	2.771	-1.484	2.668	3.360	1.018	-0.525
			sd	(0.059)	(0.113)	(0.069)	(0.099)	(0.059)	0.086	0.083	0.133
	0.5	10	mean	1.834	3.137	2.749	-1.557	2.635	3.355	1.038	-0.451
			sd	(0.098)	(0.153)	(0.092)	(0.151)	(0.088)	0.141	0.135	0.201
		5	mean	1.862	3.129	2.684	-1.510	2.611	3.288	1.054	-0.531
			sd	(0.194)	(0.210)	(0.208)	(0.219)	(0.146)	0.244	0.231	0.329
	0.2	10	mean	1.991	3.057	2.498	-1.588	2.474	3.156	1.239	-0.502
			sd	(0.312)	(0.393)	(0.290)	(0.397)	(0.398)	0.359	0.376	0.602
		5	mean	2.169	2.626	2.099	-1.983	1.830	2.770	1.423	-0.908
			sd	(0.474)	(0.958)	(0.489)	(0.961)	(0.515)	0.569	0.505	1.330

**Table 5:** Dissected main and epistatic effects of QTL_3_ and QTL_4_ in the in RIL-based aTTC design using the two-step approach under the F_∞_ metric model in simulation study.

N	h	m	statistics	QTL_3_		QTL_4_		QTL_3_×QTL_4_			
				*a*_*3*_	*d*_*3*_	*a*_*4*_	*d*_*4*_	*a*_*3*_*a*_*4*_	*a*_*3*_*d*_*4*_	*d*_*3*_*a*_*4*_	*d*_*3*_*d*_*4*_
Parameter values				0	0	-2	2.1	-0.7	-1.6	-3.8	2.9
800	0.8	10	mean	0.001	0.003	-2.003	2.097	-0.698	-1.605	-3.798	2.898
			sd	(0.022)	(0.038)	(0.020)	(0.042)	(0.022)	(0.035)	(0.029)	(0.048)
		5	mean	0.001	0.002	-2.006	2.100	-0.702	-1.602	-3.794	2.892
			sd	(0.033)	(0.061)	(0.034)	(0.053)	(0.028)	(0.047)	(0.044)	(0.074)
	0.5	10	mean	0.008	0.012	-2.007	2.098	-0.703	-1.606	-3.784	2.892
			sd	(0.044)	(0.073)	(0.047)	(0.075)	(0.038)	(0.062)	(0.066)	(0.086)
		5	mean	0.010	0.021	-2.007	2.126	-0.683	-1.614	-3.792	2.855
			sd	(0.063)	(0.114)	(0.054)	(0.100)	(0.067)	(0.089)	(0.088)	(0.123)
	0.2	10	mean	0.023	0.021	-2.029	2.116	-0.695	-1.620	-3.761	2.836
			sd	(0.090)	(0.158)	(0.093)	(0.150)	(0.096)	(0.136)	(0.153)	(0.206)
		5	mean	0.029	0.002	-2.047	2.111	-0.745	-1.664	-3.708	2.821
			sd	(0.193)	(0.230)	(0.166)	(0.256)	(0.187)	(0.237)	(0.226)	(0.389)
400	0.8	10	mean	0.002	0.006	-2.003	2.104	-0.699	-1.602	-3.794	2.891
			sd	(0.030)	(0.053)	(0.030)	(0.059)	(0.031)	(0.040)	(0.043)	(0.072)
		5	mean	0.008	0.001	-2.008	2.116	-0.698	-1.611	-3.788	2.872
			sd	(0.040)	(0.079)	(0.047)	(0.087)	(0.049)	(0.068)	(0.063)	(0.107)
	0.5	10	mean	0.006	0.008	-2.019	2.101	-0.703	-1.629	-3.778	2.877
			sd	(0.055)	(0.101)	(0.058)	(0.096)	(0.051)	(0.087)	(0.092)	(0.134)
		5	mean	0.025	0.014	-2.047	2.121	-0.687	-1.658	-3.755	2.833
			sd	(0.099)	(0.155)	(0.094)	(0.156)	(0.092)	(0.152)	(0.132)	(0.204)
	0.2	10	mean	0.049	0.032	-2.079	2.164	-0.700	-1.661	-3.664	2.778
			sd	(0.213)	(0.226)	(0.230)	(0.225)	(0.121)	(0.255)	(0.273)	(0.274)
		5	mean	0.244	0.010	-2.333	2.142	-0.727	-1.920	-3.419	2.749
			sd	(0.402)	(0.315)	(0.444)	(0.351)	(0.224)	(0.528)	(0.495)	(0.467)
200	0.8	10	mean	0.002	0.002	-2.004	2.105	-0.697	-1.605	-3.797	2.896
			sd	(0.046)	(0.079)	(0.045)	(0.079)	(0.047)	(0.065)	(0.062)	(0.098)
		5	mean	0.011	0.016	-2.011	2.110	-0.693	-1.615	-3.778	2.867
			sd	(0.065)	(0.116)	(0.064)	(0.095)	(0.066)	(0.096)	(0.089)	(0.137)
	0.5	10	mean	0.031	0.017	-2.037	2.129	-0.692	-1.628	-3.749	2.848
			sd	(0.093)	(0.157)	(0.082)	(0.149)	(0.085)	(0.121)	(0.135)	(0.182)
		5	mean	0.127	0.005	-2.164	2.111	-0.700	-1.751	-3.588	2.852
			sd	(0.307)	(0.202)	(0.313)	(0.198)	(0.131)	(0.336)	(0.352)	(0.262)
	0.2	10	mean	0.332	0.243	-2.373	2.352	-0.588	-2.008	-3.340	2.553
			sd	(0.482)	(0.599)	(0.404)	(0.594)	(0.555)	(0.493)	(0.506)	(0.674)
		5	mean	0.255	0.485	-2.637	2.808	-0.737	-2.363	-3.001	2.351
			sd	(0.825)	(1.049)	(0.614)	(0.960)	(0.909)	(0.497)	(0.559)	(0.955)

**Table 6:** Dissected main and epistatic effects of QTL_5_ and QTL_6_ in the in RIL-based aTTC design using the two-step approach under the F_∞_ metric model in simulation study.

n	h	m	statistics	QTL_5_		QTL_6_		QTL_5_×QTL_6_			
				*a*_*5*_	*d*_*5*_	*a*_*6*_	*d*_*6*_	*a*_*5*_*a*_*6*_	*a*_*5*_*d*_*6*_	*d*_*5*_*a*_*6*_	*d*_*5*_*d*_*6*_
Parameter values				0	0	0	0	2.8	3.9	0.9	-0.6
800	0.8	10	mean	0.001	0.008	0.003	0.006	2.791	3.897	0.903	-0.594
			sd	(0.019)	(0.038)	(0.022)	(0.039)	(0.022)	(0.033)	(0.029)	(0.049)
		5	mean	0.006	0.010	0.003	0.008	2.791	3.894	0.905	-0.588
			sd	(0.029)	(0.047)	(0.035)	(0.058)	(0.030)	(0.041)	(0.047)	(0.075)
	0.5	10	mean	0.001	0.002	0.006	0.009	2.788	3.892	0.903	-0.597
			sd	(0.046)	(0.075)	(0.045)	(0.067)	(0.038)	(0.059)	(0.063)	(0.088)
		5	mean	0.005	0.006	0.000	0.003	2.772	3.888	0.903	-0.600
			sd	(0.055)	(0.102)	(0.063)	(0.102)	(0.057)	(0.083)	(0.086)	(0.134)
	0.2	10	mean	0.019	0.031	0.005	0.021	2.746	3.847	0.926	-0.590
			sd	(0.105)	(0.136)	(0.086)	(0.136)	(0.083)	(0.123)	(0.120)	(0.183)
		5	mean	0.016	0.022	0.011	0.011	2.721	3.841	0.918	-0.618
			sd	(0.122)	(0.221)	(0.129)	(0.212)	(0.109)	(0.201)	(0.183)	(0.290)
400	0.8	10	mean	0.001	0.002	0.005	0.007	2.788	3.897	0.908	-0.593
			sd	(0.030)	(0.058)	(0.030)	(0.057)	(0.033)	(0.045)	(0.047)	(0.071)
		5	mean	0.001	0.002	0.002	0.000	2.789	3.897	0.899	-0.610
			sd	(0.046)	(0.079)	(0.047)	(0.077)	(0.046)	(0.059)	(0.068)	(0.100)
	0.5	10	mean	0.002	0.011	0.005	0.030	2.764	3.869	0.889	-0.581
			sd	(0.067)	(0.115)	(0.059)	(0.102)	(0.059)	(0.097)	(0.095)	(0.141)
		5	mean	0.020	0.016	0.005	0.009	2.736	3.859	0.916	-0.608
			sd	(0.095)	(0.182)	(0.084)	(0.180)	(0.097)	(0.142)	(0.138)	(0.225)
	0.2	10	mean	0.008	0.018	0.044	0.010	2.721	3.833	0.928	-0.595
			sd	(0.142)	(0.217)	(0.158)	(0.234)	(0.117)	(0.185)	(0.155)	(0.269)
		5	mean	0.089	0.316	0.068	0.282	2.343	3.791	0.994	-0.329
			sd	(0.264)	(0.905)	(0.255)	(1.015)	(0.879)	(0.344)	(0.338)	(1.027)
200	0.8	10	mean	0.003	0.005	0.000	0.003	2.787	3.896	0.902	-0.603
			sd	(0.044)	(0.080)	(0.041)	(0.076)	(0.049)	(0.061)	(0.062)	(0.106)
		5	mean	0.018	0.018	0.008	0.029	2.768	3.871	0.924	-0.578
			sd	(0.067)	(0.111)	(0.065)	(0.107)	(0.061)	(0.086)	(0.094)	(0.138)
	0.5	10	mean	0.023	0.011	0.001	0.031	2.740	3.846	0.903	-0.581
			sd	(0.099)	(0.151)	(0.097)	(0.169)	(0.085)	(0.136)	(0.140)	(0.209)
		5	mean	0.010	0.021	0.004	0.027	2.723	3.880	0.939	-0.586
			sd	(0.129)	(0.232)	(0.141)	(0.225)	(0.123)	(0.163)	(0.194)	(0.281)
	0.2	10	mean	0.039	0.132	0.060	0.114	2.535	3.806	0.958	-0.520
			sd	(0.276)	(0.532)	(0.265)	(0.495)	(0.469)	(0.294)	(0.368)	(0.696)
		5	mean	0.311	0.573	0.314	0.517	1.837	3.201	1.439	-0.776
			sd	(0.646)	(0.963)	(0.672)	(1.050)	(0.510)	(0.719)	(0.805)	(1.370)

### Real data analysis results

#### QTL mapping in *II* and *IJ* hybrid

In the *II* hybrid, all $p=221+\frac{1}{2}\times 221\times (221-1)=22321$ were simultaneously included in the genetic model, about 115 times as large as the sample size, while in *IJ* hybrids, $p=141+\frac{1}{2}\times 141\times (141-1)=10011$, which was about 45 times bigger than the sample size. QTL effect-explained 1% phenotypic variation was set as a threshold for declaring the presence of QTL. QTL mapping results for the *II* and *IJ* hybrids are listed in Tables A and B in S12 Supporting Information, respectively.

#### Augmented effects in *II* and *IJ* hybrid

As shown in Table A in S12 Supporting Information, 14 QTLs and 36 digenic interactions were detected in the *II* hybrid, and the explained variation of a single QTL or interaction varied from 1.13% to 7.67%. In the RIL mapping population, 8 QTL (16%) were revealed, of which 2 QTLs (25%) were detected in trait GD with relative small phenotypic variation. In trait YD, a digenic interaction of marker C1016 and C483 explained the maximum (7.49%) phenotypic variation. In Z_1_, 11 QTL (22%) were detected, and one or more QTL were revealed in each trait. The explained variation in Z_1_ varied from 1.21% to 7.67%. The interaction between marker R3166 and RZ667 was also detected in traits FGPP and GD, and explained 7.67% and 7.33% variation, respectively. In Z_2_, 10 QTLs were identified. There was no QTL detected in trait PL. The explained variation in Z_2_ varied from 1.13% to 5.91%. In Z_4_, 11 interactions were found, and at least one QTL or interaction was revealed in each trait. The maximum explained variation was in trait HD (6.05%). Ten interactions were also dissected, and the explained variation of a single interaction varied from 1.69% to 7.09%.

In the *IJ* hybrid, as shown in Table B in S12 Supporting Information, a total of 46 QTL and 75 interactions was detected. Of the detected QTL, the majority was detected in RIL (39.37%) and Z_4_ (41.73%). The explained variation of a single QTL in the RIL ranged from 1.09% to 27.48%. In Z_4_, the detected QTL-associated marker RG333 affected HD in chromosome 8 explained 27.48% of phenotypic variation. It was also found simultaneously in Z_1_ and Z_2_ data sets. Eighteen QTLs in Z_1_ and 9 QTLs in Z_2_ were identified. In Z_1_, QTL-associated marker RG333 influenced HD, accounting for 36.58% of variation, which also explained 8.47% of variation for PH. In Z_4_, the majority of interactions were detected in trait FGPP in which 17 (58.6%) and 10 (41.7%) marker pairs were found in ${\vec{i}}_{12}=i_{a_{1}a_{2}}-i_{d_{1}d_{2}}$ and ${\overset{\leftarrow }{i}}_{12}=i_{a_{1}d_{2}}-i_{d_{1}a_{2}}$.

#### Dissection of main and epistatic effects

Integrated in the QTL mapping result of Tables A and B in S12 Supporting Information, main and epistatic effects were dissected by the proposed approach distributed previous part 3.4. The results of the *II* and *IJ* hybrid are presented in Tables 7 and 8, respectively. For main effect QTL, we dissected the additive and dominance effects, whereas for interactions, additive × additive (*aa*), additive × dominance (*ad*), dominance × additive (*da*), dominance × dominance (*dd*), effects were dissected. The dominance degree of each QTL or interaction was separately calculated by |*d*_*1*_/*a*_*1*_| and |*d*_*1*_*d*_*2*_/*a*_*1*_*a*_*2*_|, respectively. Where *d*_*1*_, *a*_*1*_, *d*_*1*_*d*_*2*_, *a*_*1*_*a*_*2*_ denote the dissected dominance effect, additive effect, dominance × dominance epistatic effect, additive × additive epistatic effect, respectively. According to Stuber et al. [12], main effect QTL can be classified as additive (|*d*_*1*_/*a*_*1*_|<0.2), partial dominance (0.2≤|*d*_*1*_/*a*_*1*_|<0.8), dominance (0.8≤|*d*_*1*_/*a*_*1*_|<1.2), and overdominance (|*d*_*1*_/*a*_*1*_|≥1.2). Epistatic QTL can be classified as additive (|*d*_*1*_*d*_*2*_/*a*_*1*_*a*_*2*_|<0.2), partial dominance (0.2≤|*d*_*1*_*d*_*2*_/*a*_*1*_*a*_*2*_|<0.8), dominance (0.8≤|*d*_*1*_*d*_*2*_/*a*_*1*_*a*_*2*_|<1.2), and overdominance (|*d*_*1*_*d*_*2*_/*a*_*1*_*a*_*2*_|≥1.2).

**Table 7:** Dissected main and epistatic effects in *II* hybrid.

Trait	Chr^a^	Marker	Chr^a^	Marker	QTL_i_		QTL_j_		QTL_i_×QTL_j_				Dominance degree^b^
					*a*_*1*_	*d*_*1*_	*a*_*2*_	*d*_*2*_	*a*_*1*_*a*_*2*_	*a*_*1*_*d*_*2*_	*d*_*1*_*a*_*2*_	*d*_*1*_*d*_*2*_	
HD	12	C996			2.09	0.00							A
HD	8	C347	4	C56	0.46	-1.84	-0.46	-1.84	0.79	-0.46	0.46	4.47	OD
HD	1	R2632	10	RM258	-0.39	0.33	0.39	0.33	0.33	2.42	1.64	-0.33	D
HD	2	RZ599	11	R3203	0.04	-1.89	-0.04	-1.89	-1.89	-0.04	0.04	1.89	D
HD	3	RM227	8	C1121	-0.27	-1.56	0.27	-1.56	-1.56	0.27	-0.27	1.56	D
HD	6	Waxy	6	R2549	-0.83	-0.15	0.83	-0.15	-0.15	0.83	-0.83	0.15	D
PH	7	RM70			1.92	0.00							A
PH	3	R1925			0.00	1.86							OD
PH	5	RG360	8	R1629	0.24	-2.44	-0.24	-2.44	-2.44	-0.24	0.24	2.44	D
PH	6	RM204	6	C962	1.67	-0.26	-1.67	-0.26	-0.26	-1.67	1.67	0.26	D
TP	2	RM53			0.00	0.38							OD
TP	4	R78	12	C909B	-0.07	0.33	0.07	0.33	-0.27	0.07	-0.07	-0.92	OD
TP	4	C2807	6	P	-0.01	0.60	0.01	0.60	0.60	0.01	-0.01	-0.60	D
TP	2	RG634	2	R1738	-0.81	0.09	0.81	0.09	0.09	0.81	-0.81	-0.09	D
PL	6	G342			0.30	0.00							A
PL	4	G235	7	RM70	-0.19	-0.46	0.19	-0.46	0.13	0.19	-0.19	1.06	OD
PL	3	C316	8	RM25	-0.14	-0.41	0.14	-0.41	-0.41	0.14	-0.14	0.41	D
PL	6	RM204	12	C909B	-0.28	0.20	0.28	0.20	0.20	0.28	-0.28	-0.20	D
FGPP	5	R3166	6	RZ667	-1.39	5.65	1.39	5.65	-2.64	1.39	-1.39	-13.94	OD
FGPP	8	RM223	9	RG570	-0.78	0.17	0.78	0.17	0.17	-2.92	-4.48	-0.17	D
FGPP	2	RM48	9	R1687	1.03	3.97	-1.03	3.97	3.97	-1.03	1.03	-3.97	D
FGPP	8	C1121	8	RG978	-3.82	0.00	3.82	0.00	0.00	3.82	-3.82	0.00	D
SS	12	RM20b			2.62	0.00							A
SS	6	RG424	13	C933	-0.76	-0.97	0.76	-0.97	-0.97	3.13	1.61	0.97	D
SS	2	G1314a	6	R2549	-1.00	2.94	1.00	2.94	2.94	1.00	-1.00	-2.94	D
SS	9	C153B	14	C477	-7.01	-1.55	7.01	-1.55	-1.55	7.01	-7.01	1.55	D
GD	5	R3166	6	RZ667	-0.05	0.19	0.05	0.19	-0.12	0.05	-0.05	-0.50	OD
GD	1	C161	1	RM243	0.05	0.22	-0.05	0.22	0.22	-0.05	0.05	-0.22	D
GD	2	RM48	9	R1687	0.03	0.17	-0.03	0.17	0.17	-0.03	0.03	-0.17	D
GD	2	RZ324	7	RM234	-0.12	0.08	0.12	0.08	0.08	0.12	-0.12	-0.08	D
KGW	3	C1176			0.00	0.33							OD
KGW	8	L363A			0.00	0.22							OD
KGW	5	RG360	8	C347	0.02	-0.01	-0.02	-0.01	-0.01	-0.41	-0.37	0.01	D
KGW	8	RM25	9	RZ404	0.18	0.40	-0.18	0.40	-0.60	-0.18	0.18	-1.39	OD
KGW	1	RG101	4	RM241	-0.13	0.33	0.13	0.33	0.33	0.13	-0.13	-0.33	D
KGW	1	G393	1	R2201	0.75	-0.87	-0.75	-0.87	-0.87	-0.75	0.75	0.87	D
YD	10	C153A			1.50	0.00							A
YD	7	R1789			0.00	1.67							OD
YD	4	C2807	14	C477	-0.08	0.32	0.08	0.32	0.32	-1.86	-2.02	-0.32	D
YD	8	R1394	9	RM215	-0.77	1.99	0.77	1.99	1.99	0.77	-0.77	-1.99	D
YD	9	R1952b	10	C153A	1.37	0.01	-1.37	0.01	0.01	-1.37	1.37	-0.01	D
YD	11	L1044	11	Y6854L	4.53	1.02	-4.53	1.02	1.02	-4.53	4.53	-1.02	D

^a^ Chromosome where the detected QTL located in.

^b^Main effect QTL can be classified as additive (A) (|*d*_*1*_/*a*_*1*_|<0.2), partial dominance (PD) (0.2≤|*d*_*1*_/*a*_*1*_|<0.8), dominance (D) (0.8≤|*d*_*1*_/*a*_*1*_|<1.2), and overdominance (OD) (|*d*_*1*_/*a*_*1*_|≥1.2). Epistatic QTL can be classified as additive (A) (|*d*_*1*_*d*_*2*_/*a*_*1*_*a*_*2*_|<0.2), partial dominance (PD) (0.2≤|*d*_*1*_*d*_*2*_/*a*_*1*_*a*_*2*_|<0.8), dominance (D) (0.8≤|*d*_*1*_*d*_*2*_/*a*_*1*_*a*_*2*_|<1.2), and overdominance (OD) (|*d*_*1*_*d*_*2*_/*a*_*1*_*a*_*2*_|≥1.2).

**Table 8:** Dissected main and epistatic effects in *IJ* hybrid.

Trait	Chr^a^	Marker	Chr^a^	Marker	QTL_i_		QTL_j_		QTL_i_×QTL_j_				Dominance degree^b^
					*a*_*1*_	*d*_*1*_	*a*_*2*_	*d*_*2*_	*a*_*1*_*a*_*2*_	*a*_*1*_*d*_*2*_	*d*_*1*_*a*_*2*_	*d*_*1*_*d*_*2*_	
HD	1	RG811			0.73	0.00							A
HD	3	XNPB249			0.83	0.00							A
HD	3	CDO1081			0.72	0.00							A
HD	7	RG711			0.60	0.00							A
HD	8	RG333			2.73	0.96							PD
HD	5	RZ556	8	RZ562	0.80	0.55	0.80	0.55	0.00	0.80	0.80	1.10	OD
HD	8	RZ562	9	RG667	0.85	1.01	0.85	1.01	1.01	0.85	0.85	1.01	D
HD	2	TW500	5	RG480	0.43	0.24	0.43	0.24	0.24	0.43	0.43	0.24	D
HD	6	RZ450	6	CDO204	6.66	0.00	6.66	0.00	0.00	6.66	6.66	0.00	D
HD	8	RG333	11	CDO534	3.80	0.28	1.07	0.45	0.45	1.07	1.07	0.45	D
PH	8	RG333			3.47	0.00							A
PH	7	CDO533			0.00	1.48							OD
PH	2	RZ987	10	RZ892	0.26	1.34	0.26	1.34	1.28	0.26	0.26	3.95	OD
PH	2	RG152	4	XNPB271	0.12	0.79	0.12	0.79	2.21	0.12	0.12	3.79	OD
PH	2	TW500	3	RG510	0.03	1.35	0.03	1.35	1.35	0.03	0.03	1.35	D
PH	1	RG173	2	TW500	1.15	1.02	1.15	1.02	1.02	1.15	1.15	1.02	D
PH	4	RG449	7	RG711	1.63	0.03	1.63	0.03	0.03	1.63	1.63	0.03	D
PH	5	RZ390	9	RZ12	0.90	0.31	0.90	0.31	0.31	0.90	0.90	0.31	D
PH	5	RZ70	8	RZ562	1.68	0.18	1.68	0.18	0.18	1.68	1.68	0.18	D
TP	1	RG541	7	RZ626	0.01	0.24	0.01	0.24	0.07	0.01	0.01	0.41	OD
TP	9	RZ422	11	XNPB179	0.04	0.25	0.04	0.25	0.05	0.04	0.04	0.45	OD
TP	2	RG555	7	CDO405	0.15	0.00	0.15	0.00	0.00	0.34	0.04	0.00	D
TP	6	RZ450	9	RZ12	0.03	0.05	0.03	0.05	0.06	0.12	0.18	0.06	D
TP	2	TW500	6	RZ828	0.04	0.29	0.04	0.29	0.29	0.04	0.04	0.29	D
TP	3	RG1356	6	RG213	0.12	0.00	0.12	0.00	0.00	0.12	0.12	0.00	D
TP	4	RG908	9	XNPB295	0.10	0.08	0.10	0.08	0.08	0.10	0.10	0.08	D
PL	5	RZ296	11	CDO534	0.05	0.32	0.05	0.32	0.10	0.05	0.05	0.55	OD
PL	4	RZ262	12	XNPB189	0.12	0.45	0.12	0.45	0.45	0.12	0.12	0.45	D
PL	4	RG449	11	CDO534	0.12	0.31	0.12	0.31	0.31	0.12	0.12	0.31	D
PL	9	RZ404	12	RG98	0.30	0.12	0.30	0.12	0.12	0.30	0.30	0.12	D
FGPP	3	CDO1081			6.12	0.00							A
FGPP	1	RG541	4	XNPB271	1.76	0.66	1.76	0.66	0.66	4.93	1.41	0.66	D
FGPP	1	RG469	5	RZ390	0.60	3.18	0.60	3.18	3.18	0.60	0.60	3.18	D
FGPP	1	RZ776	8	RG333	0.28	4.18	0.28	4.18	4.18	0.28	0.28	4.18	D
FGPP	1	RG375	10	RZ811	2.59	5.47	2.59	5.47	5.47	2.59	2.59	5.47	D
FGPP	2	RZ913	2	RG544	0.01	2.71	0.01	2.71	2.71	0.01	0.01	2.71	D
FGPP	2	CDO395	7	CDO405	0.22	4.98	0.22	4.98	4.98	0.22	0.23	4.98	D
FGPP	2	CDO395	8	RG333	0.22	2.14	0.22	2.14	2.14	0.22	0.22	2.14	D
FGPP	2	RZ987	6	WAXY	0.14	2.28	0.14	2.28	2.28	0.14	0.14	2.28	D
FGPP	2	RZ987	10	RZ892	2.27	4.02	2.27	4.02	4.02	2.27	2.27	4.02	D
FGPP	2	XNPB132	10	RZ811	1.97	3.42	1.97	3.42	3.42	1.97	1.97	3.42	D
FGPP	2	RG544	5	RG480	2.07	4.65	2.07	4.65	4.65	2.07	2.07	4.65	D
FGPP	2	TW500	8	RZ562	0.36	5.19	0.36	5.19	5.19	0.36	0.36	5.19	D
FGPP	3	XNPB249	8	RG136	0.19	2.33	0.19	2.33	2.33	0.19	0.19	2.33	D
FGPP	3	RZ16	10	RZ561	0.59	3.01	0.59	3.01	3.01	0.59	0.59	3.01	D
FGPP	4	CDO456	11	XNPB320	1.55	3.61	1.55	3.61	3.61	1.55	1.55	3.61	D
FGPP	5	RG573	9	RG667	0.39	3.70	0.39	3.70	3.70	0.39	0.39	3.70	D
FGPP	6	RG433	8	RZ28	0.10	2.10	0.10	2.10	2.10	0.10	0.10	2.10	D
FGPP	7	CDO405	10	RZ561	1.95	4.59	1.95	4.59	4.59	1.95	1.95	4.59	D
FGPP	1	RG375	12	XNPB316	2.62	1.52	2.62	1.52	1.52	2.62	2.62	1.52	D
FGPP	1	CDO962	2	RZ987	2.53	1.08	2.53	1.08	1.08	2.53	2.53	1.08	D
FGPP	1	RG173	5	RG480	3.60	0.14	3.60	0.14	0.14	3.60	3.60	0.14	D
FGPP	3	CDO1081	12	RZ816	4.27	0.07	1.85	0.07	0.07	1.85	1.85	0.07	D
FGPP	4	CDO456	6	XNPB317	1.96	0.34	1.96	0.34	0.34	1.96	1.96	0.34	D
FGPP	7	RG528	11	RZ638	3.67	2.60	3.67	2.60	2.60	3.67	3.68	2.60	D
FGPP	8	RG333	9	RZ927	4.54	2.46	4.54	2.46	2.46	4.54	4.54	2.46	D
FGPP	9	XNPB103	12	RG901	3.96	0.31	3.96	0.31	0.31	3.96	3.96	0.31	D
FGPP	9	RZ12	9	RG667	7.70	0.86	7.70	0.86	0.86	7.70	7.70	0.86	D
FGPP	12	RZ816	12	XNPB189	7.07	6.83	7.07	6.83	6.83	7.07	7.07	6.83	D
SS	1	XNPB302	10	RZ811	0.66	0.38	0.66	0.38	0.37	0.38	1.71	0.37	D
SS	2	RG544	7	RG711	0.35	3.04	0.35	3.04	3.04	0.35	0.35	3.04	D
SS	7	RG528	10	RZ400	2.05	0.91	2.05	0.91	0.91	2.05	2.05	0.91	D
GD	6	RG162			0.00	0.16							OD
GD	1	RG375	10	RZ811	0.10	0.29	0.10	0.29	0.11	0.10	0.10	0.48	OD
GD	2	CDO395	7	CDO405	0.03	0.33	0.03	0.33	0.33	0.03	0.03	0.33	D
GD	2	RZ599	6	RZ965	0.16	0.25	0.16	0.25	0.25	0.16	0.16	0.25	D
GD	4	RZ590	9	RZ12	0.23	0.03	0.23	0.03	0.03	0.23	0.23	0.03	D
KGW	5	RZ296			0.91	0.00							A
KGW	5	CDO202			0.00	0.41							OD
KGW	2	RG555	9	RG358	0.50	1.33	0.50	1.33	1.33	0.50	0.50	1.33	D
KGW	4	RG908	8	RG333	1.84	2.63	1.84	2.63	2.63	1.84	1.84	2.63	D
YD	8	RZ562			0.00	191.74							OD
YD	2	RZ825	5	RG480	177.88	378.17	177.88	378.17	378.17	177.88	177.88	378.17	D
YD	7	CDO405	10	RZ400	166.44	544.18	166.44	544.18	544.18	166.44	166.44	544.18	D
YD	4	RG908	8	RG333	481.55	686.50	481.55	686.50	686.50	481.55	481.55	686.50	D

^a^ See footnote of Table 7.

^b^ See footnote of Table 7.

HD: In the *II* hybrid, the only main effect QTL was classified as additive; in the other five epistatic QTL, most were classified as dominance, except one that showed overdominance. In the *IJ* hybrid, four main effect QTL were classified as additive, and only one main effect QTL was classified as partial dominance; four epistatic QTL were classified as dominance, and one epistatic QTL was classified as overdominance.

PH: In the *II* hybrid, two main effect QTL were classified as additive and overdominance, respectively; two epistatic QTL were classified as dominance. In the *IJ* hybrid, two main effect QTL were classified as additive and overdominance, respectively; in seven epistatic QTL, two were classified as overdominance and the other five were dominance.

Tillers per plant: In the *II* hybrid, the only main effect QTL was classified as overdominance; two of three epistatic QTL were classified as dominance and the remaining one was classified as overdominance. In the *IJ* hybrid, no main effect QTL was found; in seven epistatic QTL, two were classified as overdominance and the remaining five were dominance, which was similar to trait PH.

PL: In the *II* hybrid, the only main effect QTL was classified as additive; two of three epistatic QTL were classified as dominance and the remaining one showed overdominance. In the *IJ* hybrid, no main effect QTL was found; in four epistatic QTL, one was identified as overdominance and the rest were dominance.

FGPP: In the *II* hybrid, no main effect QTL was found; in four epistatic QTL, one was classified as overdominance and the rest were dominance. In the *IJ* hybrid, the only main effect QTL was classified as additive; a total of 28 epistatic QTL was dissected, all of which showed dominance.

Percentage of seed set: In the *II* hybrid, the only main effect QTL was classified as additive and the three epistatic QTL were classified as dominance. In the *IJ* hybrid, only three epistatic QTL were dissected and all of them were classified as dominance.

GD: In the *II* hybrid, no main effect QTL was found; among four epistatic QTL, one was classified as overdominance and the remaining three were dominance. In the *IJ* hybrid, the only main effect QTL was classified as overdominance; among four epistatic QTL, three were classified as dominance and the remaining one was classified as overdominance.

KGW: In the *II* hybrid, two main effect QTL were classified as overdominance; in four epistatic QTL, one showed overdominance and the remaining three showed dominance. In the *IJ* hybrid, two main effect QTL were classified as additive and overdominance, respectively; two epistatic QTL showed dominance.

YD: In the *II* hybrid, two main effect QTL were classified as additive and overdominance, respectively; all four epistatic QTL were classified as dominance. In the *IJ* hybrid, the only main effect QTL showed overdominance, and the three epistatic QTL were classified as dominance.

From Tables 7 and 8, we can see that little common loci were found. This phenomenon partially results from the mapping markers in *IJ* and *II* hybrid are different. But on the same chromosome, we found some nearby loci affected same trait in both *II* and *IJ* hybrids.

Table 9 summarizes the main and epistatic QTL revealed in the *II* and *IJ* hybrids. For main effect QTL, 10 QTL were identified in the *II* hybrid; five were classified as additive, and the rest were classified as overdominance. In the *IJ* hybrid, 12 QTL were found, more additive (58.33%) loci were identified than overdominance in number (33.34%). For epistatic QTL, dominance or overdominance are found in two hybrid combinations, and dominance played a leading role in epistatic QTL. Dominance accumulation and overdominance were the major genetic basis of heterosis.

**Table 9:** Summary of main and epistatic effects in II and IJ hybrids.

	*II* Main-effect QTL		*IJ* Main-effect QTL		*II* Epistatic-effect QTL		*IJ* Epistatic-effect QTL	
	No.	Rate(%)	No.	Rate(%)	No.	Rate(%)	No.	Rate(%)
A	5	50	8	57.14				
PD			1	7.14				
D					26	81.25	56	86.15
OD	5	50	5	35.72	6	18.75	9	13.85
SUM	10		14		32		65	

## Discussion

### Models comparison

Based on the aTTC design, this paper developed a QTL mapping method that fit for many base populations (RIL, F_2_, and DH); by employing multiple data set transformations (Z_1i_, Z_2i_, Z_3i_, Z_4i_, Z_5i_, and Z_6i_), many types of main and epistatic effects can be dissected. This paper took one combination (Z_1_, Z_2_, and Z_4_) of the aTTC design as an instance and proposed a two-step approach to dissect additive, dominance, and epistatic effects of QTL in the RIL-based aTTC design. A series of Monte Carlo simulation studies were carried out to confirm the proposed approach. Compared to previous studies on our methodologies, the proposed approach offered great advantages over previous methods.

aTTC design has many more transformations than do the TTC or NCIII designs, and with a series of transformation combinations (Z_1_, Z_2_, and Z_4_), (Z_1_, Z_2_, and Z_5_), or (Z_1_, Z_2_, and Z_6_), we can dissect main and epistatic effects of individual QTL or interactions by QTL mapping. It provides a new method for quantitative genetics research and especially for allowing scientists and breeders to understand the genetic basis for plant heterosis. In our study, we took the transformation combination Z_1_, Z_2_, and Z_4_ of RIL-based aTTC design as an instance to dissect genetic effects. There were some advantages when taking RIL as the base population. The genetic expectation mean of RIL was equivalent to *L*_*4i*_; therefore, there was no need to self-mate the base population. We simply used the RIL population data set substitute *L*_*4i*_, which saved labor and time. When using RIL-based TTC design for QTL mapping, we need generate four populations RIL, L1, L2 and L3. However, when using RIL-based aTTC design, breeders only need generate three populations RIL, L1 and L2. With combination (Z_1_ = L1+L2, Z_2_ = L1−L2 and Z_4_ = L1+L2−L4), we can dissect additive, dominance, and epistatic effects of QTL with high statistical powers and accuracies. In addition, many real mapping populations that derived from RIL-based NCIII design can be re-analyzed by the proposed method to develop main and epistatic effects to clearly decipher a genetic basis for heterosis.

In the present study, we used three different interaction patterns in one genetic model, which was much more complicated than that proposed by He et al. [37,39]. As shown in Tables A and B in S11 Supporting Information, with high detection power, all the augmented main effects in QTL (QTL_1-6_) and epistatic effects in digenic interactions (QTL_1_ and QTL_2_, QTL_3_ and QTL_4_, and QTL_5_ and QTL_6_) were estimated in an unbiased manner in Z_1_ and Z_2_. In Z_4_, two augmented epistatic effects ${\vec{i}}_{12}=i_{a_{1}a_{2}}-i_{d_{1}d_{2}}$ and ${\overset{\leftarrow }{i}}_{12}=i_{a_{1}d_{2}}-i_{d_{1}a_{2}}$ were further estimated precisely (shown in Table 3).

Actually, for the detection of small and linked QTL, low powers were observed. EBLASSO can handle the model that includes many effects [37, 39, 43]. In this sudy, we use a large number of effects, including main and epistatic QTL effects, simultaneously. EBLASSO shrinks weak effect into zero, which has little influence on large effect QTL. Simulation studies demonstrated that the fast EBLASSO greatly improved calculated speed and detected more true QTL effects without increasing the false-positive rate.

### Comparison of QTL mapping results in *II* and *IJ* hybrid with previous mapping results

The QTL mapping results of this paper were compared with those of our previous study [24] in which the CIM was employed to mapping main effect QTL [44], and the mixed linear approach [45] was used to estimate epistatic QTL. QTL detected by both studies are listed in Tables 10 and 11 for *II* and *IJ* hybrids, respectively. As shown in Table 10, a total of nine main effects QTL and four epistatic QTL was found simultaneously in two studies; only one QTL revealed in trait PH showed opposite dominance degree. In an RIL mapping population, three main effects QTL were detected; two main effects QTL were detected in Z_1_, and both of them were identified as additive. Eight QTL were found in Z_2_, and half of them were main effects; dominance degree revealed by two methods was similar, except for marker R1925 in trait PH. For the *IJ* hybrid, shown in Table 11, no epistatic QTL was simultaneously detected. The number of main effects QTL detected by both studies was 17, 3, and 3 for RIL, Z_1_, and Z_2_, respectively. Except for marker CDO533 in trait PH, the detected main effects QTL showed the same dominance degree. If not taking threshold into consideration, the ratio of same main effect QTL detected by the fast EBLASSO algorithm to previous mapping results were 23.08%, 22.22%, and 50% for RIL, Z_1_, and Z_2_, respectively in the *II* hybrid, whereas in the *IJ* hybrid, they were 54.84%, 17.65%, and 21.430%.

**Table 10:** Comparison of the QTL mapping results by the proposed approach with previous results in Li et al. in *II* hybrid.

					RIL				Z_1_				Z_2_				Dominance degree^b^	
Trait	Chr^a^	Marker	Chr^a^	Marker	Method A^c^		Method B^d^		Method A		Method B		Method A		Method B		Method A	Method B
					beta	h^2^(%)^e^	beta	h^2^(%)^e^	beta	h^2^(%)^e^	beta	h^2^(%)^e^	beta	h^2^(%)^e^	beta	h^2^(%)^e^		
HD	12	C996							-3.10	6.10	-2.09	2.86					A	A
HD	1	R2632	10	RM258									2.11	6.27	2.03	5.91	OD	D
PH	10	RG561			2.27	3.6	1.27	2.16									A	-
PH	3	R1925											-2.86	4.60	-1.86	1.99	A	OD
PL	5	R3166			0.43	4.2	-0.30	1.21									A	-
PL	6	G342							0.62	5.20	0.30	1.21					A	A
FGPP	8	RM223	9	RG570									-3.75	3.08	-3.70	3.30	OD	D
SS	6	RG424	13	C933									2.18	3.56	2.37	4.45	OD	D
GD	6	R1014			0.2	5.5	0.10	1.64									A	-
KGW	3	C1176											0.42	3.80	-0.33	2.48	D	OD
KGW	8	L363A											0.50	5.50	-0.22	1.13	OD	OD
YD	7	R1789											-1.99	5.20	1.67	3.67	OD	OD
YD	4	C2807	14	C477									-2.77	8.24	-1.94	5.33	OD	D

^a^ See footnote of Table 7.

^b^ See footnote of Table 7.

^c^ Method A: Composite-interval mapping, with WinQTLcart (Zeng 1994) for main effect QTL mapping; Mixed linear approach, with QTLMAPPER ver.1.0 (Wang et al, 1999) for epistatic effect QTL mapping.

^d^ Method B: Proposed method in this paper.

^e^ Variation contributed by QTL or digenic interaction.

**Table 11:** Comparison of the QTL mapping results by the proposed approach with previous results in Li *et al* in *IJ* hybrid.

					RIL				Z_1_				Z_2_				Dominance degree^b^	
Trait	Chr^a^	Marker	Chr^a^	Marker	Method A^c^		Method B^d^		Method A		Method B		Method A		Method B		Method A	Method B
					beta	h^2^ (%)^e^	beta	h^2^ (%)^e^	beta	h^2^ (%)^e^	beta	h^2^ (%)^e^	beta	h^2^ (%)^e^	beta	h^2^ (%)^e^		
HD	3	CDO1081			1.71	7.9	1.62	7.08	1.10	6.80	0.72	2.94					PD	A
HD	8	RG333			-5.1	58.3	-3.46	27.48	-2.72	34.80	-2.73	36.58	-1.22	17.40	-0.96	10.93	PD	PD
PH	8	RG333			-5.1	58.3	-2.22	55.37	-4.81	15.80	-3.47	8.47					A	A
PH	7	CDO533											-3.79	29.00	-1.48	4.29	A	OD
TP	4	RG214			0.27	8.7	0.21	5.12									A	-
TP	5	CDO1160			0.2	5.1	0.11	1.53									OD	-
PL	7	RG528			-1.07	34.6	-0.21	1.26									A	-
PL	9	RG358			0.44	5.2	0.21	1.17									A	-
FGPP	3	CDO1081			-7.02	12.5	-5.60	7.97									A	-
FGPP	4	RG214			-7.8	15.7	-7.28	13.06									A	-
FGPP	5	RG360			5.96	9	4.98	5.95									PD	-
SS	5	RG360			2.9	7.2	2.65	5.68									A	-
GD	3	RZ993			-0.3	11.5	-0.11	1.66									A	-
GD	4	RZ590			-0.38	17.8	-0.37	16.70									PD	-
GD	10	RZ561			0.2	5	0.14	2.17									A	-
KGW	8	RG333			3.07	15.5	2.86	14.55									OD	-
YD	3	CDO1081			-0.49	7.2	-0.45	6.03									A	-
YD	8	RZ562			0.66	11.3	0.56	8.09					0.17	5.90	0.19	4.17	OD	-

^a^ See footnote of Table 7.

^b^ See footnote of Table 7.

^c^ See footnote of Table 10.

^d^ See footnote of Table 10.

^e^ See footnote of Table 10.

Among the identified QTL, some of them were pleiotropic. In the *IJ* hybrid, marker RG333 on chromosome 8 was simultaneously revealed in traits HD, PH, KGW, and YD; marker CDO1081 on chromosome 3 was simultaneously identified for traits HD, FGPP, and YD. These markers, especially for marker RG333 and marker CDO1081, were also found pleiotropic in the work of Xiao et al. [8] and Li et al. [24]. These regions deserve further attention, especially in marker-assisted breeding.

### Genetic basis of heterosis with real data analysis

With our proposed approach, we dissected genetic effects of QTL and interactions for the *II* and *IJ* hybrids, respectively, and calculated the dominance degree of each QTL or digenic interaction (Tables 7 and 8). We summarized the classified dominance degree of real mapping populations (Table 9) and found that dominance degree in the Z_2_ data set that mainly characterized the heterosis showed overdominance and dominance for QTL and digenic interactions, and the ratio of dominance is greater than overdominance. Therefore, we conclude that dominance accumulation and overdominance are the major genetic basis of heterosis. This finding is consistent with Huang et al. [4], who pointed out that the accumulation of numerous rare superior alleles with positive dominance was an important contributor to heterotic phenomena after genomic analysis of hybrid rice varieties.

To explicitly elucidate the influence of single-locus (additive and dominance) and two-loci (*aa*, *ad*, *da*, and *dd* epistatic effect) genetic effects conditioning the heterosis of agronomic traits, models or genetic mating design (e.g., RIL-based TTC design) [30, 38], which can be used to study how interactions among multiple genes can lead to the phenotypic manifestations of heterosis, are probably the most relevant. Recent findings from genomic, proteomic, metabolic, epigenetic, and network studies in hybrids and polyploids also highlight some testable models for heterosis [46].

## Supporting information

S1 Supporting Information Expected genetic values of *Z*_*1i*_, *Z*_*2i*_, *Z*_*3i*_, *Z*_*4i*_, *Z*_*5i*_ and *Z*_*6i*_ under both the F_∞_ and F_2_ metric models in RIL-based aTTC design. (DOCX)

S2 Supporting Information Expected genetic values of *Z*_*1i*_, *Z*_*2i*_, *Z*_*3i*_, *Z*_*4i*_, *Z*_*5i*_ and *Z*_*6i*_ under both the F_∞_ and F_2_ metric models in F_2_-based aTTC design. (DOCX)

S3 Supporting Information Statistical genetic models for mapping heterotic QTL in the RIL-based aTTC design under the F_2_ metric model. (DOC)

S4 Supporting Information Statistical genetic models for mapping heterotic QTL in the F_2_-based aTTC design under the F_∞_ metric model. (DOC)

S5 Supporting Information Simulation data generate script. (ZIP)

S6 Supporting Information Simulation data of Z_1_. (ZIP)

S7 Supporting Information Simulation data of Z_2_. (ZIP)

S8 Supporting Information Simulation data of Z_4_. (ZIP)

S9 Supporting Information Simulation data of Z_5_. (ZIP)

S10 Supporting Information Simulation data of Z_6_. (ZIP)

S11 Supporting Information Simulation results of Z_1_,Z_2_,Z_5_,Z_6_. (DOC)

S12 Supporting Information Real data augument effect results. (DOC)

S1 Fig The mean statistic power of augmented main and epistatic effect interactions in Z_1_. (TIF)

S2 Fig The mean statistic power of augmented main and epistatic effect interactions in Z_2_. (TIF)

S3 Fig The mean statistic power of augmented main and epistatic effect interactions in Z_5_. (TIF)

S4 Fig The mean statistic power of augmented main and epistatic effect interactions in Z_6_. (TIF)
