# Correction: Genetic dissection of main and epistatic effects of QTL based on augmented triple test cross design

**DOI:** 10.1371/journal.pone.0198562

**Published:** 2018-05-30

**Authors:** Xueli Zhang, Congwei Sun, Zheng Zhang, Zhijun Dai, Yuan Chen, Xiong Yuan, Zheming Yuan, Wenbang Tang, Lanzhi Li, Zhongli Hu

There is an error in affiliation 2 for authors Xueli Zhang, Congwei Sun, Zheng Zhang, Zhijun Dai, Yuan Chen, Xiong Yuan, Zheming Yuan, and Lanzhi Li. Affiliation 2 should be: Hunan Provincial Key Laboratory for Biology and Control of Plant Diseases and Insect Pests.

## References

[1] Zhang X, Sun C, Zhang Z, Dai Z, Chen Y, Yuan X, et al (2017) Genetic dissection of main and epistatic effects of QTL based on augmented triple test cross design. PLoS ONE 12(12): e0189054 https://doi.org/10.1371/journal.pone.0189054 29240818 10.1371/journal.pone.0189054 PMC5730204

