# Supplementary material for: Genetic dissection of main and epistatic effects of QTL based on augmented triple test cross design
Source: PLoS One. 2017 Dec 14;12(12):e0189054. doi: 10.1371/journal.pone.0189054 (PMC5730204; doi:10.1371/journal.pone.0189054)
Supplement: S1 Supporting Information — (DOCX) [file pone.0189054.s001.docx]

**Expected genetic values of *Z_1i_*, *Z_2i_*, *Z_3i_*, *Z_4i_*, *Z_5i_* and *Z_6i_* under both the F_∞_ and F_2_ metric models in RIL-based aTTC design**

We assume that a quantitative trait is controlled by two QTL, *A* and *B*, each with two alleles (*A*, *a*; *B*, *b*). The recombination fraction between A and B was *r*. The genotypes of the two inbred lines (*P_1_* and *P_2_*) are *AABB* and *aabb*, respectively. For the RIL population, there are four homozygotes, *AABB*, *AAbb*, *aaBB* and *aabb*, with frequency (1−*R*)/2, *R*/2, *R*/2 and (1−*R*)/2, respectively, where *R* = 2*r*/(1+2*r*). In aTTC design, individuals from the RIL population are crossed to *P_1_* (with gamete *AB*) and *P_2_* (with gamete *ab*) and *F_1_* to produce *L_1_* and *L_2_* and *L_3_* families, at the same time, the *n* individuals in base population were self-mated to get *L_4_*.The expected genetic values of *L_1i_*, *L_2i_*, *L_3i_* and *L_4i_* under the F_∞_ and F_2_ metric model in RIL-based aTTC design are presented in Table A1, Table A2, Table A3 and Table A4, respectively.

**Table A1. Expected genetic values of *L_1i_* under the F_∞_ and F_2_ metric model in RIL-based aTTC design.**

| Genotype of  RIL | Frequency | F_∞_ | | | | | | | | |  | F_2_ | | | | | | | | |
| --- | --- | --- | --- | --- | --- | --- | --- | --- | --- | --- | --- | --- | --- | --- | --- | --- | --- | --- | --- | --- |
|  |  | $\mu$ | $a_{1}$ | $d_{1}$ | $a_{2}$ | $d_{2}$ |  |  |  |  |  | $\mu$ | $a_{1}$ | $d_{1}$ | $a_{2}$ | $d_{2}$ |  |  |  |  |
| $\frac{\begin{matrix} A & B \end{matrix}}{\begin{matrix} A & B \end{matrix}}$ | (1-*R*)/2 | 1 | 1 | 0 | 1 | 0 | 1 | 0 | 0 | 0 |  | 1 | 1 | -1/2 | 1 | -1/2 | 1 | -1/2 | -1/2 | 1/4 |
| $\frac{\begin{matrix} A & b \end{matrix}}{\begin{matrix} A & b \end{matrix}}$ | *R*/2 | 1 | 1 | 0 | 0 | 1 | 0 | 1 | 0 | 0 |  | 1 | 1 | -1/2 | 0 | 1/2 | 0 | 1/2 | 0 | -1/4 |
| $\frac{\begin{matrix} a & B \end{matrix}}{\begin{matrix} a & B \end{matrix}}$ | *R*/2 | 1 | 0 | 1 | 1 | 0 | 0 | 0 | 1 | 0 |  | 1 | 0 | 1/2 | 1 | -1/2 | 0 | 0 | 1/2 | -1/4 |
| $\frac{\begin{matrix} a & b \end{matrix}}{\begin{matrix} a & b \end{matrix}}$ | (1-*R*)/2 | 1 | 0 | 1 | 0 | 1 | 0 | 0 | 0 | 1 |  | 1 | 0 | 1/2 | 0 | 1/2 | 0 | 0 | 0 | 1/4 |

**Table A2. Expected genetic values of *L_2i_* under the F_∞_ and F_2_ metric model in RIL-based aTTC design.**

| Genotype of  RIL | Frequency | F_∞_ | | | | | | | | |  | F_2_ | | | | | | | | |
| --- | --- | --- | --- | --- | --- | --- | --- | --- | --- | --- | --- | --- | --- | --- | --- | --- | --- | --- | --- | --- |
|  |  | $\mu$ | $a_{1}$ | $d_{1}$ | $a_{2}$ | $d_{2}$ |  |  |  |  |  | $\mu$ | $a_{1}$ | $d_{1}$ | $a_{2}$ | $d_{2}$ |  |  |  |  |
| $\frac{\begin{matrix} A & B \end{matrix}}{\begin{matrix} A & B \end{matrix}}$ | (1-*R*)/2 | 1 | 0 | 1 | 0 | 1 | 0 | 0 | 0 | 1 |  | 1 | 0 | 1/2 | 0 | 1/2 | 0 | 0 | 0 | 1/4 |
| $\frac{\begin{matrix} A & b \end{matrix}}{\begin{matrix} A & b \end{matrix}}$ | *R*/2 | 1 | 0 | 1 | -1 | 0 | 0 | 0 | -1 | 0 |  | 1 | 0 | 1/2 | -1 | -1/2 | 0 | 0 | -1/2 | -1/4 |
| $\frac{\begin{matrix} a & B \end{matrix}}{\begin{matrix} a & B \end{matrix}}$ | *R*/2 | 1 | -1 | 0 | 0 | 1 | 0 | -1 | 0 | 0 |  | 1 | -1 | -1/2 | 0 | 1/2 | 0 | -1/2 | 0 | -1/4 |
| $\frac{\begin{matrix} a & b \end{matrix}}{\begin{matrix} a & b \end{matrix}}$ | (1-*R*)/2 | 1 | -1 | 0 | -1 | 0 | 1 | 0 | 0 | 0 |  | 1 | -1 | -1/2 | -1 | -1/2 | 1 | 1/2 | 1/2 | 1/4 |

**Table A3. Expected genetic values of *L_3i_* under the F_∞_ and F_2_ metric model in RIL-based aTTC design.**

| Genotype of  RIL | Frequency | F_∞_ | | | | | | | | |  | F_2_ | | | | | | | | |
| --- | --- | --- | --- | --- | --- | --- | --- | --- | --- | --- | --- | --- | --- | --- | --- | --- | --- | --- | --- | --- |
|  |  | $\mu$ | $a_{1}$ | $d_{1}$ | $a_{2}$ | $d_{2}$ |  |  |  |  |  | $\mu$ | $a_{1}$ | $d_{1}$ | $a_{2}$ | $d_{2}$ |  |  |  |  |
| $\frac{\begin{matrix} A & B \end{matrix}}{\begin{matrix} A & B \end{matrix}}$ | (1-*R*)/2 | 1 | 1/2 | 1/2 | 1/2 | 1/2 | (1-r)/2 | r/2 | r/2 | (1-r)/2 |  | 1 | 1/2 | 0 | 1/2 | 0 | (1-r)/2 | -(1-2r)/4 | -(1-2r)/4 | (1-2r)/4 |
| $\frac{\begin{matrix} A & b \end{matrix}}{\begin{matrix} A & b \end{matrix}}$ | *R*/2 | 1 | 1/2 | 1/2 | -1/2 | 1/2 | -r/2 | (1-r)/2 | -(1-r)/2 | r/2 |  | 1 | 1/2 | 0 | -1/2 | 0 | -r/2 | (1-2r)/4 | -(1-2r)/4 | -(1-2r)/4 |
| $\frac{\begin{matrix} a & B \end{matrix}}{\begin{matrix} a & B \end{matrix}}$ | *R*/2 | 1 | -1/2 | 1/2 | 1/2 | 1/2 | -r/2 | -(1-r)/2 | (1-r)/2 | r/2 |  | 1 | -1/2 | 0 | 1/2 | 0 | -r/2 | -(1-2r)/4 | (1-2r)/4 | -(1-2r)/4 |
| $\frac{\begin{matrix} a & b \end{matrix}}{\begin{matrix} a & b \end{matrix}}$ | (1-*R*)/2 | 1 | -1/2 | 1/2 | -1/2 | 1/2 | (1-r)/2 | -r/2 | -r/2 | (1-r)/2 |  | 1 | -1/2 | 0 | -1/2 | 0 | (1-r)/2 | (1-2r)/4 | (1-2r)/4 | (1-2r)/4 |

**Table A4. Expected genetic values of *L_4i_* under the F_∞_ and F_2_ metric model in RIL-based aTTC design.**

| Genotype of  RIL | Frequency | F_∞_ | | | | | | | | |  | F_2_ | | | | | | | | |
| --- | --- | --- | --- | --- | --- | --- | --- | --- | --- | --- | --- | --- | --- | --- | --- | --- | --- | --- | --- | --- |
|  |  | $\mu$ | $a_{1}$ | $d_{1}$ | $a_{2}$ | $d_{2}$ |  |  |  |  |  | $\mu$ | $a_{1}$ | $d_{1}$ | $a_{2}$ | $d_{2}$ |  |  |  |  |
| $\frac{\begin{matrix} A & B \end{matrix}}{\begin{matrix} A & B \end{matrix}}$ | (1-*R*)/2 | 1 | 1 | 0 | 1 | 0 | 1 | 0 | 0 | 0 |  | 1 | 1 | 0 | 1 | 0 | 1 | 0 | 0 | 0 |
| $\frac{\begin{matrix} A & b \end{matrix}}{\begin{matrix} A & b \end{matrix}}$ | *R*/2 | 1 | 1 | 0 | -1 | 0 | -1 | 0 | 0 | 0 |  | 1 | 1 | 0 | -1 | 0 | -1 | 0 | 0 | 0 |
| $\frac{\begin{matrix} a & B \end{matrix}}{\begin{matrix} a & B \end{matrix}}$ | *R*/2 | 1 | -1 | 0 | 1 | 0 | -1 | 0 | 0 | 0 |  | 1 | -1 | 0 | 1 | 0 | -1 | 0 | 0 | 0 |
| $\frac{\begin{matrix} a & b \end{matrix}}{\begin{matrix} a & b \end{matrix}}$ | (1-*R*)/2 | 1 | -1 | 0 | -1 | 0 | 1 | 0 | 0 | 0 |  | 1 | -1 | 0 | -1 | 0 | 1 | 0 | 0 | 0 |

where $\mu$ is model mean (mean genotypic values of four homozygotes, *AABB*, *AAbb*, *aaBB* and *aabb*); $a_{1}$ is the additive effect of QTL *A*, i.e. average substitution effect of *a* (in *aaBB* and *aabb*) by *A*; $d_{1}$ is the dominance effect of QTL *A*, i.e. the departure in genotypic value of the heterozygote mean of *Aa* (*AaBB* and *Aabb*) from the midpoint between the two homozygote means of *AA* (*AABB* and *AAbb*) and *aa* (*aaBB* and *aabb*); $a_{2}$ and $d_{2}$ can be similarly defined;, , and are additive × additive, additive × dominance, dominance × additive and dominance × dominance epistatic effects between QTL *A* and *B*.

The expected genetic values of *Z_1i_*, *Z_2i_*, *Z_3i_*, *Z_4i_*, *Z_5i_* and *Z_6i_* under both the F_∞_ and F_2_ metric models in RIL-based aTTC design are presented in Table A5, Table A6, Table A7, Table A8, Table A9 and Table A10, respectively.

**Table A5. Expected genetic values of *Z_1i_* under the F_∞_ and F_2_ metric model in RIL-based aTTC design.**

| Genotype of  RIL | Frequency | F_∞_ | | | | | | | | |  | F_2_ | | | | | | | | |
| --- | --- | --- | --- | --- | --- | --- | --- | --- | --- | --- | --- | --- | --- | --- | --- | --- | --- | --- | --- | --- |
|  |  | $\mu$ | $a_{1}$ | $d_{1}$ | $a_{2}$ | $d_{2}$ |  |  |  |  |  | $\mu$ | $a_{1}$ | $d_{1}$ | $a_{2}$ | $d_{2}$ |  |  |  |  |
| $\frac{\begin{matrix} A & B \end{matrix}}{\begin{matrix} A & B \end{matrix}}$ | (1-*R*)/2 | 2 | 1 | 1 | 1 | 1 | 1 | 0 | 0 | 1 |  | 2 | 1 | 0 | 1 | 0 | 1 | -1/2 | -1/2 | 1/2 |
| $\frac{\begin{matrix} A & b \end{matrix}}{\begin{matrix} A & b \end{matrix}}$ | *R*/2 | 2 | 1 | 1 | -1 | 1 | 0 | 1 | -1 | 0 |  | 2 | 1 | 0 | -1 | 0 | 0 | 1/2 | -1/2 | -1/2 |
| $\frac{\begin{matrix} a & B \end{matrix}}{\begin{matrix} a & B \end{matrix}}$ | *R*/2 | 2 | -1 | 1 | 1 | 1 | 0 | -1 | 1 | 0 |  | 2 | -1 | 0 | 1 | 0 | 0 | -1/2 | 1/2 | -1/2 |
| $\frac{\begin{matrix} a & b \end{matrix}}{\begin{matrix} a & b \end{matrix}}$ | (1-*R*)/2 | 2 | -1 | 1 | -1 | 1 | 1 | 0 | 0 | 1 |  | 2 | -1 | 0 | -1 | 0 | 1 | 1/2 | 1/2 | 1/2 |

**Table A6. Expected genetic values of *Z_2i_* under the F_∞_ and F_2_ metric model in RIL-based aTTC design.**

| Genotype of  RIL | Frequency | F_∞_ | | | | | | | | |  | F_2_ | | | | | | | | |
| --- | --- | --- | --- | --- | --- | --- | --- | --- | --- | --- | --- | --- | --- | --- | --- | --- | --- | --- | --- | --- |
|  |  | $\mu$ | $a_{1}$ | $d_{1}$ | $a_{2}$ | $d_{2}$ |  |  |  |  |  | $\mu$ | $a_{1}$ | $d_{1}$ | $a_{2}$ | $d_{2}$ |  |  |  |  |
| $\frac{\begin{matrix} A & B \end{matrix}}{\begin{matrix} A & B \end{matrix}}$ | (1-*R*)/2 | 0 | 1 | -1 | 1 | -1 | 1 | 0 | 0 | -1 |  | 0 | 1 | -1 | 1 | -1 | 1 | -1/2 | -1/2 | 0 |
| $\frac{\begin{matrix} A & b \end{matrix}}{\begin{matrix} A & b \end{matrix}}$ | *R*/2 | 0 | 1 | -1 | 1 | 1 | 0 | 1 | 1 | 0 |  | 0 | 1 | -1 | 1 | 1 | 0 | 1/2 | 1/2 | 0 |
| $\frac{\begin{matrix} a & B \end{matrix}}{\begin{matrix} a & B \end{matrix}}$ | *R*/2 | 0 | 1 | 1 | 1 | -1 | 0 | 1 | 1 | 0 |  | 0 | 1 | 1 | 1 | -1 | 0 | 1/2 | 1/2 | 0 |
| $\frac{\begin{matrix} a & b \end{matrix}}{\begin{matrix} a & b \end{matrix}}$ | (1-*R*)/2 | 0 | 1 | 1 | 1 | 1 | -1 | 0 | 0 | 1 |  | 0 | 1 | 1 | 1 | 1 | -1 | -1/2 | -1/2 | 0 |

**Table A7. Expected genetic values of *Z_3i_* under the F_∞_ and F_2_ metric model in RIL-based aTTC design.**

| Genotype of  RIL | Frequency | F_∞_ | | | | | | | | |  | F_2_ | | | | | | | | |
| --- | --- | --- | --- | --- | --- | --- | --- | --- | --- | --- | --- | --- | --- | --- | --- | --- | --- | --- | --- | --- |
|  |  | $\mu$ | $a_{1}$ | $d_{1}$ | $a_{2}$ | $d_{2}$ |  |  |  |  |  | $\mu$ | $a_{1}$ | $d_{1}$ | $a_{2}$ | $d_{2}$ |  |  |  |  |
| $\frac{\begin{matrix} A & B \end{matrix}}{\begin{matrix} A & B \end{matrix}}$ | (1-*R*)/2 | 0 | 0 | 0 | 0 | 0 | r | -r | -r | r |  | 0 | 0 | 0 | 0 | 0 | r | -r | -r | r |
| $\frac{\begin{matrix} A & b \end{matrix}}{\begin{matrix} A & b \end{matrix}}$ | *R*/2 | 0 | 0 | 0 | 0 | 0 | r | r | -r | -r |  | 0 | 0 | 0 | 0 | 0 | r | r | -r | -r |
| $\frac{\begin{matrix} a & B \end{matrix}}{\begin{matrix} a & B \end{matrix}}$ | *R*/2 | 0 | 0 | 0 | 0 | 0 | r | -r | r | -r |  | 0 | 0 | 0 | 0 | 0 | r | -r | r | -r |
| $\frac{\begin{matrix} a & b \end{matrix}}{\begin{matrix} a & b \end{matrix}}$ | (1-*R*)/2 | 0 | 0 | 0 | 0 | 0 | r | r | r | r |  | 0 | 0 | 0 | 0 | 0 | r | r | r | r |

**Table A8. Expected genetic values of *Z_4i_* under the F_∞_ and F_2_ metric model in RIL-based aTTC design.**

| Genotype of  RIL | Frequency | F_∞_ | | | | | | | | |  | F_2_ | | | | | | | | |
| --- | --- | --- | --- | --- | --- | --- | --- | --- | --- | --- | --- | --- | --- | --- | --- | --- | --- | --- | --- | --- |
|  |  | $\mu$ | $a_{1}$ | $d_{1}$ | $a_{2}$ | $d_{2}$ |  |  |  |  |  | $\mu$ | $a_{1}$ | $d_{1}$ | $a_{2}$ | $d_{2}$ |  |  |  |  |
| $\frac{\begin{matrix} A & B \end{matrix}}{\begin{matrix} A & B \end{matrix}}$ | (1-*R*)/2 | 1 | 0 | 1 | 0 | 1 | 0 | 0 | 0 | 1 |  | 1 | 0 | 1/2 | 0 | 1/2 | 0 | 0 | 0 | 1/4 |
| $\frac{\begin{matrix} A & b \end{matrix}}{\begin{matrix} A & b \end{matrix}}$ | *R*/2 | 1 | 0 | 1 | 0 | 1 | 1 | 1 | -1 | 0 |  | 1 | 0 | 1/2 | 0 | 1/2 | 1 | 1 | -1 | -3/4 |
| $\frac{\begin{matrix} a & B \end{matrix}}{\begin{matrix} a & B \end{matrix}}$ | *R*/2 | 1 | 0 | 1 | 0 | 1 | 1 | -1 | 1 | 0 |  | 1 | 0 | 1/2 | 0 | 1/2 | 1 | -1 | 1 | -3/4 |
| $\frac{\begin{matrix} a & b \end{matrix}}{\begin{matrix} a & b \end{matrix}}$ | (1-*R*)/2 | 1 | 0 | 1 | 0 | 1 | 0 | 0 | 0 | 1 |  | 1 | 0 | 1/2 | 0 | 1/2 | 0 | 0 | 0 | 1/4 |

**Table A9. Expected genetic values of *Z_5i_* under the F_∞_ and F_2_ metric model in RIL-based aTTC design.**

| Genotype of  RIL | Frequency | F_∞_ | | | | | | | | |  | F_2_ | | | | | | | | |
| --- | --- | --- | --- | --- | --- | --- | --- | --- | --- | --- | --- | --- | --- | --- | --- | --- | --- | --- | --- | --- |
|  |  | $\mu$ | $a_{1}$ | $d_{1}$ | $a_{2}$ | $d_{2}$ |  |  |  |  |  | $\mu$ | $a_{1}$ | $d_{1}$ | $a_{2}$ | $d_{2}$ |  |  |  |  |
| $\frac{\begin{matrix} A & B \end{matrix}}{\begin{matrix} A & B \end{matrix}}$ | (1-*R*)/2 | 3 | 3/2 | 3/2 | 3/2 | 3/2 | (3-r)/2 | r/2 | r/2 | (3-r)/2 |  | 3 | 3/2 | 0 | 3/2 | 0 | (3-r)/2 | -(3-2r)/4 | -(3-2r)/4 | (3-2r)/4 |
| $\frac{\begin{matrix} A & b \end{matrix}}{\begin{matrix} A & b \end{matrix}}$ | *R*/2 | 3 | 3/2 | 3/2 | -3/2 | 3/2 | -r/2 | (3-r)/2 | -(3-r)/2 | r/2 |  | 3 | 3/2 | 0 | -3/2 | 0 | -r/2 | (3-2r)/4 | -(3-2r)/4 | -(3-2r)/4 |
| $\frac{\begin{matrix} a & B \end{matrix}}{\begin{matrix} a & B \end{matrix}}$ | *R*/2 | 3 | -3/2 | 3/2 | 3/2 | 3/2 | -r/2 | -(3-r)/2 | (3-r)/2 | r/2 |  | 3 | -3/2 | 0 | 3/2 | 0 | -r/2 | -(3-2r)/4 | (3-2r)/4 | -(3-2r)/4 |
| $\frac{\begin{matrix} a & b \end{matrix}}{\begin{matrix} a & b \end{matrix}}$ | (1-*R*)/2 | 3 | -3/2 | 3/2 | -3/2 | 3/2 | (3-r)/2 | -r/2 | -r/2 | (3-r)/2 |  | 3 | -3/2 | 0 | -3/2 | 0 | (3-r)/2 | (3-2r)/4 | (3-2r)/4 | (3-2r)/4 |

**Table A10. Expected genetic values of *Z_6i_* under the F_∞_ and F_2_ metric model in RIL-based aTTC design.**

| Genotype of  RIL | Frequency | F_∞_ | | | | | | | | |  | F_2_ | | | | | | | | |
| --- | --- | --- | --- | --- | --- | --- | --- | --- | --- | --- | --- | --- | --- | --- | --- | --- | --- | --- | --- | --- |
|  |  | $\mu$ | $a_{1}$ | $d_{1}$ | $a_{2}$ | $d_{2}$ |  |  |  |  |  | $\mu$ | $a_{1}$ | $d_{1}$ | $a_{2}$ | $d_{2}$ |  |  |  |  |
| $\frac{\begin{matrix} A & B \end{matrix}}{\begin{matrix} A & B \end{matrix}}$ | (1-*R*)/2 | 1 | 0 | 1 | 0 | 1 | -r | r | r | 1-r |  | 1 | 0 | 0 | 0 | 0 | -r | -(1-2r)/2 | -(1-2r)/2 | (1-2r)/2 |
| $\frac{\begin{matrix} A & b \end{matrix}}{\begin{matrix} A & b \end{matrix}}$ | *R*/2 | 1 | 0 | 1 | 0 | 1 | 1-r | 1-r | -(1-r) | r |  | 1 | 0 | 0 | 0 | 0 | 1-r | (1-2r)/2 | -(1-2r)/2 | -(1-2r)/2 |
| $\frac{\begin{matrix} a & B \end{matrix}}{\begin{matrix} a & B \end{matrix}}$ | *R*/2 | 1 | 0 | 1 | 0 | 1 | 1-r | -(1-r) | 1-r | r |  | 1 | 0 | 0 | 0 | 0 | 1-r | -(1-2r)/2 | (1-2r)/2 | -(1-2r)/2 |
| $\frac{\begin{matrix} a & b \end{matrix}}{\begin{matrix} a & b \end{matrix}}$ | (1-*R*)/2 | 1 | 0 | 1 | 0 | 1 | -r | -r | -r | 1-r |  | 1 | 0 | 0 | 0 | 0 | -r | (1-2r)/2 | (1-2r)/2 | (1-2r)/2 |
