# Supplementary material for: Genetic dissection of main and epistatic effects of QTL based on augmented triple test cross design
Source: PLoS One. 2017 Dec 14;12(12):e0189054. doi: 10.1371/journal.pone.0189054 (PMC5730204; doi:10.1371/journal.pone.0189054)
Supplement: S2 Supporting Information — (DOCX) [file pone.0189054.s002.docx]

**Expected genetic values of *Z_1i_*, *Z_2i_*, *Z_3i_*, *Z_4i_*, *Z_5i_* and *Z_6i_* under both the F_∞_ and F_2_ metric models in F_2_-based aTTC design**

We assume that a quantitative trait is controlled by two QTL, *A* and *B*, each with two alleles (*A*, *a*; *B*, *b*). The recombination fraction between A and B was *r*. The genotypes of the two inbred lines (*P_1_* and *P_2_*) are *AABB* and *aabb*, respectively, and their *F_1_* are *AaBb*. The *F_1_* produces four types of gametes *AB*, *Ab*, *aB* and *ab* with frequencies (1-r)/2, r/2, r/2 and (1-r)/2. The gametes from *F_1_* are combined to produce *F_2_* population with 9 type genotypes. Note that the two double heterozygotes $AB/ab$ and $Ab/aB$, with different gametes constitutions under linkage disequilibrium, should be distinguished. In aTTC design, individuals from the *F_2_* population are crossed to *P_1_* (with gamete *AB*) and *P_2_* (with gamete *ab*) and *F_1_* to produce *L_1_* and *L_2_* and *L_3_* families, at the same time, the *n* individuals in base population were self-mated to get *L_4_*.The expected genetic values of *L_1i_*, *L_2i_*, *L_3i_* and *L_4i_* under the F_∞_ and F_2_ metric model in F_2_-based aTTC design are presented in Table B1, Table B2, Table B3 and Table B4, respectively.

**Table B1. Expected genetic values of *L_1i_* under the F_∞_ and F_2_ metric model in F_2_-based aTTC design.**

| Genotype of  F_2_ plant | F_2_ metric model | | | | | | | | |  | F_∞_ metric model | | | | | | | | |
| --- | --- | --- | --- | --- | --- | --- | --- | --- | --- | --- | --- | --- | --- | --- | --- | --- | --- | --- | --- |
|  | $\mu$ | $a_{1}$ | $d_{1}$ | $a_{2}$ | $d_{2}$ | $i_{a_{1}a_{2}}$ | $i_{a_{1}d_{2}}$ | $i_{d_{1}a_{2}}$ | $i_{d_{1}d_{2}}$ |  | $\mu$ | $a_{1}$ | $d_{1}$ | $a_{2}$ | $d_{2}$ | $i_{a_{1}a_{2}}$ | $i_{a_{1}d_{2}}$ | $i_{d_{1}a_{2}}$ | $i_{d_{1}d_{2}}$ |
| $\frac{\begin{matrix} A & B \end{matrix}}{\begin{matrix} A & B \end{matrix}}$ | $1$ | $1$ | $-\frac{1}{2}$ | $1$ | $-\frac{1}{2}$ | $1$ | $-\frac{1}{2}$ | $-\frac{1}{2}$ | $\frac{1}{4}$ |  | $1$ | $1$ | $0$ | $1$ | $0$ | $1$ | $0$ | $0$ | $0$ |
| $\frac{\begin{matrix} A & B \end{matrix}}{\begin{matrix} A & b \end{matrix}}$ | $1$ | $1$ | $-\frac{1}{2}$ | $\frac{1}{2}$ | $0$ | $\frac{1}{2}$ | $0$ | $-\frac{1}{4}$ | $0$ |  | $1$ | $1$ | $0$ | $\frac{1}{2}$ | $\frac{1}{2}$ | $\frac{1}{2}$ | $\frac{1}{2}$ | $0$ | $0$ |
| $\frac{\begin{matrix} A & b \end{matrix}}{\begin{matrix} A & b \end{matrix}}$ | $1$ | $1$ | $-\frac{1}{2}$ | $0$ | $\frac{1}{2}$ | $0$ | $\frac{1}{2}$ | $0$ | $-\frac{1}{4}$ |  | $1$ | $1$ | $0$ | $0$ | $1$ | $0$ | $1$ | $0$ | $0$ |
| $\frac{\begin{matrix} A & B \end{matrix}}{\begin{matrix} a & B \end{matrix}}$ | $1$ | $\frac{1}{2}$ | $0$ | $1$ | $-\frac{1}{2}$ | $\frac{1}{2}$ | $-\frac{1}{4}$ | $0$ | $0$ |  | $1$ | $\frac{1}{2}$ | $\frac{1}{2}$ | $1$ | $0$ | $\frac{1}{2}$ | $0$ | $\frac{1}{2}$ | $0$ |
| $\frac{\begin{matrix} A & B \end{matrix}}{\begin{matrix} a & b \end{matrix}}$ | $1$ | $\frac{1}{2}$ | $0$ | $\frac{1}{2}$ | $0$ | $\frac{1}{4}$ | $0$ | $0$ | $0$ |  | $1$ | $\frac{1}{2}$ | $\frac{1}{2}$ | $\frac{1}{2}$ | $\frac{1}{2}$ | $\frac{1}{4}$ | $\frac{1}{4}$ | $\frac{1}{4}$ | $\frac{1}{4}$ |
| $\frac{\begin{matrix} A & b \end{matrix}}{\begin{matrix} a & b \end{matrix}}$ | $1$ | $\frac{1}{2}$ | $0$ | $0$ | $\frac{1}{2}$ | $0$ | $\frac{1}{4}$ | $0$ | $0$ |  | $1$ | $\frac{1}{2}$ | $\frac{1}{2}$ | $0$ | $1$ | $0$ | $\frac{1}{2}$ | $0$ | $\frac{1}{2}$ |
| $\frac{\begin{matrix} a & B \end{matrix}}{\begin{matrix} a & B \end{matrix}}$ | $1$ | $0$ | $\frac{1}{2}$ | $1$ | $-\frac{1}{2}$ | $0$ | $0$ | $\frac{1}{2}$ | $-\frac{1}{4}$ |  | $1$ | $0$ | $1$ | $1$ | $0$ | $0$ | $0$ | $1$ | $0$ |
| $\frac{\begin{matrix} a & B \end{matrix}}{\begin{matrix} a & b \end{matrix}}$ | $1$ | $0$ | $\frac{1}{2}$ | $\frac{1}{2}$ | $0$ | $0$ | $0$ | $\frac{1}{4}$ | $0$ |  | $1$ | $0$ | $1$ | $\frac{1}{2}$ | $\frac{1}{2}$ | $0$ | $0$ | $\frac{1}{2}$ | $\frac{1}{2}$ |
| $\frac{\begin{matrix} a & b \end{matrix}}{\begin{matrix} a & b \end{matrix}}$ | $1$ | $0$ | $\frac{1}{2}$ | $0$ | $\frac{1}{2}$ | $0$ | $0$ | $0$ | $\frac{1}{4}$ |  | $1$ | $0$ | $1$ | $0$ | $1$ | $0$ | $0$ | $0$ | $1$ |

**Table B2. Expected genetic values of *L_2i_* under the F_∞_ and F_2_ metric model in F_2_-based aTTC design.**

| Genotype of  F_2_ plant | F_2_ metric model | | | | | | | | |  | F_∞_ metric model | | | | | | | | |
| --- | --- | --- | --- | --- | --- | --- | --- | --- | --- | --- | --- | --- | --- | --- | --- | --- | --- | --- | --- |
|  | $\mu$ | $a_{1}$ | $d_{1}$ | $a_{2}$ | $d_{2}$ | $i_{a_{1}a_{2}}$ | $i_{a_{1}d_{2}}$ | $i_{d_{1}a_{2}}$ | $i_{d_{1}d_{2}}$ |  | $\mu$ | $a_{1}$ | $d_{1}$ | $a_{2}$ | $d_{2}$ | $i_{a_{1}a_{2}}$ | $i_{a_{1}d_{2}}$ | $i_{d_{1}a_{2}}$ | $i_{d_{1}d_{2}}$ |
| $\frac{\begin{matrix} A & B \end{matrix}}{\begin{matrix} A & B \end{matrix}}$ | $1$ | $0$ | $\frac{1}{2}$ | $0$ | $\frac{1}{2}$ | $0$ | $0$ | $0$ | $\frac{1}{4}$ |  | $1$ | $0$ | $1$ | $0$ | $1$ | $0$ | $0$ | $0$ | $1$ |
| $\frac{\begin{matrix} A & B \end{matrix}}{\begin{matrix} A & b \end{matrix}}$ | $1$ | $0$ | $\frac{1}{2}$ | $-\frac{1}{2}$ | $0$ | $0$ | $0$ | $-\frac{1}{4}$ | $0$ |  | $1$ | $0$ | $1$ | $-\frac{1}{2}$ | $\frac{1}{2}$ | $0$ | $0$ | $-\frac{1}{2}$ | $\frac{1}{2}$ |
| $\frac{\begin{matrix} A & b \end{matrix}}{\begin{matrix} A & b \end{matrix}}$ | $1$ | $0$ | $\frac{1}{2}$ | $-1$ | $-\frac{1}{2}$ | $0$ | $0$ | $-\frac{1}{2}$ | $-\frac{1}{4}$ |  | $1$ | $0$ | $1$ | $-1$ | $0$ | $0$ | $0$ | $-1$ | $0$ |
| $\frac{\begin{matrix} A & B \end{matrix}}{\begin{matrix} a & B \end{matrix}}$ | $1$ | $-\frac{1}{2}$ | $0$ | $0$ | $\frac{1}{2}$ | $0$ | $-\frac{1}{4}$ | $0$ | $0$ |  | $1$ | $-\frac{1}{2}$ | $\frac{1}{2}$ | $0$ | $1$ | $0$ | $-\frac{1}{2}$ | $0$ | $\frac{1}{2}$ |
| $\frac{\begin{matrix} A & B \end{matrix}}{\begin{matrix} a & b \end{matrix}}$ | $1$ | $-\frac{1}{2}$ | $0$ | $-\frac{1}{2}$ | $0$ | $\frac{1}{4}$ | $0$ | $0$ | $0$ |  | $1$ | $-\frac{1}{2}$ | $\frac{1}{2}$ | $-\frac{1}{2}$ | $\frac{1}{2}$ | $\frac{1}{4}$ | $-\frac{1}{4}$ | $-\frac{1}{4}$ | $\frac{1}{4}$ |
| $\frac{\begin{matrix} A & b \end{matrix}}{\begin{matrix} a & b \end{matrix}}$ | $1$ | $-\frac{1}{2}$ | $0$ | $-1$ | $-\frac{1}{2}$ | $\frac{1}{2}$ | $\frac{1}{4}$ | $0$ | $0$ |  | $1$ | $-\frac{1}{2}$ | $\frac{1}{2}$ | $-1$ | $0$ | $\frac{1}{2}$ | $0$ | $-\frac{1}{2}$ | $0$ |
| $\frac{\begin{matrix} a & B \end{matrix}}{\begin{matrix} a & B \end{matrix}}$ | $1$ | $-1$ | $-\frac{1}{2}$ | $0$ | $\frac{1}{2}$ | $0$ | $-\frac{1}{2}$ | $0$ | $-\frac{1}{4}$ |  | $1$ | $-1$ | $0$ | $0$ | $1$ | $0$ | $-1$ | $0$ | $0$ |
| $\frac{\begin{matrix} a & B \end{matrix}}{\begin{matrix} a & b \end{matrix}}$ | $1$ | $-1$ | $-\frac{1}{2}$ | $-\frac{1}{2}$ | $0$ | $\frac{1}{2}$ | $0$ | $\frac{1}{4}$ | $0$ |  | $1$ | $-1$ | $0$ | $-\frac{1}{2}$ | $\frac{1}{2}$ | $\frac{1}{2}$ | $-\frac{1}{2}$ | $0$ | $0$ |
| $\frac{\begin{matrix} a & b \end{matrix}}{\begin{matrix} a & b \end{matrix}}$ | $1$ | $-1$ | $-\frac{1}{2}$ | $-1$ | $-\frac{1}{2}$ | $1$ | $\frac{1}{2}$ | $\frac{1}{2}$ | $\frac{1}{4}$ |  | $1$ | $-1$ | $0$ | $-1$ | $0$ | $1$ | $0$ | $0$ | $0$ |

**Table B3. Expected genetic values of *L_3i_* under the F_∞_ and F_2_ metric model in F_2_-based aTTC design.**

| Genotype of  F_2_ plant | F_2_ metric model | | | | | | | | |  | F_∞_ metric model | | | | | | | | |
| --- | --- | --- | --- | --- | --- | --- | --- | --- | --- | --- | --- | --- | --- | --- | --- | --- | --- | --- | --- |
|  | $\mu$ | $a_{1}$ | $d_{1}$ | $a_{2}$ | $d_{2}$ | $i_{a_{1}a_{2}}$ | $i_{a_{1}d_{2}}$ | $i_{d_{1}a_{2}}$ | $i_{d_{1}d_{2}}$ |  | $\mu$ | $a_{1}$ | $d_{1}$ | $a_{2}$ | $d_{2}$ | $i_{a_{1}a_{2}}$ | $i_{a_{1}d_{2}}$ | $i_{d_{1}a_{2}}$ | $i_{d_{1}d_{2}}$ |
| $\frac{\begin{matrix} A & B \end{matrix}}{\begin{matrix} A & B \end{matrix}}$ | $1$ | $\frac{1}{2}$ | $0$ | $\frac{1}{2}$ | $0$ | $\frac{1}{2}(1-r)$ | $-\frac{1}{4}(1-2r)$ | $-\frac{1}{4}(1-2r)$ | $\frac{1}{4}(1-2r)$ |  | $1$ | $\frac{1}{2}$ | $\frac{1}{2}$ | $\frac{1}{2}$ | $\frac{1}{2}$ | $\frac{1}{2}(1-r)$ | $\frac{1}{2}r$ | $\frac{1}{2}r$ | $\frac{1}{2}(1-r)$ |
| $\frac{\begin{matrix} A & B \end{matrix}}{\begin{matrix} A & b \end{matrix}}$ | $1$ | $\frac{1}{2}$ | $0$ | $0$ | $0$ | $\frac{1}{4}(1-2r)$ | $0$ | $-\frac{1}{4}(1-2r)$ | $0$ |  | $1$ | $\frac{1}{2}$ | $\frac{1}{2}$ | $0$ | $\frac{1}{2}$ | $\frac{1}{4}(1-2r)$ | $\frac{1}{4}$ | $-\frac{1}{4}(1-2r)$ | $\frac{1}{4}$ |
| $\frac{\begin{matrix} A & b \end{matrix}}{\begin{matrix} A & b \end{matrix}}$ | $1$ | $\frac{1}{2}$ | $0$ | $-\frac{1}{2}$ | $0$ | $-\frac{1}{2}r$ | $\frac{1}{4}(1-2r)$ | $-\frac{1}{4}(1-2r)$ | $-\frac{1}{4}(1-2r)$ |  | $1$ | $\frac{1}{2}$ | $\frac{1}{2}$ | $-\frac{1}{2}$ | $\frac{1}{2}$ | $-\frac{1}{2}r$ | $\frac{1}{2}(1-r)$ | $-\frac{1}{2}(1-r)$ | $\frac{1}{2}r$ |
| $\frac{\begin{matrix} A & B \end{matrix}}{\begin{matrix} a & B \end{matrix}}$ | $1$ | $0$ | $0$ | $\frac{1}{2}$ | $0$ | $\frac{1}{4}(1-2r)$ | $-\frac{1}{4}(1-2r)$ | $0$ | $0$ |  | $1$ | $0$ | $\frac{1}{2}$ | $\frac{1}{2}$ | $\frac{1}{2}$ | $\frac{1}{4}(1-2r)$ | $-\frac{1}{4}(1-2r)$ | $\frac{1}{4}$ | $\frac{1}{4}$ |
| $\frac{\begin{matrix} A & B \end{matrix}}{\begin{matrix} a & b \end{matrix}}$ | $1$ | $0$ | $0$ | $0$ | $0$ | $\frac{1}{4}(1-2r)$ | $0$ | $0$ | $0$ |  | $1$ | $0$ | $\frac{1}{2}$ | $0$ | $\frac{1}{2}$ | $\frac{1}{4}(1-2r)$ | $0$ | $0$ | $\frac{1}{4}$ |
| $\frac{\begin{matrix} A & b \end{matrix}}{\begin{matrix} a & b \end{matrix}}$ | $1$ | $0$ | $0$ | $-\frac{1}{2}$ | $0$ | $\frac{1}{4}(1-2r)$ | $\frac{1}{4}(1-2r)$ | $0$ | $0$ |  | $1$ | $0$ | $\frac{1}{2}$ | $-\frac{1}{2}$ | $\frac{1}{2}$ | $\frac{1}{4}(1-2r)$ | $\frac{1}{4}(1-2r)$ | $-\frac{1}{4}$ | $\frac{1}{4}$ |
| $\frac{\begin{matrix} a & B \end{matrix}}{\begin{matrix} a & B \end{matrix}}$ | $1$ | $-\frac{1}{2}$ | $0$ | $\frac{1}{2}$ | $0$ | $-\frac{1}{2}r$ | $-\frac{1}{4}(1-2r)$ | $\frac{1}{4}(1-2r)$ | $-\frac{1}{4}(1-2r)$ |  | $1$ | $-\frac{1}{2}$ | $\frac{1}{2}$ | $\frac{1}{2}$ | $\frac{1}{2}$ | $-\frac{1}{2}r$ | $-\frac{1}{2}(1-r)$ | $\frac{1}{2}(1-r)$ | $\frac{1}{2}r$ |
| $\frac{\begin{matrix} a & B \end{matrix}}{\begin{matrix} a & b \end{matrix}}$ | $1$ | $-\frac{1}{2}$ | $0$ | $0$ | $0$ | $\frac{1}{4}(1-2r)$ | $0$ | $\frac{1}{4}(1-2r)$ | $0$ |  | $1$ | $-\frac{1}{2}$ | $\frac{1}{2}$ | $0$ | $\frac{1}{2}$ | $\frac{1}{4}(1-2r)$ | $-\frac{1}{4}$ | $\frac{1}{4}(1-2r)$ | $\frac{1}{4}$ |
| $\frac{\begin{matrix} a & b \end{matrix}}{\begin{matrix} a & b \end{matrix}}$ | $1$ | $-\frac{1}{2}$ | $0$ | $-\frac{1}{2}$ | $0$ | $\frac{1}{2}(1-r)$ | $\frac{1}{4}(1-2r)$ | $\frac{1}{4}(1-2r)$ | $\frac{1}{4}(1-2r)$ |  | $1$ | $-\frac{1}{2}$ | $\frac{1}{2}$ | $-\frac{1}{2}$ | $\frac{1}{2}$ | $\frac{1}{2}(1-r)$ | $-\frac{1}{2}r$ | $-\frac{1}{2}r$ | $\frac{1}{2}(1-r)$ |

**Table B4. Expected genetic values of *L_4i_* under the F_∞_ and F_2_ metric model in F_2_-based aTTC design.**

| Genotype of  F_2_ plant | F_2_ metric model | | | | | | | | |  | F_∞_ metric model | | | | | | | | |
| --- | --- | --- | --- | --- | --- | --- | --- | --- | --- | --- | --- | --- | --- | --- | --- | --- | --- | --- | --- |
|  | $\mu$ | $a_{1}$ | $d_{1}$ | $a_{2}$ | $d_{2}$ | $i_{a_{1}a_{2}}$ | $i_{a_{1}d_{2}}$ | $i_{d_{1}a_{2}}$ | $i_{d_{1}d_{2}}$ |  | $\mu$ | $a_{1}$ | $d_{1}$ | $a_{2}$ | $d_{2}$ | $i_{a_{1}a_{2}}$ | $i_{a_{1}d_{2}}$ | $i_{d_{1}a_{2}}$ | $i_{d_{1}d_{2}}$ |
| $\frac{\begin{matrix} A & B \end{matrix}}{\begin{matrix} A & B \end{matrix}}$ | $1$ | $1$ | $-\frac{1}{2}$ | $1$ | $-\frac{1}{2}$ | $1$ | $-\frac{1}{2}$ | $-\frac{1}{2}$ | $\frac{1}{4}$ |  | $1$ | $1$ | $0$ | $1$ | $0$ | $1$ | $0$ | $0$ | $0$ |
| $\frac{\begin{matrix} A & B \end{matrix}}{\begin{matrix} A & b \end{matrix}}$ | $1$ | $1$ | $-\frac{1}{2}$ | $0$ | $\frac{1}{4}$ | $0$ | $\frac{1}{4}$ | $0$ | $-\frac{1}{8}$ |  | $1$ | $1$ | $0$ | $0$ | $\frac{1}{2}$ | $0$ | $\frac{1}{2}$ | $0$ | $0$ |
| $\frac{\begin{matrix} A & b \end{matrix}}{\begin{matrix} A & b \end{matrix}}$ | $1$ | $1$ | $-\frac{1}{2}$ | $-1$ | $-\frac{1}{2}$ | $-1$ | $-\frac{1}{2}$ | $\frac{1}{2}$ | $\frac{1}{4}$ |  | $1$ | $1$ | $0$ | $-1$ | $0$ | $-1$ | $0$ | $0$ | $0$ |
| $\frac{\begin{matrix} A & B \end{matrix}}{\begin{matrix} a & B \end{matrix}}$ | $1$ | $0$ | $\frac{1}{4}$ | $1$ | $-\frac{1}{2}$ | $1$ | $0$ | $\frac{1}{4}$ | $-\frac{1}{8}$ |  | $1$ | $0$ | $\frac{1}{2}$ | $1$ | $0$ | $0$ | $0$ | $\frac{1}{2}$ | $0$ |
| $\frac{\begin{matrix} A & B \end{matrix}}{\begin{matrix} a & b \end{matrix}}$ | $1$ | $0$ | $\frac{3}{4}$ | $0$ | $-\frac{3}{4}$ | $0$ | $0$ | $0$ | $-\frac{9}{16}$ |  | $1$ | $0$ | $\frac{1}{2}$ | $0$ | $\frac{1}{2}$ | $0$ | $0$ | $0$ | $\frac{1}{4}$ |
| $\frac{\begin{matrix} A & b \end{matrix}}{\begin{matrix} a & b \end{matrix}}$ | $1$ | $0$ | $\frac{1}{4}$ | $-1$ | $-\frac{1}{2}$ | $0$ | $0$ | $-\frac{1}{4}$ | $-\frac{1}{8}$ |  | $1$ | $0$ | $\frac{1}{2}$ | $-1$ | $0$ | $0$ | $0$ | $-\frac{1}{2}$ | $0$ |
| $\frac{\begin{matrix} a & B \end{matrix}}{\begin{matrix} a & B \end{matrix}}$ | $1$ | $-1$ | $-\frac{1}{2}$ | $1$ | $-\frac{1}{2}$ | $-1$ | $\frac{1}{2}$ | $-\frac{1}{2}$ | $\frac{1}{4}$ |  | $1$ | $-1$ | $0$ | $1$ | $0$ | $-1$ | $0$ | $0$ | $0$ |
| $\frac{\begin{matrix} a & B \end{matrix}}{\begin{matrix} a & b \end{matrix}}$ | $1$ | $-1$ | $-\frac{1}{2}$ | $0$ | $\frac{1}{4}$ | $0$ | $-\frac{1}{4}$ | $0$ | $-\frac{1}{8}$ |  | $1$ | $-1$ | $0$ | $0$ | $\frac{1}{2}$ | $0$ | $-\frac{1}{2}$ | $0$ | $0$ |
| $\frac{\begin{matrix} a & b \end{matrix}}{\begin{matrix} a & b \end{matrix}}$ | $1$ | $-1$ | $-\frac{1}{2}$ | $-1$ | $-\frac{1}{2}$ | $1$ | $\frac{1}{2}$ | $\frac{1}{2}$ | $\frac{1}{4}$ |  | $1$ | $-1$ | $0$ | $-1$ | $0$ | $1$ | $0$ | $0$ | $0$ |

where $\mu$ is model mean (mean genotypic values of four homozygotes, *AABB*, *AAbb*, *aaBB* and *aabb*); $a_{1}$ is the additive effect of QTL *A*, i.e. average substitution effect of *a* (in *aaBB* and *aabb*) by *A*; $d_{1}$ is the dominance effect of QTL *A*, i.e. the departure in genotypic value of the heterozygote mean of *Aa* (*AaBB* and *Aabb*) from the midpoint between the two homozygote means of *AA* (*AABB* and *AAbb*) and *aa* (*aaBB* and *aabb*); $a_{2}$ and $d_{2}$ can be similarly defined;, , and are additive × additive, additive × dominance, dominance × additive and dominance × dominance epistatic effects between QTL *A* and *B*.

The expected genetic values of *Z_1i_*, *Z_2i_*, *Z_3i_*, *Z_4i_*, *Z_5i_* and *Z_6i_* under both the F_∞_ and F_2_ metric models in RIL-based aTTC design are presented in Table B5, Table B6, Table B7, Table B8, Table B9 and Table B10, respectively.

**Table B5. Expected genetic values of *Z_1i_* under the F_∞_ and F_2_ metric model in F_2_-based aTTC design.**

| Genotype of  F_2_ plant | F_2_ metric model | | | | | | | | |  | F_∞_ metric model | | | | | | | | |
| --- | --- | --- | --- | --- | --- | --- | --- | --- | --- | --- | --- | --- | --- | --- | --- | --- | --- | --- | --- |
|  | $\mu$ | $a_{1}$ | $d_{1}$ | $a_{2}$ | $d_{2}$ | $i_{a_{1}a_{2}}$ | $i_{a_{1}d_{2}}$ | $i_{d_{1}a_{2}}$ | $i_{d_{1}d_{2}}$ |  | $\mu$ | $a_{1}$ | $d_{1}$ | $a_{2}$ | $d_{2}$ | $i_{a_{1}a_{2}}$ | $i_{a_{1}d_{2}}$ | $i_{d_{1}a_{2}}$ | $i_{d_{1}d_{2}}$ |
| $\frac{\begin{matrix} A & B \end{matrix}}{\begin{matrix} A & B \end{matrix}}$ | $2$ | $1$ | $0$ | $1$ | $0$ | $1$ | $-\frac{1}{2}$ | $-\frac{1}{2}$ | $\frac{1}{2}$ |  | $2$ | $1$ | $1$ | $1$ | $1$ | $1$ | $0$ | $0$ | $1$ |
| $\frac{\begin{matrix} A & B \end{matrix}}{\begin{matrix} A & b \end{matrix}}$ | $2$ | $1$ | $0$ | $0$ | $0$ | $\frac{1}{2}$ | $0$ | $-\frac{1}{2}$ | $0$ |  | $2$ | $1$ | $1$ | $0$ | $1$ | $\frac{1}{2}$ | $\frac{1}{2}$ | $-\frac{1}{2}$ | $\frac{1}{2}$ |
| $\frac{\begin{matrix} A & b \end{matrix}}{\begin{matrix} A & b \end{matrix}}$ | $2$ | $1$ | $0$ | $-1$ | $0$ | $0$ | $\frac{1}{2}$ | $-\frac{1}{2}$ | $-\frac{1}{2}$ |  | $2$ | $1$ | $1$ | $-1$ | $1$ | $0$ | $1$ | $-1$ | $0$ |
| $\frac{\begin{matrix} A & B \end{matrix}}{\begin{matrix} a & B \end{matrix}}$ | $2$ | $0$ | $0$ | $1$ | $0$ | $\frac{1}{2}$ | $-\frac{1}{2}$ | $0$ | $0$ |  | $2$ | $0$ | $1$ | $1$ | $1$ | $\frac{1}{2}$ | $-\frac{1}{2}$ | $\frac{1}{2}$ | $\frac{1}{2}$ |
| $\frac{\begin{matrix} A & B \end{matrix}}{\begin{matrix} a & b \end{matrix}}$ | $2$ | $0$ | $0$ | $0$ | $0$ | $\frac{1}{2}$ | $0$ | $0$ | $0$ |  | $2$ | $0$ | $1$ | $0$ | $1$ | $\frac{1}{2}$ | $0$ | $0$ | $\frac{1}{2}$ |
| $\frac{\begin{matrix} A & b \end{matrix}}{\begin{matrix} a & b \end{matrix}}$ | $2$ | $0$ | $0$ | $-1$ | $0$ | $\frac{1}{2}$ | $\frac{1}{2}$ | $0$ | $0$ |  | $2$ | $0$ | $1$ | $-1$ | $1$ | $\frac{1}{2}$ | $\frac{1}{2}$ | $-\frac{1}{2}$ | $\frac{1}{2}$ |
| $\frac{\begin{matrix} a & B \end{matrix}}{\begin{matrix} a & B \end{matrix}}$ | $2$ | $-1$ | $0$ | $1$ | $0$ | $0$ | $-\frac{1}{2}$ | $\frac{1}{2}$ | $-\frac{1}{2}$ |  | $2$ | $-1$ | $1$ | $1$ | $1$ | $0$ | $-1$ | $1$ | $0$ |
| $\frac{\begin{matrix} a & B \end{matrix}}{\begin{matrix} a & b \end{matrix}}$ | $2$ | $-1$ | $0$ | $0$ | $0$ | $\frac{1}{2}$ | $0$ | $\frac{1}{2}$ | $0$ |  | $2$ | $-1$ | $1$ | $0$ | $1$ | $\frac{1}{2}$ | $-\frac{1}{2}$ | $\frac{1}{2}$ | $\frac{1}{2}$ |
| $\frac{\begin{matrix} a & b \end{matrix}}{\begin{matrix} a & b \end{matrix}}$ | $2$ | $-1$ | $0$ | $-1$ | $0$ | $1$ | $\frac{1}{2}$ | $\frac{1}{2}$ | $\frac{1}{2}$ |  | $2$ | $-1$ | $1$ | $-1$ | $1$ | $1$ | $0$ | $0$ | $1$ |

**Table B6. Expected genetic values of *Z_2i_* under the F_∞_ and F_2_ metric model in F_2_-based aTTC design.**

| Genotype of  F_2_ plant | F_2_ metric model | | | | | | | | |  | F_∞_ metric model | | | | | | | | |
| --- | --- | --- | --- | --- | --- | --- | --- | --- | --- | --- | --- | --- | --- | --- | --- | --- | --- | --- | --- |
|  | $\mu$ | $a_{1}$ | $d_{1}$ | $a_{2}$ | $d_{2}$ | $i_{a_{1}a_{2}}$ | $i_{a_{1}d_{2}}$ | $i_{d_{1}a_{2}}$ | $i_{d_{1}d_{2}}$ |  | $\mu$ | $a_{1}$ | $d_{1}$ | $a_{2}$ | $d_{2}$ | $i_{a_{1}a_{2}}$ | $i_{a_{1}d_{2}}$ | $i_{d_{1}a_{2}}$ | $i_{d_{1}d_{2}}$ |
| $\frac{\begin{matrix} A & B \end{matrix}}{\begin{matrix} A & B \end{matrix}}$ | $0$ | $1$ | $-1$ | $1$ | $-1$ | $1$ | $-\frac{1}{2}$ | $-\frac{1}{2}$ | $0$ |  | $0$ | $1$ | $-1$ | $1$ | $-1$ | $1$ | $0$ | $0$ | $-1$ |
| $\frac{\begin{matrix} A & B \end{matrix}}{\begin{matrix} A & b \end{matrix}}$ | $0$ | $1$ | $-1$ | $1$ | $0$ | $\frac{1}{2}$ | $0$ | $0$ | $0$ |  | $0$ | $1$ | $-1$ | $1$ | $0$ | $\frac{1}{2}$ | $\frac{1}{2}$ | $\frac{1}{2}$ | $-\frac{1}{2}$ |
| $\frac{\begin{matrix} A & b \end{matrix}}{\begin{matrix} A & b \end{matrix}}$ | $0$ | $1$ | $-1$ | $1$ | $1$ | $0$ | $\frac{1}{2}$ | $\frac{1}{2}$ | $0$ |  | $0$ | $1$ | $-1$ | $1$ | $1$ | $0$ | $1$ | $1$ | $0$ |
| $\frac{\begin{matrix} A & B \end{matrix}}{\begin{matrix} a & B \end{matrix}}$ | $0$ | $1$ | $0$ | $1$ | $-1$ | $\frac{1}{2}$ | $0$ | $0$ | $0$ |  | $0$ | $1$ | $0$ | $1$ | $-1$ | $\frac{1}{2}$ | $\frac{1}{2}$ | $\frac{1}{2}$ | $-\frac{1}{2}$ |
| $\frac{\begin{matrix} A & B \end{matrix}}{\begin{matrix} a & b \end{matrix}}$ | $0$ | $1$ | $0$ | $1$ | $0$ | $0$ | $0$ | $0$ | $0$ |  | $0$ | $1$ | $0$ | $1$ | $0$ | $0$ | $\frac{1}{2}$ | $\frac{1}{2}$ | $0$ |
| $\frac{\begin{matrix} A & b \end{matrix}}{\begin{matrix} a & b \end{matrix}}$ | $0$ | $1$ | $0$ | $1$ | $1$ | $-\frac{1}{2}$ | $0$ | $0$ | $0$ |  | $0$ | $1$ | $0$ | $1$ | $1$ | $-\frac{1}{2}$ | $\frac{1}{2}$ | $\frac{1}{2}$ | $\frac{1}{2}$ |
| $\frac{\begin{matrix} a & B \end{matrix}}{\begin{matrix} a & B \end{matrix}}$ | $0$ | $1$ | $1$ | $1$ | $-1$ | $0$ | $\frac{1}{2}$ | $\frac{1}{2}$ | $0$ |  | $0$ | $1$ | $1$ | $1$ | $-1$ | $0$ | $1$ | $1$ | $0$ |
| $\frac{\begin{matrix} a & B \end{matrix}}{\begin{matrix} a & b \end{matrix}}$ | $0$ | $1$ | $1$ | $1$ | $0$ | $-\frac{1}{2}$ | $0$ | $0$ | $0$ |  | $0$ | $1$ | $1$ | $1$ | $0$ | $-\frac{1}{2}$ | $\frac{1}{2}$ | $\frac{1}{2}$ | $\frac{1}{2}$ |
| $\frac{\begin{matrix} a & b \end{matrix}}{\begin{matrix} a & b \end{matrix}}$ | $0$ | $1$ | $1$ | $1$ | $1$ | $-1$ | $-\frac{1}{2}$ | $-\frac{1}{2}$ | $0$ |  | $0$ | $1$ | $1$ | $1$ | $1$ | $-1$ | $0$ | $0$ | $1$ |

**Table B7. Expected genetic values of *Z_3i_* under the F_∞_ and F_2_ metric model in F_2_-based aTTC design.**

| Genotype of  F_2_ plant | F_2_ metric model | | | | | | | | |  | F_∞_ metric model | | | | | | | | |
| --- | --- | --- | --- | --- | --- | --- | --- | --- | --- | --- | --- | --- | --- | --- | --- | --- | --- | --- | --- |
|  | $\mu$ | $a_{1}$ | $d_{1}$ | $a_{2}$ | $d_{2}$ | $i_{a_{1}a_{2}}$ | $i_{a_{1}d_{2}}$ | $i_{d_{1}a_{2}}$ | $i_{d_{1}d_{2}}$ |  | $\mu$ | $a_{1}$ | $d_{1}$ | $a_{2}$ | $d_{2}$ | $i_{a_{1}a_{2}}$ | $i_{a_{1}d_{2}}$ | $i_{d_{1}a_{2}}$ | $i_{d_{1}d_{2}}$ |
| $\frac{\begin{matrix} A & B \end{matrix}}{\begin{matrix} A & B \end{matrix}}$ | $0$ | $0$ | $0$ | $0$ | $0$ | $r$ | $-r$ | $-r$ | $r$ |  | $0$ | $0$ | $0$ | $0$ | $0$ | $r$ | $-r$ | $-r$ | $r$ |
| $\frac{\begin{matrix} A & B \end{matrix}}{\begin{matrix} A & b \end{matrix}}$ | $0$ | $0$ | $0$ | $0$ | $0$ | $r$ | $0$ | $-r$ | $0$ |  | $0$ | $0$ | $0$ | $0$ | $0$ | $r$ | $0$ | $-r$ | $0$ |
| $\frac{\begin{matrix} A & b \end{matrix}}{\begin{matrix} A & b \end{matrix}}$ | $0$ | $0$ | $0$ | $0$ | $0$ | $r$ | $r$ | $-r$ | $-r$ |  | $0$ | $0$ | $0$ | $0$ | $0$ | $r$ | $r$ | $-r$ | $-r$ |
| $\frac{\begin{matrix} A & B \end{matrix}}{\begin{matrix} a & B \end{matrix}}$ | $0$ | $0$ | $0$ | $0$ | $0$ | $r$ | $-r$ | $0$ | $0$ |  | $0$ | $0$ | $0$ | $0$ | $0$ | $r$ | $-r$ | $0$ | $0$ |
| $\frac{\begin{matrix} A & B \end{matrix}}{\begin{matrix} a & b \end{matrix}}$ | $0$ | $0$ | $0$ | $0$ | $0$ | $r$ | $0$ | $0$ | $0$ |  | $0$ | $0$ | $0$ | $0$ | $0$ | $r$ | $0$ | $0$ | $0$ |
| $\frac{\begin{matrix} A & b \end{matrix}}{\begin{matrix} a & b \end{matrix}}$ | $0$ | $0$ | $0$ | $0$ | $0$ | $r$ | $r$ | $0$ | $0$ |  | $0$ | $0$ | $0$ | $0$ | $0$ | $r$ | $r$ | $0$ | $0$ |
| $\frac{\begin{matrix} a & B \end{matrix}}{\begin{matrix} a & B \end{matrix}}$ | $0$ | $0$ | $0$ | $0$ | $0$ | $r$ | $-r$ | $r$ | $-r$ |  | $0$ | $0$ | $0$ | $0$ | $0$ | $r$ | $-r$ | $r$ | $-r$ |
| $\frac{\begin{matrix} a & B \end{matrix}}{\begin{matrix} a & b \end{matrix}}$ | $0$ | $0$ | $0$ | $0$ | $0$ | $r$ | $0$ | $r$ | $0$ |  | $0$ | $0$ | $0$ | $0$ | $0$ | $r$ | $0$ | $r$ | $0$ |
| $\frac{\begin{matrix} a & b \end{matrix}}{\begin{matrix} a & b \end{matrix}}$ | $0$ | $0$ | $0$ | $0$ | $0$ | $r$ | $r$ | $r$ | $r$ |  | $0$ | $0$ | $0$ | $0$ | $0$ | $r$ | $r$ | $r$ | $r$ |

**Table B8. Expected genetic values of *Z_4i_* under the F_∞_ and F_2_ metric model in F_2_-based aTTC design.**

| Genotype of  F_2_ plant | F_2_ metric model | | | | | | | | |  | F_∞_ metric model | | | | | | | | |
| --- | --- | --- | --- | --- | --- | --- | --- | --- | --- | --- | --- | --- | --- | --- | --- | --- | --- | --- | --- |
|  | $\mu$ | $a_{1}$ | $d_{1}$ | $a_{2}$ | $d_{2}$ | $i_{a_{1}a_{2}}$ | $i_{a_{1}d_{2}}$ | $i_{d_{1}a_{2}}$ | $i_{d_{1}d_{2}}$ |  | $\mu$ | $a_{1}$ | $d_{1}$ | $a_{2}$ | $d_{2}$ | $i_{a_{1}a_{2}}$ | $i_{a_{1}d_{2}}$ | $i_{d_{1}a_{2}}$ | $i_{d_{1}d_{2}}$ |
| $\frac{\begin{matrix} A & B \end{matrix}}{\begin{matrix} A & B \end{matrix}}$ | $1$ | $0$ | $\frac{1}{2}$ | $0$ | $\frac{1}{2}$ | $0$ | $0$ | $0$ | $\frac{1}{4}$ |  | $1$ | $0$ | $1$ | $0$ | $1$ | $0$ | $0$ | $0$ | $1$ |
| $\frac{\begin{matrix} A & B \end{matrix}}{\begin{matrix} A & b \end{matrix}}$ | $1$ | $0$ | $\frac{1}{2}$ | $0$ | $-\frac{1}{4}$ | $\frac{1}{2}$ | $-\frac{1}{4}$ | $-\frac{1}{2}$ | $\frac{1}{8}$ |  | $1$ | $0$ | $1$ | $0$ | $\frac{1}{2}$ | $\frac{1}{2}$ | $0$ | $-\frac{1}{2}$ | $\frac{1}{2}$ |
| $\frac{\begin{matrix} A & b \end{matrix}}{\begin{matrix} A & b \end{matrix}}$ | $1$ | $0$ | $\frac{1}{2}$ | $0$ | $\frac{1}{2}$ | $1$ | $1$ | $-1$ | $-\frac{3}{4}$ |  | $1$ | $0$ | $1$ | $0$ | $1$ | $1$ | $1$ | $-1$ | $0$ |
| $\frac{\begin{matrix} A & B \end{matrix}}{\begin{matrix} a & B \end{matrix}}$ | $1$ | $0$ | $-\frac{1}{4}$ | $0$ | $\frac{1}{2}$ | $-\frac{1}{2}$ | $-\frac{1}{2}$ | $-\frac{1}{4}$ | $\frac{1}{8}$ |  | $1$ | $0$ | $\frac{1}{2}$ | $0$ | $1$ | $\frac{1}{2}$ | $-\frac{1}{2}$ | $0$ | $\frac{1}{2}$ |
| $\frac{\begin{matrix} A & B \end{matrix}}{\begin{matrix} a & b \end{matrix}}$ | $1$ | $0$ | $-\frac{3}{4}$ | $0$ | $\frac{3}{4}$ | $\frac{1}{2}$ | $0$ | $0$ | $\frac{9}{16}$ |  | $1$ | $0$ | $\frac{1}{2}$ | $0$ | $\frac{1}{2}$ | $\frac{1}{2}$ | $0$ | $0$ | $\frac{1}{4}$ |
| $\frac{\begin{matrix} A & b \end{matrix}}{\begin{matrix} a & b \end{matrix}}$ | $1$ | $0$ | $-\frac{1}{4}$ | $0$ | $\frac{1}{2}$ | $\frac{1}{2}$ | $\frac{1}{2}$ | $\frac{1}{4}$ | $\frac{1}{8}$ |  | $1$ | $0$ | $\frac{1}{2}$ | $0$ | $1$ | $\frac{1}{2}$ | $\frac{1}{2}$ | $0$ | $\frac{1}{2}$ |
| $\frac{\begin{matrix} a & B \end{matrix}}{\begin{matrix} a & B \end{matrix}}$ | $1$ | $0$ | $\frac{1}{2}$ | $0$ | $\frac{1}{2}$ | $1$ | $-1$ | $1$ | $-\frac{3}{4}$ |  | $1$ | $0$ | $1$ | $0$ | $1$ | $1$ | $-1$ | $1$ | $0$ |
| $\frac{\begin{matrix} a & B \end{matrix}}{\begin{matrix} a & b \end{matrix}}$ | $1$ | $0$ | $\frac{1}{2}$ | $0$ | $-\frac{1}{4}$ | $\frac{1}{2}$ | $\frac{1}{4}$ | $\frac{1}{2}$ | $\frac{1}{8}$ |  | $1$ | $0$ | $1$ | $0$ | $\frac{1}{2}$ | $\frac{1}{2}$ | $0$ | $\frac{1}{2}$ | $\frac{1}{2}$ |
| $\frac{\begin{matrix} a & b \end{matrix}}{\begin{matrix} a & b \end{matrix}}$ | $1$ | $0$ | $\frac{1}{2}$ | $0$ | $\frac{1}{2}$ | $0$ | $0$ | $0$ | $\frac{1}{4}$ |  | $1$ | $0$ | $1$ | $0$ | $1$ | $0$ | $0$ | $0$ | $1$ |

**Table B9. Expected genetic values of *Z_5i_* under the F_∞_ and F_2_ metric model in F_2_-based aTTC design.**

| Genotype of  F_2_ plant | F_2_ metric model | | | | | | | | |  | F_∞_ metric model | | | | | | | | |
| --- | --- | --- | --- | --- | --- | --- | --- | --- | --- | --- | --- | --- | --- | --- | --- | --- | --- | --- | --- |
|  | $\mu$ | $a_{1}$ | $d_{1}$ | $a_{2}$ | $d_{2}$ | $i_{a_{1}a_{2}}$ | $i_{a_{1}d_{2}}$ | $i_{d_{1}a_{2}}$ | $i_{d_{1}d_{2}}$ |  | $\mu$ | $a_{1}$ | $d_{1}$ | $a_{2}$ | $d_{2}$ | $i_{a_{1}a_{2}}$ | $i_{a_{1}d_{2}}$ | $i_{d_{1}a_{2}}$ | $i_{d_{1}d_{2}}$ |
| $\frac{\begin{matrix} A & B \end{matrix}}{\begin{matrix} A & B \end{matrix}}$ | $3$ | $\frac{3}{2}$ | $0$ | $\frac{3}{2}$ | $0$ | $\frac{1}{2}(3-r)$ | $-\frac{1}{4}(3-2r)$ | $-\frac{1}{4}(3-2r)$ | $\frac{1}{4}(3-2r)$ |  | $3$ | $\frac{3}{2}$ | $\frac{3}{2}$ | $\frac{3}{2}$ | $\frac{3}{2}$ | $\frac{1}{2}(3-r)$ | $\frac{r}{2}$ | $\frac{r}{2}$ | $\frac{1}{2}(3-r)$ |
| $\frac{\begin{matrix} A & B \end{matrix}}{\begin{matrix} A & b \end{matrix}}$ | $3$ | $\frac{3}{2}$ | $0$ | $0$ | $0$ | $\frac{1}{4}(3-2r)$ | $0$ | $-\frac{1}{4}(3-2r)$ | $0$ |  | $3$ | $\frac{3}{2}$ | $\frac{3}{2}$ | $0$ | $\frac{3}{2}$ | $\frac{1}{4}(3-2r)$ | $\frac{3}{4}$ | $-\frac{1}{4}(3-2r)$ | $\frac{3}{4}$ |
| $\frac{\begin{matrix} A & b \end{matrix}}{\begin{matrix} A & b \end{matrix}}$ | $3$ | $\frac{3}{2}$ | $0$ | $-\frac{3}{2}$ | $0$ | $-\frac{r}{2}$ | $\frac{1}{4}(3-2r)$ | $-\frac{1}{4}(3-2r)$ | $-\frac{1}{4}(3-2r)$ |  | $3$ | $\frac{3}{2}$ | $\frac{3}{2}$ | $-\frac{3}{2}$ | $\frac{3}{2}$ | $-\frac{r}{2}$ | $\frac{1}{2}(3-r)$ | $-\frac{1}{2}(3-r)$ | $\frac{r}{2}$ |
| $\frac{\begin{matrix} A & B \end{matrix}}{\begin{matrix} a & B \end{matrix}}$ | $3$ | $0$ | $0$ | $\frac{3}{2}$ | $0$ | $\frac{1}{4}(3-2r)$ | $-\frac{1}{4}(3-2r)$ | $0$ | $0$ |  | $3$ | $0$ | $\frac{3}{2}$ | $\frac{3}{2}$ | $\frac{3}{2}$ | $\frac{1}{4}(3-2r)$ | $-\frac{1}{4}(3-2r)$ | $\frac{3}{4}$ | $\frac{3}{4}$ |
| $\frac{\begin{matrix} A & B \end{matrix}}{\begin{matrix} a & b \end{matrix}}$ | $3$ | $0$ | $0$ | $0$ | $0$ | $\frac{1}{4}(3-2r)$ | $0$ | $0$ | $0$ |  | $3$ | $0$ | $\frac{3}{2}$ | $0$ | $\frac{3}{2}$ | $\frac{1}{4}(3-2r)$ | $0$ | $0$ | $\frac{3}{4}$ |
| $\frac{\begin{matrix} A & b \end{matrix}}{\begin{matrix} a & b \end{matrix}}$ | $3$ | $0$ | $0$ | $-\frac{3}{2}$ | $0$ | $\frac{1}{4}(3-2r)$ | $\frac{1}{4}(3-2r)$ | $0$ | $0$ |  | $3$ | $0$ | $\frac{3}{2}$ | $-\frac{3}{2}$ | 3$\frac{3}{2}$ | $\frac{1}{4}(3-2r)$ | $\frac{1}{4}(3-2r)$ | $-\frac{3}{4}$ | $\frac{3}{4}$ |
| $\frac{\begin{matrix} a & B \end{matrix}}{\begin{matrix} a & B \end{matrix}}$ | $3$ | $-\frac{3}{2}$ | $0$ | $\frac{3}{2}$ | $0$ | $-\frac{r}{2}$ | $-\frac{1}{4}(3-2r)$ | $\frac{1}{4}(3-2r)$ | $-\frac{1}{4}(3-2r)$ |  | $3$ | $-\frac{3}{2}$ | $\frac{3}{2}$ | $\frac{3}{2}$ | $\frac{3}{2}$ | $-\frac{r}{2}$ | $-\frac{1}{2}(3-r)$ | $\frac{1}{2}(3-r)$ | $\frac{r}{2}$ |
| $\frac{\begin{matrix} a & B \end{matrix}}{\begin{matrix} a & b \end{matrix}}$ | $3$ | $-\frac{3}{2}$ | $0$ | $0$ | $0$ | $\frac{1}{4}(3-2r)$ | $0$ | $\frac{1}{4}(3-2r)$ | $0$ |  | $3$ | $-\frac{3}{2}$ | $\frac{3}{2}$ | $0$ | $\frac{3}{2}$ | $\frac{1}{4}(3-2r)$ | $-\frac{3}{4}$ | $\frac{1}{4}(3-2r)$ | $\frac{3}{4}$ |
| $\frac{\begin{matrix} a & b \end{matrix}}{\begin{matrix} a & b \end{matrix}}$ | $3$ | $-\frac{3}{2}$ | $0$ | $-\frac{3}{2}$ | $0$ | $\frac{1}{2}(3-r)$ | $\frac{1}{4}(3-2r)$ | $\frac{1}{4}(3-2r)$ | $\frac{1}{4}(3-2r)$ |  | $3$ | $-\frac{3}{2}$ | $\frac{3}{2}$ | $-\frac{3}{2}$ | $\frac{3}{2}$ | $\frac{1}{2}(3-r)$ | $-\frac{r}{2}$ | $-\frac{r}{2}$ | $\frac{1}{2}(3-r)$ |

**Table B10. Expected genetic values of *Z_6i_* under the F_∞_ and F_2_ metric model in F_2_-based aTTC design.**

| Genotype of  F_2_ plant | F_2_ metric model | | | | | | | | |  | F_∞_ metric model | | | | | | | | |
| --- | --- | --- | --- | --- | --- | --- | --- | --- | --- | --- | --- | --- | --- | --- | --- | --- | --- | --- | --- |
|  | $\mu$ | $a_{1}$ | $d_{1}$ | $a_{2}$ | $d_{2}$ | $i_{a_{1}a_{2}}$ | $i_{a_{1}d_{2}}$ | $i_{d_{1}a_{2}}$ | $i_{d_{1}d_{2}}$ |  | $\mu$ | $a_{1}$ | $d_{1}$ | $a_{2}$ | $d_{2}$ | $i_{a_{1}a_{2}}$ | $i_{a_{1}d_{2}}$ | $i_{d_{1}a_{2}}$ | $i_{d_{1}d_{2}}$ |
| $\frac{\begin{matrix} A & B \end{matrix}}{\begin{matrix} A & B \end{matrix}}$ | $1$ | $0$ | $\frac{1}{2}$ | $0$ | $\frac{1}{2}$ | $-r$ | $r$ | $r$ | $\frac{1}{4}(1-4r)$ |  | $1$ | $0$ | $1$ | $0$ | $1$ | $-r$ | $r$ | $r$ | $1-r$ |
| $\frac{\begin{matrix} A & B \end{matrix}}{\begin{matrix} A & b \end{matrix}}$ | $1$ | $0$ | $\frac{1}{2}$ | $0$ | $-\frac{1}{4}$ | $\frac{1}{2}(1-2r)$ | $-\frac{1}{4}$ | $-\frac{1}{2}(1-2r)$ | $\frac{1}{8}$ |  | $1$ | $0$ | $1$ | $0$ | $\frac{1}{2}$ | $\frac{1}{2}(1-2r)$ | $0$ | $-\frac{1}{2}(1-2r)$ | $\frac{1}{2}$ |
| $\frac{\begin{matrix} A & b \end{matrix}}{\begin{matrix} A & b \end{matrix}}$ | $1$ | $0$ | $\frac{1}{2}$ | $0$ | $\frac{1}{2}$ | $-(r+1)$ | $1-r$ | $r-1$ | $-\frac{1}{4}(3-4r)$ |  | $1$ | $0$ | $1$ | $0$ | $1$ | $-(1+r)$ | $1-r$ | $r-1$ | $r$ |
| $\frac{\begin{matrix} A & B \end{matrix}}{\begin{matrix} a & B \end{matrix}}$ | $1$ | $0$ | $-\frac{1}{4}$ | $0$ | $\frac{1}{2}$ | $-\frac{1}{2}(1+2r)$ | $-\frac{1}{2}(1-2r)$ | $-\frac{1}{4}$ | $\frac{1}{8}$ |  | $1$ | $0$ | $\frac{1}{2}$ | $0$ | $1$ | $\frac{1}{2}(1-2r)$ | $-\frac{1}{2}(1-2r)$ | $0$ | $\frac{1}{2}$ |
| $\frac{\begin{matrix} A & B \end{matrix}}{\begin{matrix} a & b \end{matrix}}$ | $1$ | $0$ | $-\frac{3}{4}$ | $0$ | $\frac{3}{4}$ | $\frac{1}{2}(1-2r)$ | $0$ | $0$ | $\frac{9}{16}$ |  | $1$ | $0$ | $\frac{1}{2}$ | $0$ | $\frac{1}{2}$ | $\frac{1}{2}(1-2r)$ | $0$ | $0$ | $\frac{1}{4}$ |
| $\frac{\begin{matrix} A & b \end{matrix}}{\begin{matrix} a & b \end{matrix}}$ | $1$ | $0$ | $-\frac{1}{4}$ | $0$ | $\frac{1}{2}$ | $\frac{1}{2}(1-2r)$ | $\frac{1}{2}(1-2r)$ | $\frac{1}{4}$ | $\frac{1}{8}$ |  | $1$ | $0$ | $\frac{1}{2}$ | $0$ | $1$ | $\frac{1}{2}(1-2r)$ | $\frac{1}{2}(1-2r)$ | $0$ | $\frac{1}{2}$ |
| $\frac{\begin{matrix} a & B \end{matrix}}{\begin{matrix} a & B \end{matrix}}$ | $1$ | $0$ | $\frac{1}{2}$ | $0$ | $\frac{1}{2}$ | $-(r+1)$ | $r-1$ | $1-r$ | $-\frac{1}{4}(3-4r)$ |  | $1$ | $0$ | $1$ | $0$ | $1$ | $-(1+r)$ | $r-1$ | $1-r$ | $r$ |
| $\frac{\begin{matrix} a & B \end{matrix}}{\begin{matrix} a & b \end{matrix}}$ | $1$ | $0$ | $\frac{1}{2}$ | $0$ | $-\frac{1}{4}$ | $\frac{1}{2}(1-2r)$ | $\frac{1}{4}$ | $\frac{1}{2}(1-2r)$ | $\frac{1}{8}$ |  | $1$ | $0$ | $1$ | $0$ | $\frac{1}{2}$ | $\frac{1}{2}(1-2r)$ | $0$ | $\frac{1}{2}(1-2r)$ | $\frac{1}{2}$ |
| $\frac{\begin{matrix} a & b \end{matrix}}{\begin{matrix} a & b \end{matrix}}$ | $1$ | $0$ | $\frac{1}{2}$ | $0$ | $\frac{1}{2}$ | $-r$ | $-r$ | $-r$ | $\frac{1}{4}(1-4r)$ |  | $1$ | $0$ | $1$ | $0$ | $1$ | $-r$ | $-r$ | $-r$ | $1-r$ |
