# Supplementary material for: Genetic dissection of main and epistatic effects of QTL based on augmented triple test cross design
Source: PLoS One. 2017 Dec 14;12(12):e0189054. doi: 10.1371/journal.pone.0189054 (PMC5730204; doi:10.1371/journal.pone.0189054)
Supplement: S3 Supporting Information — (DOC) [file pone.0189054.s003.doc]

**Statistical genetic models for mapping heterotic QTL in the RIL-based aTTC design under the F_2_ metric model**

According to the genetic expectations of Z_1i_ under the F_2_ metric model (Table A5), the phenotypic value of Z_1i_ can be described as

$Z_{1i}=2\mu+x_{a_{1}i}a_{1}+d_{1}+x_{a_{2}i}a_{2}+d_{2}+x_{a_{1}a_{2}i}i_{a_{1}a_{2}}+x_{a_{1}d_{2}i}i_{a_{1}d_{2}}+x_{d_{1}a_{2}i}i_{d_{1}a_{2}}+x_{d_{1}d_{2}i}i_{d_{1}d_{2}}+e_{1i}$ (A1)

where $\mu$ is the mean genotypic values of the four homozygotes in RIL population; $a_{k}$and $d_{k}$are additive and dominance effects of the $k\mathrm{th}$QTL (*k* = 1, 2); $i_{a_{1}a_{2}}$, $i_{a_{1}d_{2}}$, $i_{d_{1}a_{2}}$and $i_{d_{1}d_{2}}$are $additive\times additive$, $additive\times dominance$, $dominance\times additive$and $dominance\times dominance$interactions between two QTL, respectively; $x_{a_{1}i}$, $x_{a_{2}i}$, $x_{a_{1}a_{2}i}$, $x_{a_{1}d_{2}i}$, $x_{d_{1}a_{2}i}$ and $x_{d_{1}d_{2}i}$ are dummy variables and determined by the genotype of the $i\mathrm{th}$RIL line (Table A5); and $e_{1i}$is the residual error with an $N(0,\sigma_{1}^{2})$distribution. According to Table A5, $x_{a_{1}a_{2}i}=x_{d_{1}d_{2}i}+\frac{1}{2}$ , $x_{a_{1}d_{2}i}=-\frac{1}{2}x_{a_{2}i}$and $x_{d_{1}a_{2}i}=-\frac{1}{2}x_{a_{1}i}$, model (A1) can be reduced to

$Z_{1i}=\mu_{z_{1}}+x_{a_{1}i}a_{1}^{*}+x_{a_{2}i}a_{2}^{*}+x_{\overleftrightarrow{i}_{12}i}\overleftrightarrow{i}_{12}+e_{1i}$ (A2)

Where$\mu_{z_{1}}=2\mu-\frac{1}{2}i_{d_{1}d_{2}}$, $a_{1}^{*}=a_{1}-\frac{1}{2}i_{d_{1}a_{2}}$, $a_{2}^{*}=a_{2}-\frac{1}{2}i_{a_{1}d_{2}}$, $\overleftrightarrow{i}_{12}=i_{a_{1}a_{2}}+i_{d_{1}d_{2}}$ and $x_{\overleftrightarrow{i}_{12}i}=x_{a_{1}a_{2}i}=x_{d_{1}d_{2}i}+\frac{1}{2}$. If the quantitative trait was controlled by *q* QTL, model (A2) should be extended to

$Z_{1i}=\mu_{z_{1}}+\sum_{k=1}^{q} x_{a_{k}^{*}i}a_{k}^{*}+\sum_{k=1}^{q-1} \sum_{l=k+1}^{q} x_{\overleftrightarrow{i}_{_{kl}}i}\overleftrightarrow{i}_{kl}+e_{1i}$ (A3)

Where the model mean$\mu_{z_{1}}=2\mu-\frac{1}{2}\sum_{k\neq1}^{q} i_{d_{k}d_{l}}$; $a_{k}^{*}=a_{k}-\frac{1}{2}\sum_{l\neq k}^{q} i_{d_{k}a_{l}}$ is augmented additive effect of QTL *k*; $\overleftrightarrow{i}_{kl}=i_{a_{k}a_{l}}+i_{d_{k}d_{l}}$is augmented epistatic effect between QTL *k* and *l*. Coefficients $x_{a_{k}^{*}i}$and $x_{\overleftrightarrow{i}_{_{kl}}i}$are determined by genotypes of the $k\mathrm{th}$and $l\mathrm{th}$QTL for the $i\mathrm{th}$RIL line, as shown in Table 1.

Similarly, the phenotypic value of Z_2i_ can be described as

$Z_{2i}=a_{1}+u_{d_{1}i}d_{1}+a_{2}+u_{d_{2}i}d_{2}+u_{a_{1}a_{2}i}i_{a_{1}a_{2}}+u_{a_{1}d_{2}i}i_{a_{1}d_{2}}+u_{d_{1}a_{2}i}i_{d_{1}a_{2}}+e_{2i}$ (A4)

Where$u_{d_{1}i}$, $u_{d_{2}i}$, $u_{a_{1}a_{2}i}$, $u_{a_{1}d_{2}i}$ and $u_{d_{1}a_{2}i}$ are determined by the genotype of the $i\mathrm{th}$ RIL line (Table A6), $e_{2i}$ is the residual error with an $N(0,\sigma_{2}^{2})$distribution. According to Table A6,$u_{a_{1}d_{2}i}=u_{d_{1}a_{2}i}$and $u_{a_{1}a_{2}i}=-\frac{1}{2}\left(u_{d_{1}i}+u_{d_{2}i}\right)$, model (A4) can be reduced to

$Z_{2i}=\mu_{z_{2}}+u_{d_{1}i}d_{1}^{*}+u_{d_{2}i}d_{2}^{*}+u_{\tilde{i}_{12}i}\tilde{i}_{12}+e_{2i}$ (A5)

Where $\mu_{z_{2}}=a_{1}+a_{2}$, $d_{1}^{*}=d_{1}-\frac{1}{2}i_{a_{1}a_{2}}$, $d_{2}^{*}=d_{2}-\frac{1}{2}i_{a_{1}a_{2}}$, $\tilde{i}_{12}=i_{a_{1}d_{2}}+i_{d_{1}a_{2}}$ and $u_{\tilde{i}_{12}i}=u_{a_{1}d_{2}i}=u_{d_{1}a_{2}i}$. If the quantitative trait was controlled by *q* QTL, model (A5) should be extended to

$Z_{2i}=\mu_{z_{2}}+\sum_{k=1}^{q} u_{d_{k}^{*}i}d_{k}^{*}+\sum_{k=1}^{q-1} \sum_{l=k+1}^{q} u_{\tilde{i}_{_{kl}}i}\tilde{i}_{kl}+e_{2i}$ (A6)

Where the model mean $\mu_{z_{2}}=\sum_{k=1}^{q} a_{k}$; $d_{k}^{*}=d_{k}-\frac{1}{2}\sum_{l\neq k}^{q} i_{a_{k}a_{l}}$ is the augmented dominance effect of QTL $k$, $\tilde{i}_{kl}=i_{a_{k}d_{l}}+i_{d_{k}a_{l}}$is the augmented epistatic effect between QTL $k$ and $l$. Coefficients $u_{d_{k}^{*}i}$and $u_{\tilde{i}_{_{kl}}i}$are determined by genotypes of the $k\mathrm{th}$and $l\mathrm{th}$QTL for the $i\mathrm{th}$RIL line (Table 1).

Similarly, the phenotypic value of Z_3i_ can be described as

$$Z_{3i}=ri_{a_{1}a_{2}}+v_{a_{1}d_{2}i}i_{a_{1}d_{2}}+v_{d_{1}a_{2}i}i_{d_{1}a_{2}}+v_{d_{1}d_{2}i}i_{d_{1}d_{2}}+e_{3i}$$

$=\mu_{z3}+v_{a_{1}d_{2}i}i_{a_{1}d_{2}}+v_{d_{1}a_{2}i}i_{d_{1}a_{2}}+v_{d_{1}d_{2}i}i_{d_{1}d_{2}}+e_{3i}$ (A7)

Where $\mu_{z3}=ri_{a_{1}a_{2}}$, *r* is the recombination fraction between two QTL; dummy variables $v_{a_{1}d_{2}i}$,$v_{d_{1}a_{2}i}$, and $v_{d_{1}d_{2}i}$ are determined by the genotype of the $i\mathrm{th}$ RIL line (Table A7). $e_{3i}$ is the residual error with an $N(0,\sigma_{3}^{2})$distribution. Pure effects $i_{a_{1}d_{2}},i_{d_{1}a_{2}},i_{d_{1}d_{2}}$can be estimated directly.

In the same way, the phenotypic value of Z_4i_ can be described as

$Z_{4i}=\mu+\frac{1}{2}d_{1}+\frac{1}{2}d_{2}+w_{a_{1}a_{2}i}i_{a_{1}a_{2}}+w_{a_{1}d_{2}i}i_{a_{1}d_{2}}+w_{d_{1}a_{2}i}i_{d_{1}a_{2}}+w_{d_{1}d_{2}i}i_{d_{1}d_{2}}+e_{4i}$ (A8)

Where $w_{a_{1}a_{2}i}$, $w_{a_{1}d_{2}i}$, $w_{d_{1}a_{2}i}$ and $w_{d_{1}d_{2}i}$ are determined by the genotype of the $i\mathrm{th}$ RIL line (Table A8), $e_{4i}$ is the residual error with an $N(0,\sigma_{4}^{2})$distribution. According to Table A8, there are $w_{a_{1}d_{2}i}=-w_{d_{1}a_{2}i}$ and $w_{a_{1}a_{2}i}=\frac{1}{4}-w_{d_{1}d_{2}i}$. Therefore, model (A8) can be reduced to

$Z_{4i}=\mu_{z_{4}}+w_{\overrightarrow{i}_{_{12}}i}\overrightarrow{i}_{12}+w_{\overleftarrow{i}_{_{12}}i}\overleftarrow{i}_{12}+e_{4i}$ (A9)

Where $\mu_{z_{4}}=\mu+\frac{1}{2}\left(d_{1}+d_{2}\right)+\frac{1}{4}i_{d_{1}d_{2}}$, $\overrightarrow{i}_{12}=i_{a_{1}a_{2}}-i_{d_{1}d_{2}}$, $\overleftarrow{i}_{12}=i_{a_{1}d_{2}}-i_{d_{1}a_{2}}$, and$w_{\overrightarrow{i}_{_{12}}i}=w_{a_{1}a_{2}i}$,$w_{\overleftarrow{i}_{_{12}}i}=w_{a_{1}d_{2}i}$.

If the quantitative trait was controlled by *q* QTL, model (A9) can be extended to

$Z_{4i}=\mu_{z_{4}}+\sum_{k=1}^{q-1} \sum_{l=k+1}^{q} w_{\overrightarrow{i}_{_{kl}}i}\overrightarrow{i}_{_{kl}}+\sum_{k=1}^{q-1} \sum_{l=k+1}^{q} w_{\overleftarrow{i}_{_{\text{kl}}}i}\overleftarrow{i}_{_{kl}}+e_{4i}$ (A10)

Where the model mean$\mu_{z_{4}}=\mu+\frac{1}{2}\sum_{k=1}^{q} d_{k}+\frac{1}{4}\sum_{l\neq k}^{q} i_{d_{k}d_{l}}$, $\overrightarrow{i}_{kl}=i_{a_{k}a_{l}}-i_{d_{k}d_{l}}$, $\overleftarrow{i}_{kl}=i_{a_{k}d_{l}}-i_{d_{k}a_{l}}$ is the augmented epistatic effect between QTL $k$ and $l$. Coefficients $w_{\overrightarrow{i}_{_{kl}}i}$ and $w_{\overleftarrow{i}_{_{\text{kl}}}i}$ are determined by genotypes of the $k\mathrm{th}$ and $l\mathrm{th}$QTL for the $i\mathrm{th}$RIL line (Table 1).

In the same way, the phenotypic value of Z_5i_ can be described as

$Z_{5i}=3\mu+s_{a_{1}i}a_{1}+s_{a_{2}i}a_{2}+s_{a_{1}a_{2}i}i_{a_{1}a_{2}}+s_{a_{1}d_{2}i}i_{a_{1}d_{2}}+s_{d_{1}a_{2}i}i_{d_{1}a_{2}}+s_{d_{1}d_{2}i}i_{d_{1}d_{2}}+e_{5i}$ (A11)

Where $s_{a_{1}i}$, $s_{a_{2}i}$, $s_{a_{1}a_{2}i}$, $s_{a_{1}d_{2}i}$, $s_{d_{1}a_{2}i}$ and $s_{d_{1}d_{2}i}$ are determined by the genotype of the $i\mathrm{th}$ RIL line (Table A9), $e_{5i}$ is the residual error with an $N(0,\sigma_{5}^{2})$distribution. According to Table A9, model (A11) can be reduced to

$Z_{5i}=\mu_{z_{5}}+s_{a_{1}i}a_{1}+s_{a_{2}i}a_{2}+s_{a_{1}a_{2}i}i_{a_{1}a_{2}}+s_{a_{1}d_{2}i}i_{a_{1}d_{2}}+s_{d_{1}a_{2}i}i_{d_{1}a_{2}}+s_{d_{1}d_{2}i}i_{d_{1}d_{2}}+e_{5i}$ (A12)

Where the model mean $\mu_{z_{5}}=3\mu$. Pure effects $a_{1},a_{2},i_{a_{1}a_{2}},i_{a_{1}d_{2}},i_{d_{1}a_{2}},i_{d_{1}d_{2}}$ can be estimated directly.

In the same way, the phenotypic value of Z_6i_ can be described as

$Z_{6i}=\mu+t_{a_{1}a_{2}i}i_{a_{1}a_{2}}+t_{a_{1}d_{2}i}i_{a_{1}d_{2}}+t_{d_{1}a_{2}i}i_{d_{1}a_{2}}+t_{d_{1}d_{2}i}i_{d_{1}d_{2}}+e_{6i}$ (A13)

Where $t_{a_{1}a_{2}i}$, $t_{a_{1}d_{2}i}$, $t_{d_{1}a_{2}i}$ and $t_{d_{1}d_{2}i}$ are determined by the genotype of the $i\mathrm{th}$ RIL line (Table A10), $e_{6i}$ is the residual error with an $N(0,\sigma_{6}^{2})$distribution. Pure effects $i_{a_{1}a_{2}},i_{a_{1}d_{2}},i_{d_{1}a_{2}},i_{d_{1}d_{2}}$ can be calculated directly.

When transformation combination is Z_1_, Z_2_, and Z_5_, After performing QTL mapping, genetic parameter$a_{1},a_{2},i_{a_{1}a_{2}},i_{a_{1}d_{2}},i_{d_{1}a_{2}},i_{d_{1}d_{2}}$in $Z_{5i}$, $a_{1}^{*}$, $a_{2}^{*}$, $d_{1}^{*}$, and $d_{2}^{*}$ in $Z_{1i}$ and $Z_{2i}$ can be obtained. Because $a_{1}^{*}=a_{1}+\frac{1}{2}(\mathrm{i}_{\mathrm{a}_{1}\mathrm{d}_{2}}-\mathrm{i}_{\mathrm{d}_{1}\mathrm{a}_{2}})$, $a_{2}^{*}=a_{2}+\frac{1}{2}(\mathrm{i}_{d_{1}a_{2}}-\mathrm{i}_{a_{1}d_{2}})$, $d_{1}^{*}=d_{1}+\frac{1}{2}(\mathrm{i}_{a_{1}a_{2}}-\mathrm{i}_{d_{1}d_{2}})$, and $d_{2}^{*}=d_{2}+\frac{1}{2}(\mathrm{i}_{a_{1}a_{2}}-\mathrm{i}_{d_{1}d_{2}})$， with the estimation value of $d_{1}^{*}$, $d_{2}^{*}$, we can get $d_{1}$ and $d_{2}$ by $d_{1}=d_{1}^{*}-\frac{1}{2}(\mathrm{i}_{a_{1}a_{2}}-\mathrm{i}_{d_{1}d_{2}})$, $d_{2}=d_{2}^{*}-\frac{1}{2}(\mathrm{i}_{a_{1}a_{2}}-\mathrm{i}_{d_{1}d_{2}})$. Thus, all pure main and epistatic effects were obtained.

When transformation combination is Z_1_, Z_2_, and Z_6_, After performing QTL mapping, genetic parameter$i_{a_{1}a_{2}},i_{a_{1}d_{2}},i_{d_{1}a_{2}},i_{d_{1}d_{2}}$in $Z_{6i}$, $a_{1}^{*}$, $a_{2}^{*}$, $d_{1}^{*}$, and $d_{2}^{*}$ in $Z_{1i}$ and $Z_{2i}$ can be obtained. Because $a_{1}^{*}=a_{1}+\frac{1}{2}(\mathrm{i}_{\mathrm{a}_{1}\mathrm{d}_{2}}-\mathrm{i}_{\mathrm{d}_{1}\mathrm{a}_{2}})$, $a_{2}^{*}=a_{2}+\frac{1}{2}(\mathrm{i}_{d_{1}a_{2}}-\mathrm{i}_{a_{1}d_{2}})$, $d_{1}^{*}=d_{1}+\frac{1}{2}(\mathrm{i}_{a_{1}a_{2}}-\mathrm{i}_{d_{1}d_{2}})$, and $d_{2}^{*}=d_{2}+\frac{1}{2}(\mathrm{i}_{a_{1}a_{2}}-\mathrm{i}_{d_{1}d_{2}})$， with the estimation value of $a_{1}^{*}$, $a_{2}^{*}$,$d_{1}^{*}$ and $d_{2}^{*}$, we can get $a_{1}$, $a_{2}$,$d_{1}$ and $d_{2}$ by $a_{1}=a_{1}^{*}-\frac{1}{2}(\mathrm{i}_{a_{1}d_{2}}-\mathrm{i}_{d_{1}a_{2}})$, $a_{2}=a_{2}^{*}-\frac{1}{2}(\mathrm{i}_{d_{1}a_{2}}-\mathrm{i}_{a_{1}d_{2}})$, $d_{1}=d_{1}^{*}-\frac{1}{2}(\mathrm{i}_{a_{1}a_{2}}-\mathrm{i}_{d_{1}d_{2}})$ and $d_{2}=d_{2}^{*}-\frac{1}{2}(\mathrm{i}_{a_{1}a_{2}}-\mathrm{i}_{d_{1}d_{2}})$. Thus, all pure main and epistatic effects were obtained.
