# Supplementary material for: Genetic dissection of main and epistatic effects of QTL based on augmented triple test cross design
Source: PLoS One. 2017 Dec 14;12(12):e0189054. doi: 10.1371/journal.pone.0189054 (PMC5730204; doi:10.1371/journal.pone.0189054)
Supplement: S4 Supporting Information — (DOC) [file pone.0189054.s004.doc]

**Statistical genetic models for mapping heterotic QTL in the F_2_-based aTTC design under the F_∞_ metric model**

According to the expected genetic values of $Z_{1i}$ under the F_∞_ metric model (Table B5), the phenotypic value of $Z_{1i}$ can be described as:

$Z_{1i}=2\mu+x_{\text{a}_{1}i}a_{1}+d_{1}+x_{\text{a}_{2}i}a_{2}+d_{2}+x_{\text{a}_{1}\text{a}_{2}i}i_{a_{1}a_{2}}+x_{\text{a}_{1}\text{d}_{2}i}i_{a_{1}d_{2}}+x_{\text{d}_{1}\text{a}_{2}i}i_{d_{1}a_{2}}+x_{\text{d}_{1}\text{d}_{2}i}i_{d_{1}d_{2}}+e_{1i}$ (B1)

where $\mu$ is the mean genotypic values of four homozygotes; $a_{1}$ and $d_{1}$ are additive and dominance effects of QTL A; $a_{2}$ and $d_{2}$ are additive and dominance effects of QTL B; $i_{a_{1}a_{2}}$, $i_{a_{1}d_{2}}$, $i_{d_{1}a_{2}}$ and $i_{d_{1}d_{2}}$ are additive × additive, additive × dominance, dominance × additive and dominance × dominance epistatic effects; $x_{\text{a}_{1}i}$, $x_{\text{a}_{2}i}$, $x_{\text{a}_{1}\text{a}_{2}i}$, $x_{\text{a}_{1}\text{d}_{2}i}$, and are determined by the genotype of the *i*th F_2_ plant (Table B5); and is the residual error with an distribution. According to the results in Table B5, there are and . To solve the genetic parameters, model (B1) must be reduced to:

$Z_{1i}=\mu_{Z_{1}}+x_{\text{a}_{1}i}a_{1}^{*}+x_{\text{a}_{2}i}a_{2}^{*}+x_{\overleftrightarrow{\text{i}}_{12}i}\overleftrightarrow{i}_{12}+e_{1i}$ (B2)

where $\mu_{Z_{1}}=2\mu+d_{1}+d_{2}$, , , $\overleftrightarrow{i}_{12}=(i_{a_{1}a_{2}}+i_{d_{1}d_{2}})$ and $x_{\overleftrightarrow{\text{i}}_{12}i}=x_{\text{a}_{1}\text{a}_{2}i}=x_{\text{d}_{1}\text{d}_{2}i}$.

If the quantitative trait was controlled by $q$ QTL, model (B2) should be extended to:

$Z_{1i}=\mu_{Z_{1}}+\sum_{k=1}^{q} x_{a_{k}i}a_{k}^{*}+\sum_{k=1}^{q-1} \sum_{l=k+1}^{q} x_{\overleftrightarrow{i}_{kl}i}\overleftrightarrow{i}_{kl}+e_{1i}$ (B3)

where is model mean; the augmented additive effect of QTL $k$, which equals to the pure additive effect of QTL $k$ ($a_{k}$) add half the sum of the difference between *ad* and *da* epistatic effects of QTL $k$ with all other QTL in the whole genome; is augmented epistatic effect between QTL $k$ and $l$, which estimates the compounded effect of *aa* and *dd* epistatic effects between QTL $k$ and $l$. Coefficients and are determined by genotypes of the *k*th and *l*th QTL (marker) for the *i*th F_2_ plant. Model (B3) considers multiple QTL and all types of digenic epistasis and can be used to obtain $a_{k}^{*}$ and $\overleftrightarrow{i}_{kl}$, simultaneously.

In the same way, the phenotypic value of can be described as:

$Z_{2i}=a_{1}+u_{\text{d}_{1}i}d_{1}+a_{2}+u_{\text{d}_{2}i}d_{2}+u_{\text{a}_{1}\text{a}_{2}i}i_{a_{1}a_{2}}+u_{\text{a}_{1}\text{d}_{2}i}i_{a_{1}d_{2}}+u_{\text{d}_{1}\text{a}_{2}i}i_{d_{1}a_{2}}+u_{\text{d}_{1}\text{d}_{2}i}i_{d_{1}d_{2}}+e_{2i}$ (B4)

where $a_{1}$, $d_{1}$, $a_{2}$, $d_{2}$, $i_{a_{1}a_{2}}$, $i_{a_{1}d_{2}}$, $i_{d_{1}a_{2}}$ and $i_{d_{1}d_{2}}$ are same as those in model (A1); $u_{\text{d}_{1}i}$, $u_{\text{d}_{2}i}$, $u_{\text{a}_{1}\text{a}_{2}i}$, $u_{\text{a}_{1}\text{d}_{2}i}$, $u_{\text{d}_{1}\text{a}_{2}i}$ and $u_{\text{d}_{1}\text{d}_{2}i}$ are determined by the genotype of the *i*th F_2_ plant (Table B6), and $e_{2i}$ is the residual error with an $N(0,\sigma_{2}^{2})$ distribution. According to the results in Table B6, there are $u_{\text{a}_{1}\text{d}_{2}i}=u_{\text{d}_{1}\text{a}_{2}i}$ and . To solve the genetic parameters, model (B4) must be reduced to:

$Z_{2i}=\mu_{Z_{2}}+u_{\text{d}_{1}i}d_{1}^{*}+u_{\text{d}_{2}i}d_{2}^{*}+u_{\tilde{\text{i}}_{12}i}\tilde{i}_{12}+e_{2i}$ (B5)

where , , , $\tilde{i}_{12}=(i_{a_{1}d_{2}}+i_{d_{1}a_{2}})$ and $u_{\tilde{\text{i}}_{12}i}=u_{\text{a}_{1}\text{d}_{2}i}=u_{\text{d}_{1}\text{a}_{2}i}$.

If the quantitative trait was controlled by $q$ QTL, model (B5) should be extended to:

$Z_{2i}=\mu_{Z_{2}}+\sum_{k=1}^{q} u_{\text{d}_{\text{k}}i}d_{k}^{*}+\sum_{k=1}^{q-1} \sum_{l=k+1}^{q} u_{\tilde{\text{i}}_{\text{kl}}i}\tilde{i}_{kl}+e_{2i}$ (B6)

where is model mean; is the augmented dominance effect of QTL $k$, which equals to the pure dominance effect of QTL $k$ ($d_{k}$) minus half the sum of the difference between *aa* and *dd* epistatic effects of QTL $k$ with all other QTL in the whole genome; is augmented epistatic effect between QTL $k$ and $l$, which estimates the compounded effect of *ad* and *da* epistatic effects between QTL $k$ and $l$. Similarly to Model (B3), Model (B6) considers multiple QTL and all types of digenic epistasis and can be used to obtain $d_{k}^{*}$ and $\tilde{i}_{kl}$, simultaneously.

Similarly, the phenotypic value of can be described as:

(B7)

where ; is the recombination fraction between the 1st and 2nd QTL; , and are determined by the genotype of the *i*th F_2_ plant (Table B7); and is the residual error with an distribution. According to Model (B7), three types of pure epistatic effect (*ad*, *da* and *dd*) can be obtained in the analysis of the F_2_-based aTTC $Z_{3i}$ data with two-dimensional genome scans.

In the same way, the phenotypic value of $Z_{4i}$ can be described as

$Z_{4i}=\mu+w_{d_{1}i}d_{1}+w_{d_{2}i}d_{2}+w_{a_{1}a_{2}i}i_{a_{1}a_{2}}+w_{a_{1}d_{2}i}i_{a_{1}d_{2}}+w_{d_{1}a_{2}i}i_{d_{1}a_{2}}+w_{d_{1}d_{2}i}i_{d_{1}d_{2}}+e_{4i}$ (B8)

Where $w_{d_{1}i}$, $w_{d_{2}i}$, $w_{a_{1}a_{2}i}$, $w_{a_{1}d_{2}i}$, $w_{d_{1}a_{2}i}$ and $w_{d_{1}d_{2}i}$ are determined by the genotype of the $i\mathrm{th}$ F_2_ plant (Table B8), $e_{4i}$ is the residual error with an $N(0,\sigma_{4}^{2})$distribution. Pure effects $d_{1},d_{2},i_{a_{1}a_{2}},i_{a_{1}d_{2}},i_{d_{1}a_{2}},i_{d_{1}d_{2}}$ can be estimated directly.

In the same way, the phenotypic value of $Z_{5i}$ can be described as

$Z_{5i}=3\mu+s_{a_{1}i}a_{1}+\frac{3}{2}d_{1}+s_{a_{2}i}a_{2}+\frac{3}{2}d_{2}+s_{a_{1}a_{2}i}i_{a_{1}a_{2}}+s_{a_{1}d_{2}i}i_{a_{1}d_{2}}+s_{d_{1}a_{2}i}i_{d_{1}a_{2}}+s_{d_{1}d_{2}i}i_{d_{1}d_{2}}+e_{5i}$ (B9)

Where $s_{a_{1}i}$, $s_{a_{2}i}$, $s_{a_{1}a_{2}i}$, $s_{a_{1}d_{2}i}$, $s_{d_{1}a_{2}i}$ and $s_{d_{1}d_{2}i}$ are determined by the genotype of the $i\mathrm{th}$ F_2_ plant (Table B9), $e_{5i}$ is the residual error with an $N(0,\sigma_{5}^{2})$distribution. According to Table B9, model (B9) can be reduced to

$Z_{5i}=\mu_{z_{5}}+s_{a_{1}i}a_{1}+s_{a_{2}i}a_{2}+s_{a_{1}a_{2}i}i_{a_{1}a_{2}}+s_{a_{1}d_{2}i}i_{a_{1}d_{2}}+s_{d_{1}a_{2}i}i_{d_{1}a_{2}}+s_{d_{1}d_{2}i}i_{d_{1}d_{2}}+e_{5i}$ (B10)

Where the model mean $\mu_{z_{5}}=3\mu+\frac{3}{2}\left(d_{1}+d_{2}\right)$. Pure effects $a_{1},a_{2},i_{a_{1}a_{2}},i_{a_{1}d_{2}},i_{d_{1}a_{2}},i_{d_{1}d_{2}}$ can be estimated directly.

In the same way, the phenotypic value of $Z_{6i}$ can be described as

$Z_{6i}=\mu+t_{d_{1}i}d_{1}+t_{d_{2}i}d_{2}+t_{a_{1}a_{2}i}i_{a_{1}a_{2}}+t_{a_{1}d_{2}i}i_{a_{1}d_{2}}+t_{d_{1}a_{2}i}i_{d_{1}a_{2}}+t_{d_{1}d_{2}i}i_{d_{1}d_{2}}+e_{6i}$ (B11)

Where $t_{d_{1}i}$, $t_{d_{2}i}$, $t_{a_{1}a_{2}i}$, $t_{a_{1}d_{2}i}$, $t_{d_{1}a_{2}i}$ and $t_{d_{1}d_{2}i}$ are determined by the genotype of the $i\mathrm{th}$ F_2_ plant (Table B10), $e_{6i}$ is the residual error with an $N(0,\sigma_{6}^{2})$distribution. Pure effects $d_{1},d_{2},i_{a_{1}a_{2}},i_{a_{1}d_{2}},i_{d_{1}a_{2}},i_{d_{1}d_{2}}$ can be calculated directly.

**Statistical genetic models for mapping heterotic QTL in the F_2_-based aTTC design under the F_2_ metric model**

According to the expected genetic values of $Z_{1i}$ under the F_2_ metric model (Table B5), the phenotypic value of $Z_{1i}$ can be described as:

$Z_{1i}=2\mu+x_{\text{a}_{1}i}a_{1}+x_{\text{a}_{2}i}a_{2}+x_{\text{a}_{1}\text{a}_{2}i}i_{a_{1}a_{2}}+x_{\text{a}_{1}\text{d}_{2}i}i_{a_{1}d_{2}}+x_{\text{d}_{1}\text{a}_{2}i}i_{d_{1}a_{2}}+x_{\text{d}_{1}\text{d}_{2}i}i_{d_{1}d_{2}}+e_{1i}$ (B12)

where $\mu$ is the mean genotypic values of four homozygotes; $a_{1}$ and $d_{1}$ are additive and dominance effects of QTL A; $a_{2}$ and $d_{2}$ are additive and dominance effects of QTL B; $i_{a_{1}a_{2}}$, $i_{a_{1}d_{2}}$, $i_{d_{1}a_{2}}$ and $i_{d_{1}d_{2}}$ are additive × additive, additive × dominance, dominance × additive and dominance × dominance epistatic effects; $x_{\text{a}_{1}i}$, $x_{\text{a}_{2}i}$, $x_{\text{a}_{1}\text{a}_{2}i}$, $x_{\text{a}_{1}\text{d}_{2}i}$, and are determined by the genotype of the *i*th F_2_ plant (Table B5); and is the residual error with an distribution. According to the results in Table B5, there are , and . To solve the genetic parameters, model (B12) must be reduced to:

$Z_{1i}=\mu_{Z_{1}}+x_{\text{a}_{1}i}a_{1}^{*}+x_{\text{a}_{2}i}a_{2}^{*}+x_{\overleftrightarrow{\text{i}}_{12}i}\overleftrightarrow{i}_{12}+e_{1i}$ (B13)

where , , , $\overleftrightarrow{i}_{12}=i_{a_{1}a_{2}}+i_{d_{1}d_{2}}$ and .

If the quantitative trait was controlled by $q$ QTL, model (B13) should be extended to:

$Z_{1i}=\mu_{Z_{1}}+\sum_{k=1}^{q} x_{a_{k}i}a_{k}^{*}+\sum_{k=1}^{q-1} \sum_{l=k+1}^{q} x_{\overleftrightarrow{i}_{kl}i}\overleftrightarrow{i}_{kl}+e_{1i}$ (B14)

where is model mean; the augmented additive effect of QTL $k$, which equals to the pure additive effect of QTL $k$ ($a_{k}$) add half the sum of the difference between *ad* and *da* epistatic effects of QTL $k$ with all other QTL in the whole genome; is augmented epistatic effect between QTL $k$ and $l$, which estimates the compounded effect of *aa* and *dd* epistatic effects between QTL $k$ and $l$. Coefficients and are determined by genotypes of the *k*th and *l*th QTL (marker) for the *i*th F_2_ plant. Model (B14) considers multiple QTL and all types of digenic epistasis and can be used to obtain $a_{k}^{*}$ and $\overleftrightarrow{i}_{kl}$, simultaneously.

In the same way, the phenotypic value of can be described as:

$Z_{2i}=a_{1}+u_{\text{d}_{1}i}d_{1}+a_{2}+u_{\text{d}_{2}i}d_{2}+u_{\text{a}_{1}\text{a}_{2}i}i_{a_{1}a_{2}}+u_{\text{a}_{1}\text{d}_{2}i}i_{a_{1}d_{2}}+u_{\text{d}_{1}\text{a}_{2}i}i_{d_{1}a_{2}}+e_{2i}$ (B15)

where $a_{1}$, $d_{1}$, $a_{2}$, $d_{2}$, $i_{a_{1}a_{2}}$, $i_{a_{1}d_{2}}$, and $i_{d_{1}a_{2}}$ are same as those in model (A1); $u_{\text{d}_{1}i}$, $u_{\text{d}_{2}i}$, $u_{\text{a}_{1}\text{a}_{2}i}$, $u_{\text{a}_{1}\text{d}_{2}i}$, $u_{\text{d}_{1}\text{a}_{2}i}$ and $u_{\text{d}_{1}\text{d}_{2}i}$ are determined by the genotype of the *i*th F_2_ plant (Table B6), and $e_{2i}$ is the residual error with an $N(0,\sigma_{2}^{2})$ distribution. According to the results in Table B6, there are $u_{\text{a}_{1}\text{d}_{2}i}=u_{\text{d}_{1}\text{a}_{2}i}$ and . To solve the genetic parameters, model (B15) must be reduced to:

$Z_{2i}=\mu_{Z_{2}}+u_{\text{d}_{1}i}d_{1}^{*}+u_{\text{d}_{2}i}d_{2}^{*}+u_{\tilde{\text{i}}_{12}i}\tilde{i}_{12}+e_{2i}$ (B16)

where , , , $\tilde{i}_{12}=i_{a_{1}d_{2}}+i_{d_{1}a_{2}}$ and $u_{\tilde{\text{i}}_{12}i}=u_{\text{a}_{1}\text{d}_{2}i}=u_{\text{d}_{1}\text{a}_{2}i}$.

If the quantitative trait was controlled by $q$ QTL, model (B16) should be extended to:

$Z_{2i}=\mu_{Z_{2}}+\sum_{k=1}^{q} u_{\text{d}_{\text{k}}i}d_{k}^{*}+\sum_{k=1}^{q-1} \sum_{l=k+1}^{q} u_{\tilde{\text{i}}_{\text{kl}}i}\tilde{i}_{kl}+e_{2i}$ (B17)

where is model mean; is the augmented dominance effect of QTL $k$, which equals to the pure dominance effect of QTL $k$ ($d_{k}$) minus half the sum of the difference between *aa* and *dd* epistatic effects of QTL $k$ with all other QTL in the whole genome; is augmented epistatic effect between QTL $k$ and $l$, which estimates the compounded effect of *ad* and *da* epistatic effects between QTL $k$ and $l$. Similarly to Model (A3), Model (A6) considers multiple QTL and all types of digenic epistasis and can be used to obtain $d_{k}^{*}$ and $\tilde{i}_{kl}$, simultaneously.

Similarly, the phenotypic value of can be described as:

(B18)

where ; is the recombination fraction between the 1st and 2nd QTL; , and are determined by the genotype of the *i*th F_2_ plant (Table B7); and is the residual error with an distribution. According to Model (B18), three types of pure epistatic effect (*ad*, *da* and *dd*) can be obtained in the analysis of the F_2_-based aTTC $Z_{3i}$ data with two-dimensional genome scans.

In the same way, the phenotypic value of $Z_{4i}$ can be described as

$Z_{4i}=\mu+w_{d_{1}i}d_{1}+w_{d_{2}i}d_{2}+w_{a_{1}a_{2}i}i_{a_{1}a_{2}}+w_{a_{1}d_{2}i}i_{a_{1}d_{2}}+w_{d_{1}a_{2}i}i_{d_{1}a_{2}}+w_{d_{1}d_{2}i}i_{d_{1}d_{2}}+e_{4i}$ (B19)

Where $w_{d_{1}i}$, $w_{d_{2}i}$, $w_{a_{1}a_{2}i}$, $w_{a_{1}d_{2}i}$, $w_{d_{1}a_{2}i}$ and $w_{d_{1}d_{2}i}$ are determined by the genotype of the $i\mathrm{th}$ F_2_ plant (Table B8), $e_{4i}$ is the residual error with an $N(0,\sigma_{4}^{2})$distribution. Pure effects $d_{1},d_{2},i_{a_{1}a_{2}},i_{a_{1}d_{2}},i_{d_{1}a_{2}},i_{d_{1}d_{2}}$ can be estimated directly.

In the same way, the phenotypic value of $Z_{5i}$ can be described as

$Z_{5i}=3\mu+s_{a_{1}i}a_{1}+s_{a_{2}i}a_{2}+s_{a_{1}a_{2}i}i_{a_{1}a_{2}}+s_{a_{1}d_{2}i}i_{a_{1}d_{2}}+s_{d_{1}a_{2}i}i_{d_{1}a_{2}}+s_{d_{1}d_{2}i}i_{d_{1}d_{2}}+e_{5i}$ (B20)

Where $s_{a_{1}i}$, $s_{a_{2}i}$, $s_{a_{1}a_{2}i}$, $s_{a_{1}d_{2}i}$, $s_{d_{1}a_{2}i}$ and $s_{d_{1}d_{2}i}$ are determined by the genotype of the $i\mathrm{th}$ F_2_ plant (Table B9), $e_{5i}$ is the residual error with an $N(0,\sigma_{5}^{2})$distribution. According to Table B9, and model (11) can be reduced to

$Z_{5i}=\mu_{z_{5}}+s_{a_{1}a_{2}i}i_{a_{1}a_{2}}+s_{a_{1}d_{2}i}\overrightarrow{i}_{a_{1}d_{2}}+s_{d_{1}a_{2}i}\overleftarrow{i}_{d_{1}a_{2}}+s_{d_{1}d_{2}i}i_{d_{1}d_{2}}+e_{5i}$ (B21)

Where the model mean $\mu_{z_{5}}=3\mu$. and .

If the quantitative trait was controlled by $q$ QTL, model (B21) should be extended to:

$Z_{5i}=\mu_{Z_{5}}+\sum_{k=1}^{q-1} \sum_{l=k+1}^{q} s_{a_{k}a_{l}i}i_{a_{k}a_{l}}+\sum_{k=1}^{q-1} \sum_{l=k+1}^{q} s_{a_{k}d_{l}i}\overrightarrow{i}_{a_{k}d_{l}}+\sum_{k=1}^{q-1} \sum_{l=k+1}^{q} s_{d_{k}a_{l}i}\overleftarrow{i}_{d_{k}a_{l}}+\sum_{k=1}^{q-1} \sum_{l=k+1}^{q} s_{d_{k}d_{l}i}i_{d_{k}d_{l}}+e_{5i}$ (B22)

where $\mu_{z_{5}}=3\mu$ is model mean; and are the augmented epistatic effect between QTL $k$ and $l$, which estimates the compounded effect between QTL $k$ and $l$.

In the same way, the phenotypic value of $Z_{6i}$ can be described as

$Z_{6i}=\mu+t_{d_{1}i}d_{1}+t_{d_{2}i}d_{2}+t_{a_{1}a_{2}i}i_{a_{1}a_{2}}+t_{a_{1}d_{2}i}i_{a_{1}d_{2}}+t_{d_{1}a_{2}i}i_{d_{1}a_{2}}+t_{d_{1}d_{2}i}i_{d_{1}d_{2}}+e_{6i}$ (B23)

Where $t_{d_{1}i}$, $t_{d_{2}i}$, $t_{a_{1}a_{2}i}$, $t_{a_{1}d_{2}i}$, $t_{d_{1}a_{2}i}$ and $t_{d_{1}d_{2}i}$ are determined by the genotype of the $i\mathrm{th}$ F_2_ plant (Table B10), $e_{6i}$ is the residual error with an $N(0,\sigma_{6}^{2})$distribution. Pure effects $d_{1},d_{2},i_{a_{1}a_{2}},i_{a_{1}d_{2}},i_{d_{1}a_{2}},i_{d_{1}d_{2}}$ can be calculated directly.
