# Supplementary material for: Genetic dissection of main and epistatic effects of QTL based on augmented triple test cross design
Source: PLoS One. 2017 Dec 14;12(12):e0189054. doi: 10.1371/journal.pone.0189054 (PMC5730204; doi:10.1371/journal.pone.0189054)
Supplement: S5 Supporting Information — (ZIP) [file pone.0189054.s005.zip › read me.docx]

aTTC_design_F_inf_metric.m is a matlab script for generating genotype and phenotype data of Z1, Z2, Z3, Z4, Z5 and Z6.

get_Marker_simulation.m, RIL_QTLsimulation_F_inf.m, tcRIL_QTLsimulation_F_inf_BC_1.m, tcRIL_QTLsimulation_F_inf_BC_2.m, tcRIL_QTLsimulation_F_inf_BC_3.m are subfunctions script of aTTC_design_F_inf_metric( ).

get_Marker_simulation.m is a matlab script for generating genotype data of RIL. tcRIL_QTLsimulation_F_inf_BC_1.m, tcRIL_QTLsimulation_F_inf_BC_2.m and tcRIL_QTLsimulation_F_inf_BC_3.m are separate scripts for generating genotype and phenotype data of L1, L2 and L3.
