# Supplementary material for: Genetic dissection of main and epistatic effects of QTL based on augmented triple test cross design
Source: PLoS One. 2017 Dec 14;12(12):e0189054. doi: 10.1371/journal.pone.0189054 (PMC5730204; doi:10.1371/journal.pone.0189054)
Supplement: S11 Supporting Information — (DOC) [file pone.0189054.s011.doc]

**Table 1. QTL mapping results for Z_1_ in RIL-based aTTC design under the F_∞_ metric model in simulation study.**

| n | h | m | QTL_1_ | | | QTL_2_ | | | QTL_3_ | | | QTL_4_ | | | QTL_5_ | | | QTL_6_ | | | QTL_1_×QTL_2_ | | | | QTL_3_×QTL_4_ | | | | QTL_5_×QTL_6_ | | | |
| --- | --- | --- | --- | --- | --- | --- | --- | --- | --- | --- | --- | --- | --- | --- | --- | --- | --- | --- | --- | --- | --- | --- | --- | --- | --- | --- | --- | --- | --- | --- | --- | --- |
| parameter |  |  | *a_1_** | position | power | *a_2_** | position | power | *a_3_** | position | power | *a_4_** | position | power | *a_5_** | position | power | *a_6_** | position | power | *i_12_** | position | position | power | *i_34_** | position | position | power | *i_56_** | position | position | power |
|  | | | 3.0 | 20 |  | 1.6 | 36 |  | 1.1 | 45 |  | -3.1 | 70 |  | 1.5 | 80 |  | -1.5 | 95 |  | 2.2 | 20 | 36 |  | 2.2 | 45 | 70 |  | 2.2 | 80 | 95 |  |
| 800 | 0.8 | 10 | 3.002 | 20 | 1 | 1.595 | 36 | 1 | 1.097 | 45 | 1 | -3.099 | 70 | 1 | 1.496 | 80 | 1 | -1.500 | 95 | 1 | 2.193 | 20 | 36 | 1 | 2.200 | 45 | 70 | 1 | 2.196 | 80 | 95 | 1 |
|  |  |  | (.021) |  |  | (.023) |  |  | (.020) |  |  | (.025) |  |  | (.023) |  |  | (.021) |  |  | (.044) |  |  |  | (.040) |  |  |  | (.043) |  |  |  |
|  |  | 5 | 3.003 | 20 | 1 | 1.595 | 36 | 1 | 1.097 | 45 | 1 | -3.102 | 70 | 1 | 1.500 | 80 | 1 | -1.497 | 95 | 1 | 2.206 | 20 | 36 | 1 | 2.190 | 45 | 70 | 1 | 2.203 | 80 | 95 | 1 |
|  |  |  | (.027) |  |  | (.030) |  |  | (.031) |  |  | (.032) |  |  | (.034) |  |  | (.033) |  |  | (.057) |  |  |  | (.073) |  |  |  | (.067) |  |  |  |
|  | 0.5 | 10 | 2.992 | 20 | 1 | 1.604 | 36 | 1 | 1.097 | 45 | 1 | -3.097 | 70 | 1 | 1.495 | 80 | 1 | -1.500 | 95 | 1 | 2.198 | 20 | 36 | 1 | 2.189 | 45 | 70 | 1 | 2.191 | 80 | 95 | 1 |
|  |  |  | (.044) |  |  | (.049) |  |  | (.046) |  |  | (.048) |  |  | (.042) |  |  | (.047) |  |  | (.088) |  |  |  | (.077) |  |  |  | (.079) |  |  |  |
|  |  | 5 | 2.995 | 20 | 1 | 1.588 | 36 | 1 | 1.099 | 45 | 1 | -3.096 | 70 | 1 | 1.498 | 80 | 1 | -1.493 | 95 | 1 | 2.174 | 20 | 36 | 1 | 2.172 | 45 | 70 | 1 | 2.172 | 80 | 95 | 1 |
|  |  |  | (.061) |  |  | (.059) |  |  | (.065) |  |  | (.063) |  |  | (.056) |  |  | (.066) |  |  | (.139) |  |  |  | (.105) |  |  |  | (.120) |  |  |  |
|  | 0.2 | 10 | 2.996 | 20 | 1 | 1.582 | 36 | 1 | 1.094 | 45 | 1 | -3.100 | 70 | 1 | 1.479 | 80 | 1 | -1.466 | 95 | 1 | 2.159 | 20 | 36 | 1 | 2.140 | 45 | 70 | 1 | 2.155 | 80 | 95 | 1 |
|  |  |  | (.089) |  |  | (.089) |  |  | (.095) |  |  | (.101) |  |  | (.094) |  |  | (.097) |  |  | (.158) |  |  |  | (.181) |  |  |  | (.163) |  |  |  |
|  |  | 5 | 2.960 | 20 | 1 | 1.555 | 36 | 1 | 1.051 | 45 | 1 | -3.069 | 70 | 1 | 1.477 | 80 | 1 | -1.472 | 95 | 1 | 2.056 | 20 | 36 | 1 | 2.077 | 45 | 70 | 1 | 2.103 | 80 | 95 | 1 |
|  |  |  | (.113) |  |  | (.138) |  |  | (.116) |  |  | (.135) |  |  | (.134) |  |  | (.135) |  |  | (.258) |  |  |  | (.445) |  |  |  | (.273) |  |  |  |
| 400 | 0.8 | 10 | 3.001 | 20 | 1 | 1.602 | 36 | 1 | 1.098 | 45 | 1 | -3.099 | 70 | 1 | 1.495 | 80 | 1 | -1.499 | 95 | 1 | 2.195 | 20 | 36 | 1 | 2.192 | 45 | 70 | 1 | 2.195 | 80 | 95 | 1 |
|  |  |  | (.034) |  |  | (.030) |  |  | (.036) |  |  | (.028) |  |  | (.031) |  |  | (.029) |  |  | (.059) |  |  |  | (.066) |  |  |  | (.066) |  |  |  |
|  |  | 5 | 2.997 | 20 | 1 | 1.602 | 36 | 1 | 1.096 | 45 | 1 | -3.096 | 70 | 1 | 1.498 | 80 | 1 | -1.501 | 95 | 1 | 2.187 | 20 | 36 | 1 | 2.174 | 45 | 70 | 1 | 2.179 | 80 | 95 | 1 |
|  |  |  | (.046) |  |  | (.043) |  |  | (.050) |  |  | (.049) |  |  | (.052) |  |  | (.048) |  |  | (.081) |  |  |  | (.093) |  |  |  | (.087) |  |  |  |
|  | 0.5 | 10 | 2.997 | 20 | 1 | 1.596 | 36 | 1 | 1.081 | 45 | 1 | -3.094 | 70 | 1 | 1.492 | 80 | 1 | -1.485 | 95 | 1 | 2.187 | 20 | 36 | 1 | 2.175 | 45 | 70 | 1 | 2.183 | 80 | 95 | 1 |
|  |  |  | (.066) |  |  | (.065) |  |  | (.057) |  |  | (.073) |  |  | (.071) |  |  | (.059) |  |  | (.124) |  |  |  | (.128) |  |  |  | (.129) |  |  |  |
|  |  | 5 | 2.988 | 20 | 1 | 1.588 | 36 | 1 | 1.073 | 45 | 1 | -3.095 | 70 | 1 | 1.491 | 80 | 1 | -1.477 | 95 | 1 | 2.165 | 20 | 36 | 1 | 2.146 | 45 | 70 | 1 | 2.128 | 80 | 95 | 1 |
|  |  |  | (.091) |  |  | (.090) |  |  | (.094) |  |  | (.090) |  |  | (.088) |  |  | (.099) |  |  | (.175) |  |  |  | (.200) |  |  |  | (.189) |  |  |  |
|  | 0.2 | 10 | 2.984 | 20 | 1 | 1.557 | 36 | 1 | 1.051 | 45 | 1 | -3.081 | 70 | 1 | 1.444 | 80 | 1 | -1.496 | 95 | 1 | 2.152 | 20 | 36 | 0.99 | 2.078 | 45 | 70 | 1 | 2.126 | 80 | 95 | 1 |
|  |  |  | (.116) |  |  | (.135) |  |  | (.153) |  |  | (.114) |  |  | (.146) |  |  | (.148) |  |  | (.272) |  |  |  | (.246) |  |  |  | (.243) |  |  |  |
|  |  | 5 | 2.979 | 20 | 1 | 1.534 | 36 | 1 | 0.994 | 45 | 1 | -3.083 | 70 | 1 | 1.487 | 80 | 1 | -1.467 | 95 | 1 | 2.051 | 20 | 36 | 0.94 | 2.063 | 45 | 70 | 0.98 | 2.055 | 80 | 95 | 0.98 |
|  |  |  | (.187) |  |  | (.195) |  |  | (.192) |  |  | (.193) |  |  | (.200) |  |  | (.179) |  |  | (.530) |  |  |  | (.379) |  |  |  | (.573) |  |  |  |
| 200 | 0.8 | 10 | 2.998 | 20 | 1 | 1.592 | 36 | 1 | 1.094 | 45 | 1 | -3.100 | 70 | 0.995 | 1.493 | 80 | 1 | -1.497 | 95 | 1 | 2.196 | 20 | 36 | 1 | 2.199 | 45 | 70 | 1 | 2.183 | 80 | 95 | 1 |
|  |  |  | (.049) |  |  | (.046) |  |  | (.051) |  |  | (.044) |  |  | (.047) |  |  | (.043) |  |  | (.088) |  |  |  | (.088) |  |  |  | (.089) |  |  |  |
|  |  | 5 | 2.987 | 20 | 1 | 1.600 | 36 | 1 | 1.093 | 45 | 1 | -3.092 | 70 | 1 | 1.492 | 80 | 1 | -1.481 | 95 | 1 | 2.143 | 20 | 36 | 1 | 2.173 | 45 | 70 | 1 | 2.189 | 80 | 95 | 1 |
|  |  |  | (.060) |  |  | (.063) |  |  | (.066) |  |  | (.065) |  |  | (.066) |  |  | (.063) |  |  | (.122) |  |  |  | (.116) |  |  |  | (.122) |  |  |  |
|  | 0.5 | 10 | 2.993 | 20 | 1 | 1.590 | 36 | 1 | 1.091 | 45 | 1 | -3.097 | 70 | 1 | 1.494 | 80 | 1 | -1.471 | 95 | 1 | 2.184 | 20 | 36 | 1 | 2.156 | 45 | 70 | 1 | 2.159 | 80 | 95 | 1 |
|  |  |  | (.081) |  |  | (.098) |  |  | (.094) |  |  | (.085) |  |  | (.098) |  |  | (.090) |  |  | (.181) |  |  |  | (.173) |  |  |  | (.174) |  |  |  |
|  |  | 5 | 2.979 | 20 | 1 | 1.567 | 36 | 1 | 1.046 | 45 | 1 | -3.083 | 70 | 1 | 1.480 | 80 | 1 | -1.474 | 95 | 1 | 2.101 | 20 | 36 | 0.99 | 2.152 | 45 | 70 | 1 | 2.137 | 80 | 95 | 1 |
|  |  |  | (.127) |  |  | (.140) |  |  | (.142) |  |  | (.117) |  |  | (.125) |  |  | (.128) |  |  | (.302) |  |  |  | (.250) |  |  |  | (.258) |  |  |  |
|  | 0.2 | 10 | 2.950 | 20 | 1 | 1.540 | 36 | 1 | 1.025 | 45 | 0.97 | -3.039 | 70 | 0.995 | 1.463 | 80 | 0.995 | -1.484 | 95 | 0.99 | 2.033 | 20 | 36 | 0.97 | 2.070 | 45 | 70 | 0.93 | 2.121 | 80 | 95 | 0.95 |
|  |  |  | (.171) |  |  | (.173) |  |  | (.277) |  |  | (.185) |  |  | (.172) |  |  | (.199) |  |  | (.666) |  |  |  | (.287) |  |  |  | (.498) |  |  |  |
|  |  | 5 | 2.843 | 20 | 1 | 1.516 | 36 | 0.94 | 0.909 | 45 | 0.55 | -2.956 | 70 | 1 | 1.265 | 80 | 0.94 | -1.434 | 95 | 0.82 | 2.380 | 20 | 36 | 0.31 | 2.339 | 45 | 70 | 0.49 | 2.496 | 80 | 95 | 0.34 |
|  |  |  | (.295) |  |  | (.291) |  |  | (.628) |  |  | (.522) |  |  | (.475) |  |  | (.284) |  |  | (.379) |  |  |  | (.736) |  |  |  | (.403) |  |  |  |

Epistatic effects estimated in Z_1_ were multiplied by 2.

**Table 2. QTL mapping results for Z_2_ in RIL-based aTTC design under the F_∞_ metric model in simulation study.**

| n | h | m | | QTL_1_ | | | QTL_2_ | | | QTL_3_ | | | QTL_4_ | | | QTL_5_ | | | QTL_6_ | | | QTL_1_×QTL_2_ | | | | QTL_3_×QTL_4_ | | | | QTL_5_×QTL_6_ | | | |
| --- | --- | --- | --- | --- | --- | --- | --- | --- | --- | --- | --- | --- | --- | --- | --- | --- | --- | --- | --- | --- | --- | --- | --- | --- | --- | --- | --- | --- | --- | --- | --- | --- | --- |
| parameter |  |  | | *d_1_** | position | power | *d_2_** | position | power | *d_3_** | position | power | *d_4_** | position | power | *d_5_** | position | power | *d_6_** | position | power | *i_12_** | position | position | power | *i_34_** | position | position | power | *i_56_** | position | position | power |
|  | | | 1.6 | | 20 |  | -3.1 | 36 |  | 1.8 | 45 |  | 3.9 | 70 |  | -1.7 | 80 |  | -1.7 | 95 |  | 4.4 | 20 | 36 |  | -5.4 | 45 | 70 |  | 4.8 | 80 | 95 |  |
| 800 | 0.8 | 10 | | 1.601 | 20 | 1 | -3.103 | 36 | 1 | 1.796 | 45 | 1 | 3.895 | 70 | 1 | -1.700 | 80 | 1 | -1.699 | 95 | 1 | 4.398 | 20 | 36 | 1 | -5.404 | 45 | 70 | 1 | 4.801 | 80 | 95 | 1 |
|  |  |  | | (.020) |  |  | (.025) |  |  | (.023) |  |  | (.024) |  |  | (.022) |  |  | (.021) |  |  | (.047) |  |  |  | (.048) |  |  |  | (.045) |  |  |  |
|  |  | 5 | | 1.597 | 20 | 1 | -3.101 | 36 | 1 | 1.795 | 45 | 1 | 3.897 | 70 | 1 | -1.700 | 80 | 1 | -1.698 | 95 | 1 | 4.406 | 20 | 36 | 1 | -5.396 | 45 | 70 | 1 | 4.799 | 80 | 95 | 1 |
|  |  |  | | (.031) |  |  | (.031) |  |  | (.032) |  |  | (.035) |  |  | (.031) |  |  | (.034) |  |  | (.063) |  |  |  | (.059) |  |  |  | (.058) |  |  |  |
|  | 0.5 | 10 | | 1.596 | 20 | 1 | -3.099 | 36 | 1 | 1.785 | 45 | 1 | 3.896 | 70 | 1 | -1.690 | 80 | 1 | -1.701 | 95 | 1 | 4.370 | 20 | 36 | 1 | -5.390 | 45 | 70 | 1 | 4.794 | 80 | 95 | 1 |
|  |  |  | | (.050) |  |  | (.051) |  |  | (.045) |  |  | (.047) |  |  | (.045) |  |  | (.048) |  |  | (.083) |  |  |  | (.088) |  |  |  | (.086) |  |  |  |
|  |  | 5 | | 1.596 | 20 | 1 | -3.108 | 36 | 1 | 1.790 | 45 | 1 | 3.895 | 70 | 1 | -1.692 | 80 | 1 | -1.688 | 95 | 1 | 4.371 | 20 | 36 | 1 | -5.405 | 45 | 70 | 1 | 4.790 | 80 | 95 | 1 |
|  |  |  | | (.060) |  |  | (.064) |  |  | (.076) |  |  | (.056) |  |  | (.067) |  |  | (.061) |  |  | (.141) |  |  |  | (.135) |  |  |  | (.129) |  |  |  |
|  | 0.2 | 10 | | 1.585 | 20 | 1 | -3.100 | 36 | 1 | 1.787 | 45 | 1 | 3.881 | 70 | 1 | -1.699 | 80 | 1 | -1.689 | 95 | 1 | 4.366 | 20 | 36 | 1 | -5.380 | 45 | 70 | 1 | 4.773 | 80 | 95 | 1 |
|  |  |  | | (.090) |  |  | (.098) |  |  | (.096) |  |  | (.086) |  |  | (.082) |  |  | (.089) |  |  | (.198) |  |  |  | (.198) |  |  |  | (.166) |  |  |  |
|  |  | 5 | | 1.557 | 20 | 1 | -3.078 | 36 | 1 | 1.781 | 45 | 1 | 3.894 | 70 | 1 | -1.691 | 80 | 1 | -1.680 | 95 | 1 | 4.400 | 20 | 36 | 1 | -5.372 | 45 | 70 | 1 | 4.759 | 80 | 95 | 1 |
|  |  |  | | (.110) |  |  | (.121) |  |  | (.109) |  |  | (.132) |  |  | (.127) |  |  | (.117) |  |  | (.242) |  |  |  | (.231) |  |  |  | (.269) |  |  |  |
| 400 | 0.8 | 10 | | 1.593 | 20 | 1 | -3.106 | 36 | 1 | 1.789 | 45 | 1 | 3.899 | 70 | 1 | -1.693 | 80 | 1 | -1.698 | 95 | 1 | 4.390 | 20 | 36 | 1 | -5.396 | 45 | 70 | 1 | 4.805 | 80 | 95 | 1 |
|  |  |  | | (.031) |  |  | (.033) |  |  | (.032) |  |  | (.032) |  |  | (.033) |  |  | (.037) |  |  | (.063) |  |  |  | (.062) |  |  |  | (.069) |  |  |  |
|  |  | 5 | | 1.592 | 20 | 1 | -3.101 | 36 | 1 | 1.785 | 45 | 1 | 3.901 | 70 | 1 | -1.701 | 80 | 1 | -1.700 | 95 | 1 | 4.395 | 20 | 36 | 1 | -5.398 | 45 | 70 | 1 | 4.796 | 80 | 95 | 1 |
|  |  |  | | (.049) |  |  | (.047) |  |  | (.047) |  |  | (.045) |  |  | (.044) |  |  | (.044) |  |  | (.096) |  |  |  | (.091) |  |  |  | (.085) |  |  |  |
|  | 0.5 | 10 | | 1.576 | 20 | 1 | -3.105 | 36 | 1 | 1.798 | 45 | 1 | 3.890 | 70 | 1 | -1.683 | 80 | 1 | -1.702 | 95 | 1 | 4.376 | 20 | 36 | 1 | -5.407 | 45 | 70 | 1 | 4.758 | 80 | 95 | 1 |
|  |  |  | | (.057) |  |  | (.062) |  |  | (.065) |  |  | (.073) |  |  | (.063) |  |  | (.065) |  |  | (.122) |  |  |  | (.128) |  |  |  | (.136) |  |  |  |
|  |  | 5 | | 1.591 | 20 | 1 | -3.095 | 36 | 1 | 1.774 | 45 | 1 | 3.881 | 70 | 1 | -1.688 | 80 | 1 | -1.681 | 95 | 1 | 4.395 | 20 | 36 | 1 | -5.413 | 45 | 70 | 1 | 4.775 | 80 | 95 | 1 |
|  |  |  | | (.096) |  |  | (.091) |  |  | (.084) |  |  | (.086) |  |  | (.087) |  |  | (.079) |  |  | (.186) |  |  |  | (.192) |  |  |  | (.199) |  |  |  |
|  | 0.2 | 10 | | 1.559 | 20 | 1 | -3.118 | 36 | 1 | 1.771 | 45 | 1 | 3.903 | 70 | 1 | -1.676 | 80 | 1 | -1.648 | 95 | 1 | 4.327 | 20 | 36 | 1 | -5.325 | 45 | 70 | 1 | 4.761 | 80 | 95 | 1 |
|  |  |  | | (.135) |  |  | (.126) |  |  | (.125) |  |  | (.125) |  |  | (.136) |  |  | (.144) |  |  | (.266) |  |  |  | (.245) |  |  |  | (.231) |  |  |  |
|  |  | 5 | | 1.557 | 20 | 1 | -3.064 | 36 | 1 | 1.749 | 45 | 1 | 3.880 | 70 | 1 | -1.652 | 80 | 1 | -1.618 | 95 | 1 | 4.344 | 20 | 36 | 1 | -5.339 | 45 | 70 | 1 | 4.785 | 80 | 95 | 1 |
|  |  |  | | (.194) |  |  | (.183) |  |  | (.204) |  |  | (.180) |  |  | (.183) |  |  | (.354) |  |  | (.392) |  |  |  | (.357) |  |  |  | (.411) |  |  |  |
| 200 | 0.8 | 10 | | 1.594 | 20 | 1 | -3.090 | 36 | 1 | 1.795 | 45 | 1 | 3.901 | 70 | 1 | -1.689 | 80 | 1 | -1.692 | 95 | 1 | 4.397 | 20 | 36 | 1 | -5.402 | 45 | 70 | 1 | 4.798 | 80 | 95 | 1 |
|  |  |  | | (.047) |  |  | (.047) |  |  | (.045) |  |  | (.046) |  |  | (.047) |  |  | (.043) |  |  | (.096) |  |  |  | (.094) |  |  |  | (.085) |  |  |  |
|  |  | 5 | | 1.594 | 20 | 1 | -3.080 | 36 | 1 | 1.796 | 45 | 1 | 3.890 | 70 | 1 | -1.691 | 80 | 1 | -1.702 | 95 | 1 | 4.379 | 20 | 36 | 1 | -5.392 | 45 | 70 | 1 | 4.795 | 80 | 95 | 1 |
|  |  |  | | (.067) |  |  | (.057) |  |  | (.065) |  |  | (.061) |  |  | (.066) |  |  | (.068) |  |  | (.118) |  |  |  | (.129) |  |  |  | (.127) |  |  |  |
|  | 0.5 | 10 | | 1.594 | 20 | 1 | -3.100 | 36 | 1 | 1.788 | 45 | 1 | 3.899 | 70 | 1 | -1.671 | 80 | 1 | -1.691 | 95 | 1 | 4.393 | 20 | 36 | 1 | -5.377 | 45 | 70 | 1 | 4.749 | 80 | 95 | 1 |
|  |  |  | | (.100) |  |  | (.087) |  |  | (.092) |  |  | (.095) |  |  | (.097) |  |  | (.092) |  |  | (.201) |  |  |  | (.182) |  |  |  | (.188) |  |  |  |
|  |  | 5 | | 1.558 | 20 | 1 | -3.081 | 36 | 1 | 1.771 | 45 | 1 | 3.887 | 70 | 1 | -1.675 | 80 | 1 | -1.681 | 95 | 1 | 4.342 | 20 | 36 | 1 | -5.339 | 45 | 70 | 1 | 4.819 | 80 | 95 | 1 |
|  |  |  | | (.125) |  |  | (.123) |  |  | (.133) |  |  | (.122) |  |  | (.128) |  |  | (.138) |  |  | (.219) |  |  |  | (.275) |  |  |  | (.249) |  |  |  |
|  | 0.2 | 10 | | 1.569 | 20 | 1 | -3.076 | 36 | 1 | 1.782 | 45 | 1 | 3.891 | 70 | 1 | -1.659 | 80 | 1 | -1.642 | 95 | 1 | 4.394 | 20 | 36 | 1 | -5.349 | 45 | 70 | 1 | 4.763 | 80 | 95 | 1 |
|  |  |  | | (.183) |  |  | (.184) |  |  | (.167) |  |  | (.168) |  |  | (.179) |  |  | (.173) |  |  | (.375) |  |  |  | (.388) |  |  |  | (.376) |  |  |  |
|  |  | 5 | | 1.524 | 20 | 0.97 | -3.078 | 36 | 1 | 1.597 | 45 | 0.99 | 3.904 | 70 | 1 | -1.619 | 80 | 1 | -1.611 | 95 | 0.97 | 4.193 | 20 | 36 | 1 | -5.363 | 45 | 70 | 1 | 4.687 | 80 | 95 | 0.99 |
|  |  |  | | (.288) |  |  | (.258) |  |  | (.501) |  |  | (.293) |  |  | (.275) |  |  | (.459) |  |  | (.632) |  |  |  | (.608) |  |  |  | (1.01) |  |  |  |

Epistatic effects estimated in Z_2_ were multiplied by 2.

**Table 3. QTL mapping results for Z_5_ in RIL-based aTTC design under the F_∞_ metric model in simulation study.**

| n | h | m | QTL_1_ | | | QTL_2_ | | | QTL_3_ | | | QTL_4_ | | | QTL_5_ | | | QTL_6_ | | | QTL_1_×QTL_2_ | | | | QTL_3_×QTL_4_ | | | | QTL_5_×QTL_6_ | | | |
| --- | --- | --- | --- | --- | --- | --- | --- | --- | --- | --- | --- | --- | --- | --- | --- | --- | --- | --- | --- | --- | --- | --- | --- | --- | --- | --- | --- | --- | --- | --- | --- | --- |
| parameter |  |  | *a_1_** | position | power | *a_2_** | position | power | *a_3_** | position | power | *a_4_** | position | power | *a_5_** | position | power | *a_6_** | position | power | *i_12_** | position | position | power | *i_34_** | position | position | power | *i_56_** | position | position | power |
|  | | |  | 20 |  |  | 36 |  |  | 45 |  |  | 70 |  |  | 80 |  |  | 95 |  |  | 20 | 36 |  |  | 45 | 70 |  |  | 80 | 95 |  |
| 800 | 0.8 | 10 | 4.151 | 20 | 1 | 3.240 | 36 | 1 | 0.144 | 45 | 0.09 | -5.057 | 70 | 1 | 1.706 | 80 | 1 | -1.254 | 95 | 1 | 2.748 | 20 | 36 | 1 | -0.229 | 45 | 70 | 0.09 | 2.938 | 80 | 95 | 1 |
|  |  |  | (.024) |  |  | (.025) |  |  | (.032) |  |  | (.025) |  |  | (.027) |  |  | (.023) |  |  | (.024) |  |  |  | (.008) |  |  |  | (.026) |  |  |  |
|  |  | 5 | 4.145 | 20 | 1 | 3.232 | 36 | 1 | 0.174 | 45 | 0.03 | -5.047 | 70 | 1 | 1.722 | 80 | 1 | -1.265 | 95 | 1 | 2.754 | 20 | 36 | 1 | -0.255 | 45 | 70 | 0.05 | 2.917 | 80 | 95 | 1 |
|  |  |  | (.035) |  |  | (.032) |  |  | (.019) |  |  | (.033) |  |  | (.037) |  |  | (.033) |  |  | (.037) |  |  |  | (.013) |  |  |  | (.035) |  |  |  |
|  | 0.5 | 10 | 4.161 | 20 | 1 | 3.249 | 36 | 1 | 0.218 | 45 | 0.01 | -5.051 | 70 | 1 | 1.728 | 80 | 1 | -1.268 | 95 | 1 | 2.736 | 20 | 36 | 1 | -0.287 | 45 | 70 | 0.01 | 2.916 | 80 | 95 | 1 |
|  |  |  | (.046) |  |  | (.049) |  |  | NA |  |  | (.053) |  |  | (.050) |  |  | (.050) |  |  | (.054) |  |  |  | NA |  |  |  | (.049) |  |  |  |
|  |  | 5 | 4.132 | 20 | 1 | 3.248 | 36 | 1 | NA | 45 | 0 | -5.035 | 70 | 1 | 1.713 | 80 | 1 | -1.262 | 95 | 1 | 2.741 | 20 | 36 | 1 | NA | 45 | 70 | 0 | 2.917 | 80 | 95 | 1 |
|  |  |  | (.062) |  |  | (.075) |  |  | NA |  |  | (.067) |  |  | (.078) |  |  | (.075) |  |  | (.070) |  |  |  | NA |  |  |  | (.067) |  |  |  |
|  | 0.2 | 10 | 4.147 | 20 | 1 | 3.230 | 36 | 1 | NA | 45 | 0 | -5.062 | 70 | 1 | 1.702 | 80 | 1 | -1.256 | 95 | 1 | 2.731 | 20 | 36 | 1 | NA | 45 | 70 | 0 | 2.901 | 80 | 95 | 1 |
|  |  |  | (.094) |  |  | (.115) |  |  | NA |  |  | (.107) |  |  | (.093) |  |  | (.100) |  |  | (.098) |  |  |  | NA |  |  |  | (.094) |  |  |  |
|  |  | 5 | 4.124 | 20 | 1 | 3.190 | 36 | 1 | NA | 45 | 0 | -5.005 | 70 | 1 | 1.648 | 80 | 1 | -1.198 | 95 | 1 | 2.706 | 20 | 36 | 1 | NA | 45 | 70 | 0 | 2.904 | 80 | 95 | 1 |
|  |  |  | (.159) |  |  | (.119) |  |  | NA |  |  | (.161) |  |  | (.143) |  |  | (.134) |  |  | (.149) |  |  |  | NA |  |  |  | (.126) |  |  |  |
| 400 | 0.8 | 10 | 4.155 | 20 | 1 | 3.240 | 36 | 1 | 0.142 | 45 | 0.62 | -5.053 | 70 | 1 | 1.727 | 80 | 1 | -1.273 | 95 | 1 | 2.746 | 20 | 36 | 1 | -0.180 | 45 | 70 | 0.91 | 2.919 | 80 | 95 | 1 |
|  |  |  | (.036) |  |  | (.036) |  |  | (.020) |  |  | (.038) |  |  | (.034) |  |  | (.036) |  |  | (.035) |  |  |  | (.031) |  |  |  | (.036) |  |  |  |
|  |  | 5 | 4.147 | 20 | 1 | 3.252 | 36 | 1 | 0.199 | 45 | 0.13 | -5.038 | 70 | 1 | 1.712 | 80 | 1 | -1.271 | 95 | 1 | 2.743 | 20 | 36 | 1 | -0.215 | 45 | 70 | 0.46 | 2.918 | 80 | 95 | 1 |
|  |  |  | (.045) |  |  | (.055) |  |  | (.034) |  |  | (.048) |  |  | (.050) |  |  | (.049) |  |  | (.055) |  |  |  | (.040) |  |  |  | (.048) |  |  |  |
|  | 0.5 | 10 | 4.121 | 20 | 1 | 3.218 | 36 | 1 | 0.290 | 45 | 0.01 | -5.081 | 70 | 1 | 1.727 | 80 | 1 | -1.260 | 95 | 1 | 2.734 | 20 | 36 | 1 | -0.296 | 45 | 70 | 0.07 | 2.917 | 80 | 95 | 1 |
|  |  |  | (.067) |  |  | (.067) |  |  | NA |  |  | (.070) |  |  | (.065) |  |  | (.068) |  |  | (.080) |  |  |  | (.028) |  |  |  | (.073) |  |  |  |
|  |  | 5 | 4.133 | 20 | 1 | 3.262 | 36 | 1 | NA | 45 | 0 | -5.024 | 70 | 1 | 1.695 | 80 | 1 | -1.271 | 95 | 1 | 2.750 | 20 | 36 | 1 | NA | 45 | 70 | 0 | 2.925 | 80 | 95 | 1 |
|  |  |  | (.094) |  |  | (.113) |  |  | NA |  |  | (.100) |  |  | (.107) |  |  | (.096) |  |  | (.107) |  |  |  | NA |  |  |  | (.105) |  |  |  |
|  | 0.2 | 10 | 4.118 | 20 | 1 | 3.216 | 36 | 1 | NA | 45 | 0 | -5.026 | 70 | 1 | 1.696 | 80 | 1 | -1.216 | 95 | 0.99 | 2.721 | 20 | 36 | 1 | NA | 45 | 70 | 0 | 2.861 | 80 | 95 | 1 |
|  |  |  | (.130) |  |  | (.149) |  |  | NA |  |  | (.150) |  |  | (.129) |  |  | (.158) |  |  | (.140) |  |  |  | NA |  |  |  | (.131) |  |  |  |
|  |  | 5 | 4.155 | 20 | 1 | 3.191 | 36 | 1 | NA | 45 | 0 | -5.025 | 70 | 1 | 1.607 | 80 | 1 | -1.216 | 95 | 0.9 | 2.680 | 20 | 36 | 1 | NA | 45 | 70 | 0 | 2.893 | 80 | 95 | 1 |
|  |  |  | (.229) |  |  | (.202) |  |  | NA |  |  | (.203) |  |  | (.237) |  |  | (.160) |  |  | (.212) |  |  |  | NA |  |  |  | (.218) |  |  |  |
| 200 | 0.8 | 10 | 4.158 | 20 | 1 | 3.225 | 36 | 1 | 0.208 | 45 | 0.09 | -5.064 | 70 | 1 | 1.711 | 80 | 1 | -1.263 | 95 | 1 | 2.749 | 20 | 36 | 1 | -0.225 | 45 | 70 | 0.4 | 2.914 | 80 | 95 | 1 |
|  |  |  | (.059) |  |  | (.060) |  |  | 0.030 |  |  | (.044) |  |  | (.054) |  |  | (.048) |  |  | (.052) |  |  |  | (.043) |  |  |  | (.050) |  |  |  |
|  |  | 5 | 4.139 | 20 | 1 | 3.239 | 36 | 1 | NA | 45 | 0 | -5.053 | 70 | 1 | 1.719 | 80 | 1 | -1.261 | 95 | 1 | 2.761 | 20 | 36 | 1 | -0.260 | 45 | 70 | 0.01 | 2.903 | 80 | 95 | 1 |
|  |  |  | (.076) |  |  | (.065) |  |  | NA |  |  | (.070) |  |  | (.070) |  |  | (.069) |  |  | (.067) |  |  |  | NA |  |  |  | (.076) |  |  |  |
|  | 0.5 | 10 | 4.148 | 20 | 1 | 3.269 | 36 | 1 | NA | 45 | 0 | -5.038 | 70 | 1 | 1.730 | 80 | 1 | -1.253 | 95 | 1 | 2.746 | 20 | 36 | 1 | -0.367 | 45 | 70 | 0.01 | 2.929 | 80 | 95 | 1 |
|  |  |  | (.104) |  |  | (.099) |  |  | NA |  |  | (.093) |  |  | (.109) |  |  | (.109) |  |  | (.091) |  |  |  | NA |  |  |  | (.116) |  |  |  |
|  |  | 5 | 4.128 | 20 | 1 | 3.246 | 36 | 1 | NA | 45 | 0 | -5.021 | 70 | 1 | 1.675 | 80 | 1 | -1.234 | 95 | 1 | 2.723 | 20 | 36 | 1 | NA | 45 | 70 | 0 | 2.912 | 80 | 95 | 1 |
|  |  |  | (.158) |  |  | (.134) |  |  | NA |  |  | (.133) |  |  | (.140) |  |  | (.137) |  |  | (.145) |  |  |  | NA |  |  |  | (.140) |  |  |  |
|  | 0.2 | 10 | 4.103 | 20 | 1 | 3.161 | 36 | 1 | NA | 45 | 0 | -5.024 | 70 | 1 | 1.606 | 80 | 1 | -1.217 | 95 | 0.85 | 2.756 | 20 | 36 | 1 | NA | 45 | 70 | 0 | 2.855 | 80 | 95 | 1 |
|  |  |  | (.186) |  |  | (.190) |  |  | NA |  |  | (.201) |  |  | (.207) |  |  | (.198) |  |  | (.230) |  |  |  | NA |  |  |  | (.198) |  |  |  |
|  |  | 5 | 3.996 | 20 | 1 | 3.330 | 36 | 1 | NA | 45 | 0 | -5.049 | 70 | 1 | 1.688 | 80 | 0.74 | -1.491 | 95 | 0.21 | 2.595 | 20 | 36 | 1 | NA | 45 | 70 | 0 | 2.709 | 80 | 95 | 1 |
|  |  |  | (.301) |  |  | (.293) |  |  | NA |  |  | (.281) |  |  | (.235) |  |  | (.139) |  |  | (.305) |  |  |  | NA |  |  |  | (.363) |  |  |  |

Epistatic effects estimated in Z_5_ were multiplied by 2.

**Table 4. QTL mapping results for Z_6_ in RIL-based aTTC design under the F_∞_ metric model in simulation study.**

| n | h | m | QTL_1_ | | | QTL_2_ | | | QTL_3_ | | | QTL_4_ | | | QTL_5_ | | | QTL_6_ | | | QTL_1_×QTL_2_ | | | | QTL_3_×QTL_4_ | | | | QTL_5_×QTL_6_ | | | |
| --- | --- | --- | --- | --- | --- | --- | --- | --- | --- | --- | --- | --- | --- | --- | --- | --- | --- | --- | --- | --- | --- | --- | --- | --- | --- | --- | --- | --- | --- | --- | --- | --- |
| parameter |  |  | *a_1_** | position | power | *a_2_** | position | power | *a_3_** | position | power | *a_4_** | position | power | *a_5_** | position | power | *a_6_** | position | power | *i_12_** | position | position | power | *i_34_** | position | position | power | *i_56_** | position | position | power |
|  | | |  | 20 |  |  | 36 |  |  | 45 |  |  | 70 |  |  | 80 |  |  | 95 |  |  | 20 | 36 |  |  | 45 | 70 |  |  | 80 | 95 |  |
| 800 | 0.8 | 10 | 0.498 | 20 | 1 | 0.494 | 36 | 1 | -1.897 | 45 | 1 | -1.897 | 70 | 1 | 0.439 | 80 | 1 | 0.446 | 95 | 1 | 0.599 | 20 | 36 | 1 | -1.894 | 45 | 70 | 1 | 0.851 | 80 | 95 | 1 |
|  |  |  | (.030) |  |  | (.032) |  |  | (.027) |  |  | (.029) |  |  | (.031) |  |  | (.034) |  |  | (.026) |  |  |  | (.031) |  |  |  | (.033) |  |  |  |
|  |  | 5 | 0.490 | 20 | 1 | 0.489 | 36 | 1 | -1.904 | 45 | 1 | -1.898 | 70 | 1 | 0.431 | 80 | 1 | 0.440 | 95 | 1 | 0.591 | 20 | 36 | 1 | -1.894 | 45 | 70 | 1 | 0.845 | 80 | 95 | 1 |
|  |  |  | (.047) |  |  | (.048) |  |  | (.042) |  |  | (.042) |  |  | (.042) |  |  | (.043) |  |  | (.040) |  |  |  | (.045) |  |  |  | (.041) |  |  |  |
|  | 0.5 | 10 | 0.485 | 20 | 1 | 0.479 | 36 | 1 | -1.881 | 45 | 1 | -1.895 | 70 | 1 | 0.422 | 80 | 1 | 0.427 | 95 | 1 | 0.588 | 20 | 36 | 1 | -1.893 | 45 | 70 | 1 | 0.829 | 80 | 95 | 1 |
|  |  |  | (.066) |  |  | (.063) |  |  | (.061) |  |  | (.064) |  |  | (.061) |  |  | (.067) |  |  | (.058) |  |  |  | (.055) |  |  |  | (.064) |  |  |  |
|  |  | 5 | 0.487 | 20 | 0.82 | 0.474 | 36 | 0.79 | -1.891 | 45 | 1 | -1.898 | 70 | 1 | 0.443 | 80 | 0.63 | 0.457 | 95 | 0.57 | 0.547 | 20 | 36 | 0.98 | -1.891 | 45 | 70 | 1 | 0.820 | 80 | 95 | 1 |
|  |  |  | (.079) |  |  | (.083) |  |  | (.098) |  |  | (.078) |  |  | (.068) |  |  | (.072) |  |  | (.097) |  |  |  | (.081) |  |  |  | (.092) |  |  |  |
|  | 0.2 | 10 | 0.628 | 20 | 0.21 | 0.609 | 36 | 0.17 | -1.910 | 45 | 1 | -1.896 | 70 | 1 | 0.641 | 80 | 0.06 | 0.594 | 95 | 0.11 | 0.660 | 20 | 36 | 0.46 | -1.887 | 45 | 70 | 1 | 0.797 | 80 | 95 | 0.91 |
|  |  |  | (.058) |  |  | (.054) |  |  | (.123) |  |  | (.139) |  |  | (.062) |  |  | (.067) |  |  | (.083) |  |  |  | (.134) |  |  |  | (.117) |  |  |  |
|  |  | 5 | NA | 20 | 0 | 0.814 | 36 | 0.01 | -1.843 | 45 | 1 | -1.722 | 70 | 1 | NA | 80 | 0 | NA | 95 | 0 | 0.844 | 20 | 36 | 0.03 | -1.801 | 45 | 70 | 1 | 0.913 | 80 | 95 | 0.28 |
|  |  |  | NA |  |  | NA |  |  | (.177) |  |  | (.162) |  |  | NA |  |  | NA |  |  | (.017) |  |  |  | (.164) |  |  |  | (.090) |  |  |  |
| 400 | 0.8 | 10 | 0.487 | 20 | 1 | 0.484 | 36 | 1 | -1.899 | 45 | 1 | -1.904 | 70 | 1 | 0.436 | 80 | 1 | 0.438 | 95 | 1 | 0.587 | 20 | 36 | 1 | -1.890 | 45 | 70 | 1 | 0.838 | 80 | 95 | 1 |
|  |  |  | (.041) |  |  | (.045) |  |  | (.047) |  |  | (.048) |  |  | (.040) |  |  | (.048) |  |  | (.041) |  |  |  | (.047) |  |  |  | (.043) |  |  |  |
|  |  | 5 | 0.479 | 20 | 1 | 0.475 | 36 | 1 | -1.893 | 45 | 1 | -1.906 | 70 | 1 | 0.422 | 80 | 1 | 0.418 | 95 | 1 | 0.588 | 20 | 36 | 1 | -1.893 | 45 | 70 | 1 | 0.841 | 80 | 95 | 1 |
|  |  |  | (.067) |  |  | (.066) |  |  | (.064) |  |  | (.073) |  |  | (.065) |  |  | (.068) |  |  | (.054) |  |  |  | (.068) |  |  |  | (.068) |  |  |  |
|  | 0.5 | 10 | 0.477 | 20 | 0.79 | 0.480 | 36 | 0.77 | -1.870 | 45 | 1 | -1.908 | 70 | 1 | 0.451 | 80 | 0.69 | 0.465 | 95 | 0.62 | 0.588 | 20 | 36 | 0.97 | -1.877 | 45 | 70 | 1 | 0.828 | 80 | 95 | 1 |
|  |  |  | (.075) |  |  | (.074) |  |  | (.093) |  |  | (.087) |  |  | (.069) |  |  | (.067) |  |  | (.093) |  |  |  | (.081) |  |  |  | (.107) |  |  |  |
|  |  | 5 | 0.606 | 20 | 0.26 | 0.575 | 36 | 0.16 | -1.823 | 45 | 1 | -1.820 | 70 | 1 | 0.617 | 80 | 0.24 | 0.584 | 95 | 0.08 | 0.647 | 20 | 36 | 0.33 | -1.833 | 45 | 70 | 1 | 0.871 | 80 | 95 | 0.97 |
|  |  |  | (.087) |  |  | (.040) |  |  | (.121) |  |  | (.133) |  |  | (.070) |  |  | (.048) |  |  | (.080) |  |  |  | (.115) |  |  |  | (.135) |  |  |  |
|  | 0.2 | 10 | NA | 20 | 0 | 0.900 | 36 | 0.01 | -2.021 | 45 | 1 | -1.856 | 70 | 1 | NA | 80 | 0 | NA | 95 | 0 | 0.866 | 20 | 36 | 0.01 | -1.841 | 45 | 70 | 1 | 0.990 | 80 | 95 | 0.24 |
|  |  |  | NA |  |  | NA |  |  | (.196) |  |  | (.163) |  |  | NA |  |  | NA |  |  | NA |  |  |  | (.228) |  |  |  | (.131) |  |  |  |
|  |  | 5 | 1.128 | 20 | 0.01 | NA | 36 | 0 | -1.776 | 45 | 1 | -1.766 | 70 | 0.96 | NA | 80 | 0 | NA | 95 | 0 | NA | 20 | 36 | 0 | -1.782 | 45 | 70 | 0.99 | 1.273 | 80 | 95 | 0.04 |
|  |  |  | NA |  |  | NA |  |  | (.234) |  |  | (.219) |  |  | NA |  |  | NA |  |  | NA |  |  |  | (.229) |  |  |  | (.064) |  |  |  |
| 200 | 0.8 | 10 | 0.475 | 20 | 0.99 | 0.454 | 36 | 0.97 | -1.897 | 45 | 1 | -1.892 | 70 | 1 | 0.433 | 80 | 0.97 | 0.426 | 95 | 0.95 | 0.585 | 20 | 36 | 0.99 | -1.867 | 45 | 70 | 1 | 0.839 | 80 | 95 | 1 |
|  |  |  | (.072) |  |  | (.078) |  |  | (.063) |  |  | (.067) |  |  | (.079) |  |  | (.074) |  |  | (.068) |  |  |  | (.078) |  |  |  | (.067) |  |  |  |
|  |  | 5 | 0.466 | 20 | 0.73 | 0.482 | 36 | 0.83 | -1.869 | 45 | 1 | -1.900 | 70 | 1 | 0.456 | 80 | 0.68 | 0.469 | 95 | 0.63 | 0.571 | 20 | 36 | 0.99 | -1.875 | 45 | 70 | 1 | 0.835 | 80 | 95 | 0.99 |
|  |  |  | (.066) |  |  | (.086) |  |  | (.092) |  |  | (.090) |  |  | (.077) |  |  | (.080) |  |  | (.086) |  |  |  | (.085) |  |  |  | (.092) |  |  |  |
|  | 0.5 | 10 | 0.704 | 20 | 0.02 | 0.650 | 36 | 0.01 | -1.871 | 45 | 1 | -1.857 | 70 | 1 | 0.645 | 80 | 0.06 | 0.623 | 95 | 0.03 | 0.651 | 20 | 36 | 0.12 | -1.974 | 45 | 70 | 1 | 0.789 | 80 | 95 | 0.63 |
|  |  |  | (.099) |  |  | NA |  |  | (.138) |  |  | (.128) |  |  | (.049) |  |  | (.011) |  |  | (.085) |  |  |  | (.129) |  |  |  | (.097) |  |  |  |
|  |  | 5 | 0.838 | 20 | 0.01 | 0.756 | 36 | 0.01 | -1.966 | 45 | 1 | -1.764 | 70 | 1 | NA | 80 | 0 | NA | 95 | 0 | 0.914 | 20 | 36 | 0.07 | -1.877 | 45 | 70 | 1 | 1.013 | 80 | 95 | 0.5 |
|  |  |  | NA |  |  | NA |  |  | (.191) |  |  | (.165) |  |  | NA |  |  | NA |  |  | (.060) |  |  |  | (.175) |  |  |  | (.131) |  |  |  |
|  | 0.2 | 10 | NA | 20 | 0 | NA | 36 | 0 | -1.887 | 45 | 0.97 | -1.798 | 70 | 0.98 | NA | 80 | 0 | NA | 95 | 0 | NA | 20 | 36 | 0 | -1.803 | 45 | 70 | 0.97 | 1.270 | 80 | 95 | 0.02 |
|  |  |  | NA |  |  | NA |  |  | (.249) |  |  | (.243) |  |  | NA |  |  | NA |  |  | NA |  |  |  | (.220) |  |  |  | (.151) |  |  |  |
|  |  | 5 | NA | 20 | 0 | NA | 36 | 0 | -1.750 | 45 | 0.7 | -1.785 | 70 | 0.74 | NA | 80 | 0 | NA | 95 | 0 | NA | 20 | 36 | 0 | -1.797 | 45 | 70 | 0.53 | 1.316 | 80 | 95 | 0.02 |
|  |  |  | NA |  |  | NA |  |  | (.295) |  |  | (.269) |  |  | NA |  |  | NA |  |  | NA |  |  |  | (.242) |  |  |  | (.016) |  |  |  |

Epistatic effects estimated in Z_6_ were multiplied by 2.
