# Supplementary material for: Genetic dissection of main and epistatic effects of QTL based on augmented triple test cross design
Source: PLoS One. 2017 Dec 14;12(12):e0189054. doi: 10.1371/journal.pone.0189054 (PMC5730204; doi:10.1371/journal.pone.0189054)
Supplement: S12 Supporting Information — (DOC) [file pone.0189054.s012.doc]

**Table 1. QTL mapping results for RIL, Z_1_, Z_2_ and Z_4_ in *II* hybrid.**

|  |  |  |  |  | RIL | |  | Z_1_ | |  | | Z_2_ | | |  | | Z_4_ (*i_aa_* – *i_dd_*) | | | Z_4_ (*i_ad_* - *i_da_*) | | |
| --- | --- | --- | --- | --- | --- | --- | --- | --- | --- | --- | --- | --- | --- | --- | --- | --- | --- | --- | --- | --- | --- | --- |
| Trait | Chr^a^ | Marker | Chr^a^ | Marker | beta (sd) | h^2^(%)^b^ |  | beta (sd) | h^2^(%) ^b^ | |  | | beta (sd) | h^2^(%) ^b^ | |  | | beta (sd) | h^2^(%) ^b^ | | beta (sd) | h^2^(%) ^b^ |
| HD | 12 | C996 |  |  |  |  |  | -2.09 (0.77) | 2.86 | |  | |  |  | |  | |  |  | |  |  |
| HD | 7 | RG678 | 7 | R1789 | 1.69 (0.43) | 4.93 |  |  |  | |  | |  |  | |  | |  |  | |  |  |
| HD | 8 | C347 | 13 | C56 |  |  |  | 2.63 (0.73) | 4.41 | |  | |  |  | |  | |  |  | |  |  |
| HD | 1 | R2632 | 10 | RM258 |  |  |  |  |  | |  | | 2.03 (0.49) | 5.91 | |  | |  |  | |  |  |
| HD | 2 | RZ599 | 11 | R3203 |  |  |  |  |  | |  | |  |  | |  | | -3.77 (0.89) | 6.05 | |  |  |
| HD | 3 | RM227 | 8 | C1121 |  |  |  |  |  | |  | |  |  | |  | | -3.11 (0.89) | 3.97 | |  |  |
| HD | 6 | Waxy | 6 | R2549 |  |  |  |  |  | |  | |  |  | |  | |  |  | | 1.66 (0.63) | 1.96 |
|  |  |  |  |  |  |  |  |  |  | |  | |  |  | |  | |  |  | |  |  |
| PH | 10 | RG561 |  |  | 1.27 (0.53) | 2.16 |  |  |  | |  | |  |  | |  | |  |  | |  |  |
| PH | 7 | RM70 |  |  |  |  |  | 1.92 (0.84) | 1.93 | |  | |  |  | |  | |  |  | |  |  |
| PH | 3 | R1925 |  |  |  |  |  |  |  | |  | | -1.86 (0.8) | 1.99 | |  | |  |  | |  |  |
| PH | 5 | RG360 | 8 | R1629 |  |  |  |  |  | |  | |  |  | |  | | -4.88 (1.73) | 2.36 | |  |  |
| PH | 6 | RM204 | 6 | C962 |  |  |  |  |  | |  | |  |  | |  | |  |  | | -3.34 (1.26) | 2.07 |
|  |  |  |  |  |  |  |  |  |  | |  | |  |  | |  | |  |  | |  |  |
| TP | 2 | RM53 |  |  |  |  |  |  |  | |  | | -0.38 (0.15) | 2.32 | |  | |  |  | |  |  |
| TP | 4 | R78 | 12 | C909B |  |  |  | -0.60 (0.16) | 4.58 | |  | |  |  | |  | |  |  | |  |  |
| TP | 4 | C2807 | 6 | P |  |  |  |  |  | |  | |  |  | |  | | 1.19 (0.31) | 5.84 | |  |  |
| TP | 2 | RG634 | 2 | R1738 |  |  |  |  |  | |  | |  |  | |  | |  |  | | 1.63 (0.6) | 2.16 |
|  |  |  |  |  |  |  |  |  |  | |  | |  |  | |  | |  |  | |  |  |
| PL | 6 | G342 |  |  |  |  |  | 0.30 (0.12) | 1.21 | |  | |  |  | |  | |  |  | |  |  |
| PL | 4 | G235 | 7 | RM70 |  |  |  | 0.60 (0.16) | 4.51 | |  | |  |  | |  | |  |  | |  |  |
| PL | 3 | C316 | 8 | RM25 |  |  |  |  |  | |  | |  |  | |  | | -0.82 (0.27) | 2.59 | |  |  |
| PL | 6 | RM204 | 12 | C909B |  |  |  |  |  | |  | |  |  | |  | |  |  | | 0.55 (0.21) | 2.06 |
|  |  |  |  |  |  |  |  |  |  | |  | |  |  | |  | |  |  | |  |  |
| FGPP | 1 | R2632 |  |  | 3.57 (1.2) | 3.45 |  |  |  | |  | |  |  | |  | |  |  | |  |  |
| FGPP | 5 | R3166 | 6 | RZ667 |  |  |  | -8.29 (1.73) | 7.67 | |  | |  |  | |  | |  |  | |  |  |
| FGPP | 8 | RM223 | 9 | RG570 |  |  |  |  |  | |  | | -3.70 (1.18) | 3.3 | |  | |  |  | |  |  |
| FGPP | 2 | RM48 | 14 | R1687 |  |  |  |  |  | |  | |  |  | |  | | 7.93 (2.72) | 2.64 | |  |  |
| FGPP | 8 | C1121 | 8 | RG978 |  |  |  |  |  | |  | |  |  | |  | |  |  | | 7.65 (3.13) | 1.81 |
|  |  |  |  |  |  |  |  |  |  | |  | |  |  | |  | |  |  | |  |  |
| SS | 12 | RM20b |  |  |  |  |  | -2.62 (0.91) | 3.21 | |  | |  |  | |  | |  |  | |  |  |
| SS | 2 | RZ324 | 11 | C1003B | 1.93 (0.58) | 3.71 |  |  |  | |  | |  |  | |  | |  |  | |  |  |
| SS | 6 | RG424 | 13 | C933 |  |  |  |  |  | |  | | 2.37 (0.64) | 4.45 | |  | |  |  | |  |  |
| SS | 2 | G1314a | 6 | R2549 |  |  |  |  |  | |  | |  |  | |  | | 5.88 (1.57) | 5.55 | |  |  |
| SS | 14 | C153B | 14 | C477 |  |  |  |  |  | |  | |  |  | |  | |  |  | | 14.02 (4.68) | 2.77 |
|  |  |  |  |  |  |  |  |  |  | |  | |  |  | |  | |  |  | |  |  |
| GD | 9 | RM201 |  |  | 0.10 (0.04) | 1.38 |  |  |  | |  | |  |  | |  | |  |  | |  |  |
| GD | 6 | R1014 |  |  | 0.10 (0.04) | 1.64 |  |  |  | |  | |  |  | |  | |  |  | |  |  |
| GD | 5 | R3166 | 6 | RZ667 |  |  |  | -0.31 (0.07) | 7.33 | |  | |  |  | |  | |  |  | |  |  |
| GD | 1 | C161 | 1 | RM243 |  |  |  |  |  | |  | |  |  | |  | | 0.43 (0.1) | 5.79 | |  |  |
| GD | 2 | RM48 | 14 | R1687 |  |  |  |  |  | |  | |  |  | |  | | 0.34 (0.1) | 4.26 | |  |  |
| GD | 2 | RZ324 | 7 | RM234 |  |  |  |  |  | |  | |  |  | |  | |  |  | | 0.23 (0.07) | 3.67 |
|  |  |  |  |  |  |  |  |  |  | |  | |  |  | |  | |  |  | |  |  |
| KGW | 3 | C1176 |  |  |  |  |  |  |  | |  | | -0.33 (0.11) | 2.48 | |  | |  |  | |  |  |
| KGW | 8 | L363A |  |  |  |  |  |  |  | |  | | -0.22 (0.09) | 1.13 | |  | |  |  | |  |  |
| KGW | 3 | R19 | 10 | RM239 | -0.66 (0.17) | 4.94 |  |  |  | |  | |  |  | |  | |  |  | |  |  |
| KGW | 5 | RG360 | 8 | C347 |  |  |  |  |  | |  | | -0.39 (0.12) | 3.47 | |  | |  |  | |  |  |
| KGW | 8 | RM25 | 9 | RZ404 |  |  |  | -0.99 (0.23) | 6.5 | |  | |  |  | |  | |  |  | |  |  |
| KGW | 1 | RG101 | 4 | RM241 |  |  |  |  |  | |  | |  |  | |  | | 0.67 (0.24) | 1.88 | |  |  |
| KGW | 1 | G393 | 1 | R2201 |  |  |  |  |  | |  | |  |  | |  | |  |  | | -1.49 (0.62) | 1.69 |
|  |  |  |  |  |  |  |  |  |  | |  | |  |  | |  | |  |  | |  |  |
| YD | 10 | C153A |  |  |  |  |  | -1.50 (0.56) | 2.73 | |  | |  |  | |  | |  |  | |  |  |
| YD | 7 | R1789 |  |  |  |  |  |  |  | |  | | 1.67 (0.48) | 3.67 | |  | |  |  | |  |  |
| YD | 4 | C1016 | 8 | C483 | 1.06 (0.23) | 7.49 |  |  |  | |  | |  |  | |  | |  |  | |  |  |
| YD | 4 | C2807 | 14 | C477 |  |  |  |  |  | |  | | -1.94 (0.49) | 5.33 | |  | |  |  | |  |  |
| YD | 8 | R1394 | 9 | RM215 |  |  |  |  |  | |  | |  |  | |  | | 3.97 (1.18) | 4.48 | |  |  |
| YD | 9 | R1952b | 10 | C153A |  |  |  |  |  | |  | |  |  | |  | |  |  | | -2.75 (0.78) | 3.86 |
| YD | 11 | L1044 | 11 | Y6854L |  |  |  |  |  | |  | |  |  | |  | |  |  | | -9.06 (2.07) | 7.09 |

^a^ Chromosome where the detected QTL located in.

^b^ Variation contributed by QTL or digenic interaction.

**Table 2. QTL mapping results for RIL, Z_1_, Z_2_ and Z_4_ in *IJ* hybrid.**

|  |  |  |  | |  | | RIL | | | | |  | | Z_1_ | | | | |  | Z_2_ | | | | | | |  | Z_4_ (*i_aa_* – *i_dd_*) | | | | | | | Z_4_ (*i_ad_* - *i_da_*) | | | | | |
| --- | --- | --- | --- | --- | --- | --- | --- | --- | --- | --- | --- | --- | --- | --- | --- | --- | --- | --- | --- | --- | --- | --- | --- | --- | --- | --- | --- | --- | --- | --- | --- | --- | --- | --- | --- | --- | --- | --- | --- | --- |
| Trait | Chr^a^ | Marker | | Chr^a^ | | Marker | | beta (sd) | | h^2^(%) ^b^ | | |  | | beta (sd) | | h^2^(%) ^b^ | | | |  | | beta (sd) | | h^2^(%) ^b^ | | | |  | | beta (sd) | | h^2^(%) ^b^ | | | beta (sd) | | h^2^(%) ^b^ | | |
| HD | 1 | XNPB302 | |  | |  | | -0.82 (0.21) | | 1.83 | | |  | |  | |  | | | |  | |  | |  | | | |  | |  | |  | | |  | |  | | |
| HD | 3 | RG510 | |  | |  | | | 0.85 (0.22) | | 1.69 | | |  | |  | |  | | | |  | |  | |  | | | |  | |  | |  | | |  | |  |  |
| HD | 3 | CDO1081 | |  | |  | | | 1.62 (0.22) | | 7.08 | | |  | | -0.72 (0.19) | | 2.94 | | | |  | |  | |  | | | |  | |  | |  | | |  | |  |  |
| HD | 8 | RG333 | |  | |  | | | -3.46 (0.36) | | 27.48 | | |  | | 2.73 (0.21) | | 36.58 | | | |  | | 0.96 (0.17) | | 10.93 | | | |  | |  | |  | | |  | |  |  |
| HD | 8 | RZ562 | |  | |  | | | -1.18 (0.34) | | 3.29 | | |  | |  | |  | | | |  | |  | |  | | | |  | |  | |  | | |  | |  |  |
| HD | 11 | CDO534 | |  | |  | | | 0.83 (0.20) | | 1.85 | | |  | |  | |  | | | |  | |  | |  | | | |  | |  | |  | | |  | |  |  |
| HD | 1 | RG811 | |  | |  | | |  | |  | | |  | | 0.73 (0.19) | | 3.04 | | | |  | |  | |  | | | |  | |  | |  | | |  | |  |  |
| HD | 3 | XNPB249 | |  | |  | | |  | |  | | |  | | -0.83 (0.19) | | 3.72 | | | |  | |  | |  | | | |  | |  | |  | | |  | |  |  |
| HD | 7 | RG711 | |  | |  | | |  | |  | | |  | | -0.6 (0.18) | | 1.95 | | | |  | |  | |  | | | |  | |  | |  | | |  | |  |  |
| HD | 4 | RG908 | | 8 | | RZ562 | | | 1.20 (0.22) | | 3.65 | | |  | |  | |  | | | |  | |  | |  | | | |  | |  | |  | | |  | |  |  |
| HD | 5 | RZ556 | | 8 | | RZ562 | | |  | |  | | |  | | 1.11 (0.28) | | 1.76 | | | |  | |  | |  | | | |  | |  | |  | | |  | |  |  |
| HD | 8 | RZ562 | | 9 | | RG667 | | |  | |  | | |  | |  | |  | | | |  | |  | |  | | | |  | | -2.02 (0.51) | | 7.01 | | |  | |  |  |
| HD | 2 | TW500 | | 5 | | RG480 | | |  | |  | | |  | |  | |  | | | |  | |  | |  | | | |  | |  | |  | | | -0.86 (0.26) | | 2.55 |  |
| HD | 6 | RZ450 | | 6 | | CDO204 | | |  | |  | | |  | |  | |  | | | |  | |  | |  | | | |  | |  | |  | | | -13.32 (2.9) | | 6.30 |  |
| HD | 8 | RG333 | | 11 | | CDO534 | | |  | |  | | |  | |  | |  | | | |  | |  | |  | | | |  | |  | |  | | | -2.13 (0.3) | | 15.90 |  |
|  |  |  | |  | |  | | |  | |  | | |  | |  | |  | | | |  | |  | |  | | | |  | |  | |  | | |  | |  |  |
| PH | 1 | RZ776 | |  | |  | | | -1.89 (0.37) | | 4.51 | | |  | |  | |  | | | |  | |  | |  | | | |  | |  | |  | | |  | |  |  |
| PH | 2 | RG544 | |  | |  | | | -1.03 (0.34) | | 1.35 | | |  | |  | |  | | | |  | |  | |  | | | |  | |  | |  | | |  | |  |  |
| PH | 3 | CDO87 | |  | |  | | | 2.76 (0.42) | | 8.64 | | |  | |  | |  | | | |  | |  | |  | | | |  | |  | |  | | |  | |  |  |
| PH | 3 | XNPB249 | |  | |  | | | -2.18 (0.41) | | 5.73 | | |  | |  | |  | | | |  | |  | |  | | | |  | |  | |  | | |  | |  |  |
| PH | 3 | RZ16 | |  | |  | | | -1.22 (0.35) | | 1.93 | | |  | |  | |  | | | |  | |  | |  | | | |  | |  | |  | | |  | |  |  |
| PH | 6 | RZ682 | |  | |  | | | 2.25 (0.37) | | 6.05 | | |  | |  | |  | | | |  | |  | |  | | | |  | |  | |  | | |  | |  |  |
| PH | 8 | RG333 | |  | |  | | | -2.22 (0.37) | | 5.54 | | |  | | 3.47 (0.7) | | 8.47 | | | |  | |  | |  | | | |  | |  | |  | | |  | |  |  |
| PH | 7 | CDO533 | |  | |  | | |  | |  | | |  | |  | |  | | | |  | | 1.48 (0.47) | | 4.29 | | | |  | |  | |  | | |  | |  |  |
| PH | 1 | XNPB370 | | 7 | | CDO497 | | | 1.64 (0.37) | | 3.35 | | |  | |  | |  | | | |  | |  | |  | | | |  | |  | |  | | |  | |  |  |
| PH | 1 | RG233 | | 12 | | RG574 | | | -1.40 (0.36) | | 2.4 | | |  | |  | |  | | | |  | |  | |  | | | |  | |  | |  | | |  | |  |  |
| PH | 2 | RG152 | | 4 | | XNPB271 | | | -2.33 (0.37) | | 6.79 | | |  | |  | |  | | | |  | |  | |  | | | |  | |  | |  | | |  | |  |  |
| PH | 2 | TW500 | | 7 | | RZ509 | | | -1.16 (0.34) | | 1.72 | | |  | |  | |  | | | |  | |  | |  | | | |  | |  | |  | | |  | |  |  |
| PH | 4 | RZ602 | | 8 | | RZ66 | | | 1.43 (0.36) | | 2.47 | | |  | |  | |  | | | |  | |  | |  | | | |  | |  | |  | | |  | |  |  |
| PH | 6 | CDO204 | | 11 | | XNPB320 | | | -2.00 (0.38) | | 5.04 | | |  | |  | |  | | | |  | |  | |  | | | |  | |  | |  | | |  | |  |  |
| PH | 6 | RZ667 | | 9 | | RG662 | | | -1.41 (0.36) | | 2.52 | | |  | |  | |  | | | |  | |  | |  | | | |  | |  | |  | | |  | |  |  |
| PH | 2 | RZ987 | | 10 | | RZ892 | | |  | |  | | |  | | -5.23 (1.13) | | 5.59 | | | |  | |  | |  | | | |  | |  | |  | | |  | |  |  |
| PH | 2 | RG152 | | 4 | | XNPB271 | | |  | |  | | |  | | 6.01 (0.98) | | 7.36 | | | |  | |  | |  | | | |  | |  | |  | | |  | |  |  |
| PH | 2 | TW500 | | 3 | | RG510 | | |  | |  | | |  | |  | |  | | | |  | |  | |  | | | |  | | -2.69 (0.9) | | 3.11 | | |  | |  |  |
| PH | 1 | RG173 | | 2 | | TW500 | | |  | |  | | |  | |  | |  | | | |  | |  | |  | | | |  | |  | |  | | | 2.29 (0.65) | | 4.36 |  |
| PH | 4 | RG449 | | 7 | | RG711 | | |  | |  | | |  | |  | |  | | | |  | |  | |  | | | |  | |  | |  | | | 3.26 (0.7) | | 8.05 |  |
| PH | 5 | RZ390 | | 9 | | RZ12 | | |  | |  | | |  | |  | |  | | | |  | |  | |  | | | |  | |  | |  | | | 1.8 (0.65) | | 2.46 |  |
| PH | 5 | RZ70 | | 8 | | RZ562 | | |  | |  | | |  | |  | |  | | | |  | |  | |  | | | |  | |  | |  | | | -3.35 (0.65) | | 9.81 |  |
|  |  |  | |  | |  | | |  | |  | | |  | |  | |  | | | |  | |  | |  | | | |  | |  | |  | | |  | |  |  |
| TP | 4 | RG214 | |  | |  | | | 0.21 (0.05) | | 5.12 | | |  | |  | |  | | | |  | |  | |  | | | |  | |  | |  | | |  | |  |  |
| TP | 4 | RG908 | |  | |  | | | -0.10 (0.04) | | 1.09 | | |  | |  | |  | | | |  | |  | |  | | | |  | |  | |  | | |  | |  |  |
| TP | 5 | CDO1160 | |  | |  | | | 0.11 (0.04) | | 1.53 | | |  | |  | |  | | | |  | |  | |  | | | |  | |  | |  | | |  | |  |  |
| TP | 1 | RG541 | | 7 | | RZ626 | | |  | |  | | |  | | 0.34 (0.1) | | 2.42 | | | |  | |  | |  | | | |  | |  | |  | | |  | |  |  |
| TP | 9 | RZ422 | | 11 | | XNPB179 | | |  | |  | | |  | | -0.41 (0.11) | | 3.26 | | | |  | |  | |  | | | |  | | 0.5 (0.15) | | 3.75 | | |  | |  |  |
| TP | 2 | RG555 | | 7 | | CDO405 | | |  | |  | | |  | |  | |  | | | |  | | 0.38 (0.09) | | 4.39 | | | |  | |  | |  | | |  | |  |  |
| TP | 6 | RZ450 | | 9 | | RZ12 | | |  | |  | | |  | |  | |  | | | |  | | -0.29 (0.08) | | 2.65 | | | |  | |  | |  | | |  | |  |  |
| TP | 2 | TW500 | | 6 | | RZ828 | | |  | |  | | |  | |  | |  | | | |  | |  | |  | | | |  | | 0.58 (0.16) | | 5.12 | | |  | |  |  |
| TP | 3 | RG1356 | | 6 | | RG213 | | |  | |  | | |  | |  | |  | | | |  | |  | |  | | | |  | |  | |  | | | 0.24 (0.09) | | 1.57 |  |
| TP | 4 | RG908 | | 9 | | XNPB295 | | |  | |  | | |  | |  | |  | | | |  | |  | |  | | | |  | |  | |  | | | -0.19 (0.09) | | 1.11 |  |
|  |  |  | |  | |  | | |  | |  | | |  | |  | |  | | | |  | |  | |  | | | |  | |  | |  | | |  | |  |  |
| PL | 1 | RG462 | |  | |  | | | 0.26 (0.10) | | 1.91 | | |  | |  | |  | | | |  | |  | |  | | | |  | |  | |  | | |  | |  |  |
| PL | 1 | RZ276 | |  | |  | | | -0.22 (0.09) | | 1.44 | | |  | |  | |  | | | |  | |  | |  | | | |  | |  | |  | | |  | |  |  |
| PL | 2 | XNPB132 | |  | |  | | | 0.24 (0.09) | | 1.66 | | |  | |  | |  | | | |  | |  | |  | | | |  | |  | |  | | |  | |  |  |
| PL | 5 | RZ495 | |  | |  | | | -0.24 (0.10) | | 1.72 | | |  | |  | |  | | | |  | |  | |  | | | |  | |  | |  | | |  | |  |  |
| PL | 5 | RG480 | |  | |  | | | 0.28 (0.11) | | 2.3 | | |  | |  | |  | | | |  | |  | |  | | | |  | |  | |  | | |  | |  |  |
| PL | 5 | RG697 | |  | |  | | | -0.25 (0.10) | | 1.8 | | |  | |  | |  | | | |  | |  | |  | | | |  | |  | |  | | |  | |  |  |
| PL | 6 | RZ144 | |  | |  | | | -0.20 (0.08) | | 1.17 | | |  | |  | |  | | | |  | |  | |  | | | |  | |  | |  | | |  | |  |  |
| PL | 7 | XNPB20 | |  | |  | | | -0.42 (0.09) | | 4.97 | | |  | |  | |  | | | |  | |  | |  | | | |  | |  | |  | | |  | |  |  |
| PL | 7 | RG528 | |  | |  | | | -0.21 (0.09) | | 1.26 | | |  | |  | |  | | | |  | |  | |  | | | |  | |  | |  | | |  | |  |  |
| PL | 8 | XNPB56 | |  | |  | | | 0.30 (0.09) | | 2.74 | | |  | |  | |  | | | |  | |  | |  | | | |  | |  | |  | | |  | |  |  |
| PL | 9 | RG358 | |  | |  | | | 0.21 (0.09) | | 1.17 | | |  | |  | |  | | | |  | |  | |  | | | |  | |  | |  | | |  | |  |  |
| PL | 12 | RG869 | |  | |  | | | -0.25 (0.09) | | 1.86 | | |  | |  | |  | | | |  | |  | |  | | | |  | |  | |  | | |  | |  |  |
| PL | 1 | XNPB302 | | 9 | | RZ422 | | | -0.34 (0.09) | | 3.42 | | |  | |  | |  | | | |  | |  | |  | | | |  | |  | |  | | |  | |  |  |
| PL | 8 | RG136 | | 11 | | RZ638 | | | -0.35 (0.09) | | 3.53 | | |  | |  | |  | | | |  | |  | |  | | | |  | |  | |  | | |  | |  |  |
| PL | 5 | RZ296 | | 11 | | CDO534 | | |  | |  | | |  | | 0.44 (0.17) | | 1.04 | | | |  | |  | |  | | | |  | |  | |  | | |  | |  |  |
| PL | 4 | RZ262 | | 12 | | XNPB189 | | |  | |  | | |  | |  | |  | | | |  | |  | |  | | | |  | | 0.89 (0.22) | | 6.30 | | |  | |  |  |
| PL | 4 | RG449 | | 11 | | CDO534 | | |  | |  | | |  | |  | |  | | | |  | |  | |  | | | |  | | -0.63 (0.21) | | 3.11 | | |  | |  |  |
| PL | 9 | RZ404 | | 12 | | RG98 | | |  | |  | | |  | |  | |  | | | |  | |  | |  | | | |  | |  | |  | | | 0.61 (0.18) | | 5.07 |  |
|  |  |  | |  | |  | | |  | |  | | |  | |  | |  | | | |  | |  | |  | | | |  | |  | |  | | |  | |  |  |
| FGPP | 4 | RG214 | |  | |  | | | -7.28 (1.17) | | 13.06 | | |  | |  | |  | | | |  | |  | |  | | | |  | |  | |  | | |  | |  |  |
| FGPP | 5 | RG360 | |  | |  | | | 4.98 (1.15) | | 5.95 | | |  | |  | |  | | | |  | |  | |  | | | |  | |  | |  | | |  | |  |  |
| FGPP | 3 | CDO1081 | |  | |  | | | -5.60 (1.13) | | 7.97 | | |  | | 6.12 (1.28) | | 8.58 | | | |  | |  | |  | | | |  | |  | |  | | |  | |  |  |
| FGPP | 1 | RG541 | | 4 | | XNPB271 | | |  | |  | | |  | |  | |  | | | |  | | 6.34 (1.38) | | 4.69 | | | |  | |  | |  | | |  | |  |  |
| FGPP | 1 | RG469 | | 5 | | RZ390 | | |  | |  | | |  | |  | |  | | | |  | |  | |  | | | |  | | 6.35 (1.56) | | 2.43 | | |  | |  |  |
| FGPP | 1 | RZ776 | | 8 | | RG333 | | |  | |  | | |  | |  | |  | | | |  | |  | |  | | | |  | | 8.35 (1.57) | | 4.21 | | |  | |  |  |
| FGPP | 1 | RG375 | | 10 | | RZ811 | | |  | |  | | |  | |  | |  | | | |  | |  | |  | | | |  | | -10.93 (1.66) | | 7.20 | | |  | |  |  |
| FGPP | 2 | RZ913 | | 2 | | RG544 | | |  | |  | | |  | |  | |  | | | |  | |  | |  | | | |  | | 5.41 (1.54) | | 1.78 | | |  | |  |  |
| FGPP | 2 | CDO395 | | 7 | | CDO405 | | |  | |  | | |  | |  | |  | | | |  | |  | |  | | | |  | | 9.95 (1.63) | | 6.01 | | |  | |  |  |
| FGPP | 2 | CDO395 | | 8 | | RG333 | | |  | |  | | |  | |  | |  | | | |  | |  | |  | | | |  | | 4.29 (1.45) | | 1.11 | | |  | |  |  |
| FGPP | 2 | RZ987 | | 6 | | WAXY | | |  | |  | | |  | |  | |  | | | |  | |  | |  | | | |  | | 4.57 (1.46) | | 1.27 | | |  | |  |  |
| FGPP | 2 | RZ987 | | 10 | | RZ892 | | |  | |  | | |  | |  | |  | | | |  | |  | |  | | | |  | | -8.04 (1.6) | | 3.90 | | |  | |  |  |
| FGPP | 2 | XNPB132 | | 10 | | RZ811 | | |  | |  | | |  | |  | |  | | | |  | |  | |  | | | |  | | -6.85 (1.57) | | 2.82 | | |  | |  |  |
| FGPP | 2 | RG544 | | 5 | | RG480 | | |  | |  | | |  | |  | |  | | | |  | |  | |  | | | |  | | -9.29 (1.67) | | 5.20 | | |  | |  |  |
| FGPP | 2 | TW500 | | 8 | | RZ562 | | |  | |  | | |  | |  | |  | | | |  | |  | |  | | | |  | | 10.39 (1.63) | | 6.55 | | |  | |  |  |
| FGPP | 3 | XNPB249 | | 8 | | RG136 | | |  | |  | | |  | |  | |  | | | |  | |  | |  | | | |  | | -4.65 (1.5) | | 1.31 | | |  | |  |  |
| FGPP | 3 | RZ16 | | 10 | | RZ561 | | |  | |  | | |  | |  | |  | | | |  | |  | |  | | | |  | | 6.01 (1.52) | | 2.18 | | |  | |  |  |
| FGPP | 4 | CDO456 | | 11 | | XNPB320 | | |  | |  | | |  | |  | |  | | | |  | |  | |  | | | |  | | -7.22 (1.56) | | 3.16 | | |  | |  |  |
| FGPP | 5 | RG573 | | 9 | | RG667 | | |  | |  | | |  | |  | |  | | | |  | |  | |  | | | |  | | -7.39 (1.57) | | 3.28 | | |  | |  |  |
| FGPP | 6 | RG433 | | 8 | | RZ28 | | |  | |  | | |  | |  | |  | | | |  | |  | |  | | | |  | | 4.2 (1.43) | | 1.07 | | |  | |  |  |
| FGPP | 7 | CDO405 | | 10 | | RZ561 | | |  | |  | | |  | |  | |  | | | |  | |  | |  | | | |  | | -9.19 (1.61) | | 5.08 | | |  | |  |  |
| FGPP | 1 | RG375 | | 12 | | XNPB316 | | |  | |  | | |  | |  | |  | | | |  | |  | |  | | | |  | |  | |  | | | -5.23 (1.69) | | 3.23 |  |
| FGPP | 1 | CDO962 | | 2 | | RZ987 | | |  | |  | | |  | |  | |  | | | |  | |  | |  | | | |  | |  | |  | | | 5.07 (1.59) | | 2.98 |  |
| FGPP | 1 | RG173 | | 5 | | RG480 | | |  | |  | | |  | |  | |  | | | |  | |  | |  | | | |  | |  | |  | | | -7.2 (1.85) | | 4.52 |  |
| FGPP | 3 | CDO1081 | | 12 | | RZ816 | | |  | |  | | |  | |  | |  | | | |  | |  | |  | | | |  | |  | |  | | | 3.7 (1.51) | | 1.54 |  |
| FGPP | 4 | CDO456 | | 6 | | XNPB317 | | |  | |  | | |  | |  | |  | | | |  | |  | |  | | | |  | |  | |  | | | 3.91 (1.52) | | 1.76 |  |
| FGPP | 7 | RG528 | | 11 | | RZ638 | | |  | |  | | |  | |  | |  | | | |  | |  | |  | | | |  | |  | |  | | | -7.35 (1.76) | | 5.27 |  |
| FGPP | 8 | RG333 | | 9 | | RZ927 | | |  | |  | | |  | |  | |  | | | |  | |  | |  | | | |  | |  | |  | | | -9.07 (1.77) | | 9.17 |  |
| FGPP | 9 | XNPB103 | | 12 | | RG901 | | |  | |  | | |  | |  | |  | | | |  | |  | |  | | | |  | |  | |  | | | -7.92 (1.77) | | 6.85 |  |
| FGPP | 9 | RZ12 | | 9 | | RG667 | | |  | |  | | |  | |  | |  | | | |  | |  | |  | | | |  | |  | |  | | | 15.39 (3.92) | | 4.74 |  |
| FGPP | 12 | RZ816 | | 12 | | XNPB189 | | |  | |  | | |  | |  | |  | | | |  | |  | |  | | | |  | |  | |  | | | -14.13 (4.51) | | 2.74 |  |
|  |  |  | |  | |  | | |  | |  | | |  | |  | |  | | | |  | |  | |  | | | |  | |  | |  | | |  | |  |  |
| SS | 5 | RG360 | |  | |  | | | 2.65 (0.74) | | 5.68 | | |  | |  | |  | | | |  | |  | |  | | | |  | |  | |  | | |  | |  |  |
| SS | 1 | XNPB302 | | 10 | | RZ811 | | |  | |  | | |  | |  | |  | | | |  | | -2.09 (0.69) | | 1.66 | | | |  | |  | |  | | |  | |  |  |
| SS | 2 | RG544 | | 7 | | RG711 | | |  | |  | | |  | |  | |  | | | |  | |  | |  | | | |  | | 6.08 (1.56) | | 6.81 | | |  | |  |  |
| SS | 7 | RG528 | | 10 | | RZ400 | | |  | |  | | |  | |  | |  | | | |  | |  | |  | | | |  | |  | |  | | | -4.09 (1.1) | | 5.15 |  |
|  |  |  | |  | |  | | |  | |  | | |  | |  | |  | | | |  | |  | |  | | | |  | |  | |  | | |  | |  |  |
| GD | 3 | RZ993 | |  | |  | | | -0.11 (0.04) | | 1.66 | | |  | |  | |  | | | |  | |  | |  | | | |  | |  | |  | | |  | |  |  |
| GD | 4 | RZ590 | |  | |  | | | -0.37 (0.05) | | 16.7 | | |  | |  | |  | | | |  | |  | |  | | | |  | |  | |  | | |  | |  |  |
| GD | 10 | RZ561 | |  | |  | | | 0.14 (0.05) | | 2.17 | | |  | |  | |  | | | |  | |  | |  | | | |  | |  | |  | | |  | |  |  |
| GD | 2 | RZ913 | | 2 | | RG634 | | | -0.16 (0.05) | | 3.24 | | |  | |  | |  | | | |  | |  | |  | | | |  | |  | |  | | |  | |  |  |
| GD | 6 | RG162 | |  | |  | | |  | |  | | |  | |  | |  | | | |  | | -0.16 (0.05) | | 3.20 | | | |  | |  | |  | | |  | |  |  |
| GD | 1 | RG375 | | 10 | | RZ811 | | |  | |  | | |  | | 0.37 (0.11) | | 2.01 | | | |  | |  | |  | | | |  | |  | |  | | |  | |  |  |
| GD | 2 | CDO395 | | 7 | | CDO405 | | |  | |  | | |  | |  | |  | | | |  | |  | |  | | | |  | | 0.65 (0.17) | | 6.52 | | |  | |  |  |
| GD | 2 | RZ599 | | 6 | | RZ965 | | |  | |  | | |  | |  | |  | | | |  | |  | |  | | | |  | | 0.5 (0.15) | | 3.77 | | |  | |  |  |
| GD | 4 | RZ590 | | 9 | | RZ12 | | |  | |  | | |  | |  | |  | | | |  | |  | |  | | | |  | |  | |  | | | -0.45 (0.11) | | 6.35 |  |
|  |  |  | |  | |  | | |  | |  | | |  | |  | |  | | | |  | |  | |  | | | |  | |  | |  | | |  | |  |  |
| KGW | 8 | RG333 | |  | |  | | | 2.86 (0.41) | | 14.55 | | |  | |  | |  | | | |  | |  | |  | | | |  | |  | |  | | |  | |  |  |
| KGW | 5 | RZ296 | |  | |  | | |  | |  | | |  | | 0.91 (0.27) | | 5.18 | | | |  | |  | |  | | | |  | |  | |  | | |  | |  |  |
| KGW | 5 | CDO202 | |  | |  | | |  | |  | | |  | |  | |  | | | |  | | -0.41 (0.18) | | 1.68 | | | |  | |  | |  | | |  | |  |  |
| KGW | 3 | CDO1081 | | 8 | | RG333 | | | -1.57 (0.39) | | 5 | | |  | |  | |  | | | |  | |  | |  | | | |  | |  | |  | | |  | |  |  |
| KGW | 2 | RG555 | | 9 | | RG358 | | |  | |  | | |  | |  | |  | | | |  | |  | |  | | | |  | | -2.67 (0.92) | | 233796.55 | | |  | |  |  |
| KGW | 4 | RG908 | | 8 | | RG333 | | |  | |  | | |  | |  | |  | | | |  | |  | |  | | | |  | |  | |  | | | 3.68 (0.61) | | 12.85 |  |
|  |  |  | |  | |  | | |  | |  | | |  | |  | |  | | | |  | |  | |  | | | |  | |  | |  | | |  | |  |  |
| YD | 3 | CDO1081 | |  | |  | | | -445.31 (109.02) | | 6.03 | | |  | |  | |  | | | |  | |  | |  | | | |  | |  | |  | | |  | |  |  |
| YD | 8 | RG333 | |  | |  | | | 557.91 (115.72) | | 8.09 | | |  | |  | |  | | | |  | |  | |  | | | |  | |  | |  | | |  | |  |  |
| YD | 8 | RZ562 | |  | |  | | |  | |  | | |  | |  | |  | | | |  | | -191.74 (58.46) | | 4.17 | | | |  | |  | |  | | |  | |  |  |
| YD | 2 | RZ825 | | 5 | | RG480 | | |  | |  | | |  | |  | |  | | | |  | |  | |  | | | |  | | -756.33 (228.48) | | 3.84 | | |  | |  |  |
| YD | 7 | CDO405 | | 10 | | RZ400 | | |  | |  | | |  | |  | |  | | | |  | |  | |  | | | |  | | -1088.35 (251.79) | | 7.86 | | |  | |  |  |
| YD | 4 | RG908 | | 8 | | RG333 | | |  | |  | | |  | |  | |  | | | |  | |  | |  | | | |  | |  | |  | | | 963.1 (162.74) | | 14.12 |  |

^a^ See footnote of Table 1.

^b^ See footnote of Table 1
