# Supplementary figures and images for: Genetic dissection of main and epistatic effects of QTL based on augmented triple test cross design
Source: PLoS One. 2017 Dec 14;12(12):e0189054. doi: 10.1371/journal.pone.0189054 (PMC5730204; doi:10.1371/journal.pone.0189054)

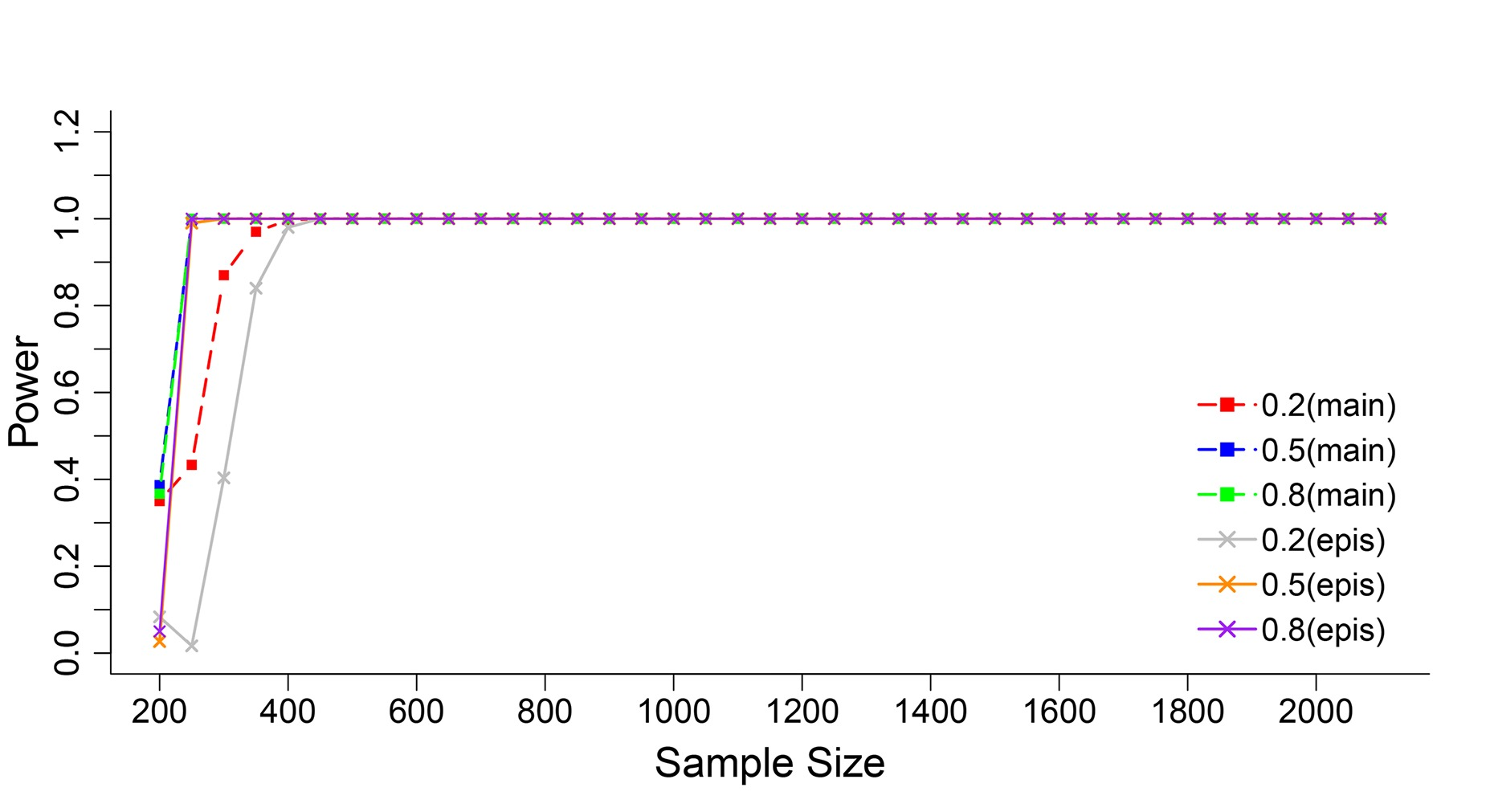

Supplement: S1 Fig — (TIF) [file pone.0189054.s013.tif]

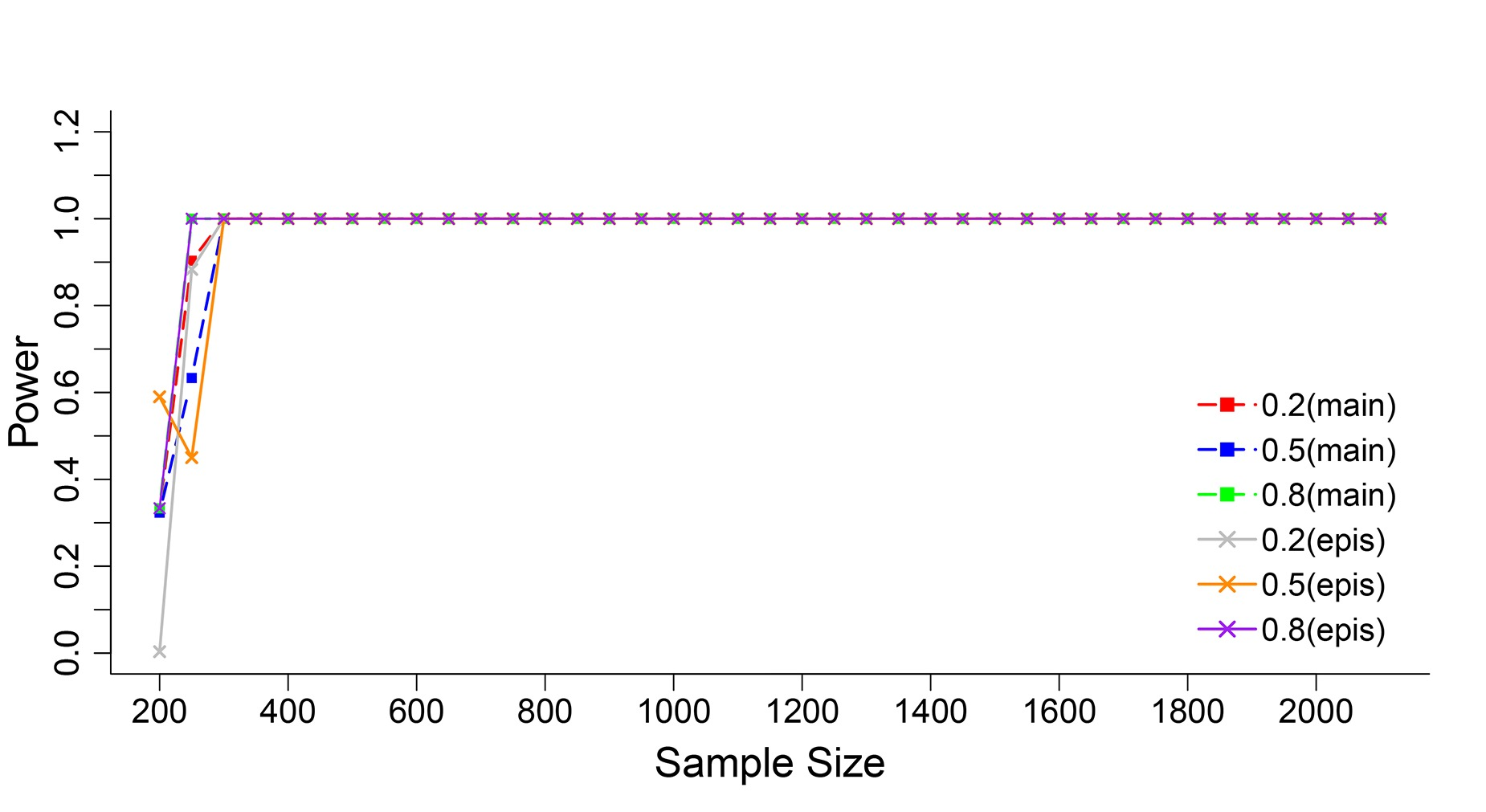

Supplement: S2 Fig — (TIF) [file pone.0189054.s014.tif]

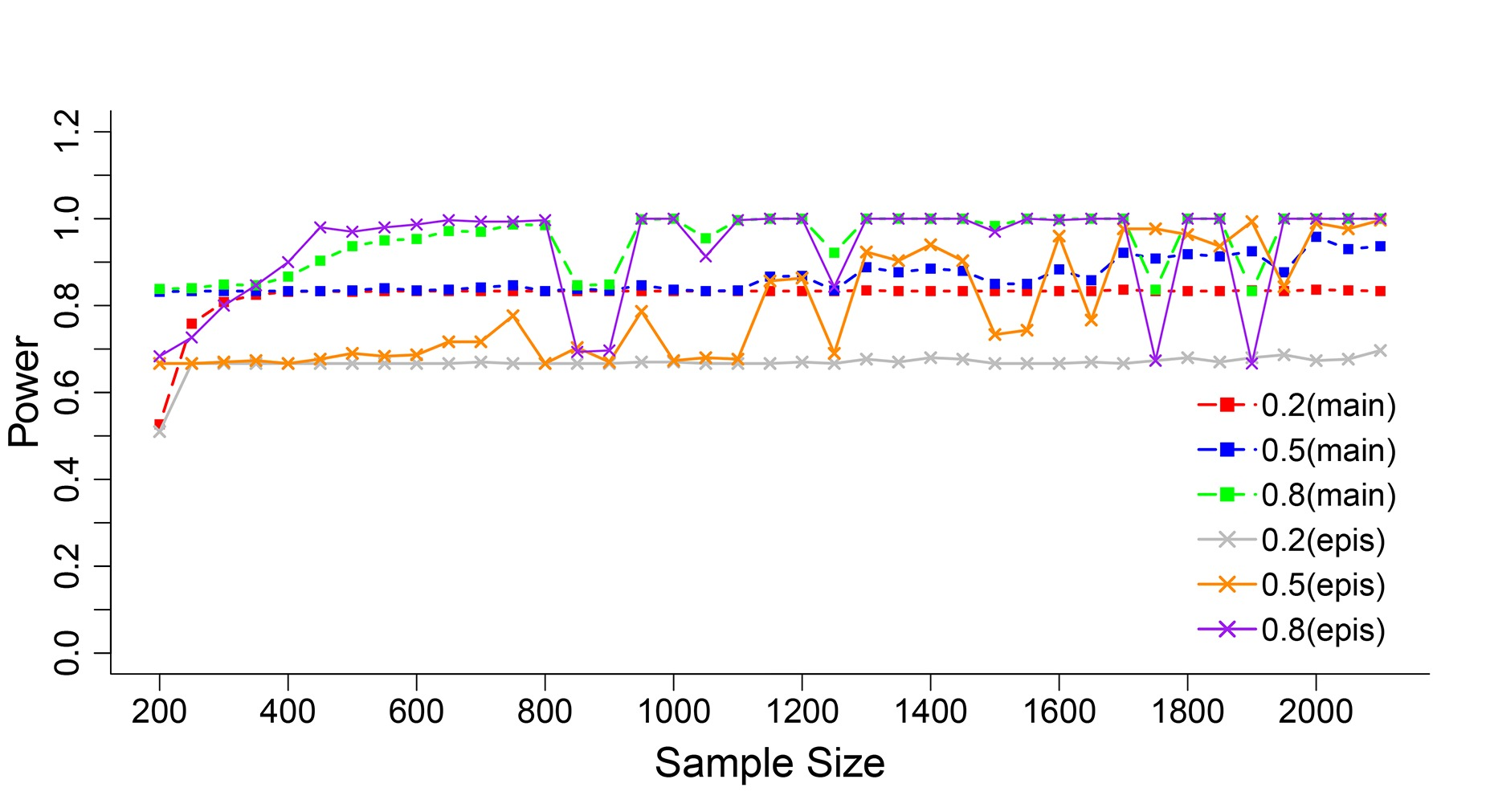

Supplement: S3 Fig — (TIF) [file pone.0189054.s015.tif]

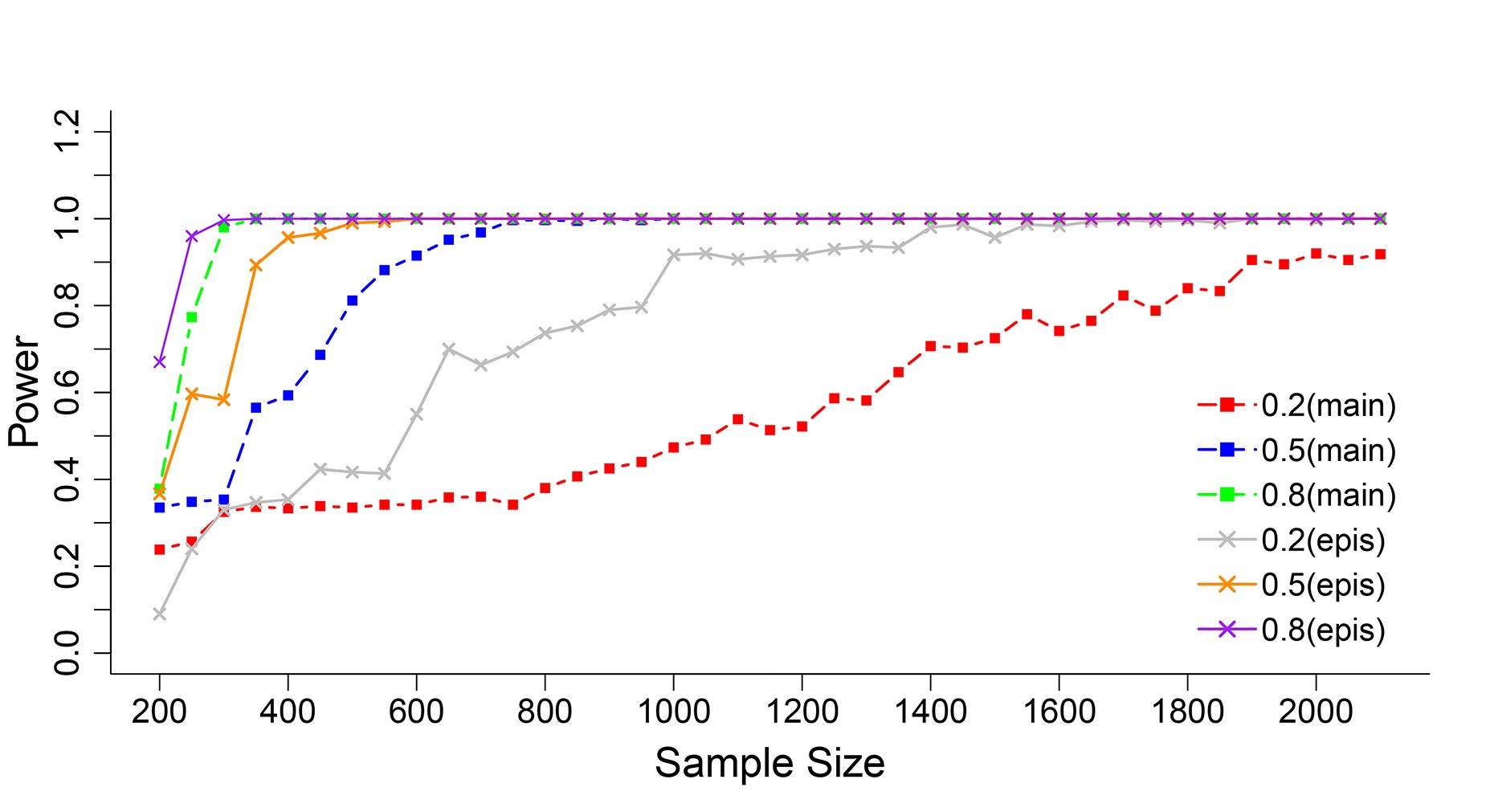

Supplement: S4 Fig — (TIF) [file pone.0189054.s016.tif]
